# Tomato genomic prediction for good performance under high-temperature and identification of loci involved in thermotolerance response

**DOI:** 10.1038/s41438-021-00647-3

**Published:** 2021-10-01

**Authors:** Elisa Cappetta, Giuseppe Andolfo, Anna Guadagno, Antonio Di Matteo, Amalia Barone, Luigi Frusciante, Maria Raffaella Ercolano

**Affiliations:** 1 Department of Agricultural Sciences, University of Naples Federico II, Via Università 100, 80055 Portici, Naples Italy; 2 Present Address: Institute of Bioscience and BioResources, National Research Council, Via Università 100, 80055 Portici, Italy

**Keywords:** Plant breeding, Abiotic

## Abstract

Many studies showed that few degrees above tomato optimum growth temperature threshold can lead to serious loss in production. Therefore, the development of innovative strategies to obtain tomato cultivars with improved yield under high temperature conditions is a main goal both for basic genetic studies and breeding activities. In this paper, a F4 segregating population was phenotypically evaluated for quantitative and qualitative traits under heat stress conditions. Moreover, a genotyping by sequencing (GBS) approach has been employed for building up genomic selection (GS) models both for yield and soluble solid content (SCC). Several parameters, including training population size, composition and marker quality were tested to predict genotype performance under heat stress conditions. A good prediction accuracy for the two analyzed traits (0.729 for yield production and 0.715 for SCC) was obtained. The predicted models improved the genetic gain of selection in the next breeding cycles, suggesting that GS approach is a promising strategy to accelerate breeding for heat tolerance in tomato. Finally, the annotation of SNPs located in gene body regions combined with QTL analysis allowed the identification of five candidates putatively involved in high temperatures response, and the building up of a GS model based on calibrated panel of SNP markers.

## Introduction

Tomato *(Solanum lycopersicum)* is one of the most important worldwide horticulture crops^1^. However, this crop face with high production losses caused by heat stress^2,3^, which heavily impact tomato fruit setting and yield related traits^4,5^. Indeed, temperature exceeding 30 °C during the day and 21 °C during the night can be very challenging for crop production^6^. Hence, heat stress is considered as a bottleneck factor for tomato fruit production, especially in temperate regions. Nowadays, developing tomato cultivars with improved yield under high temperature conditions may be a valuable strategy to cope with climate changes. The traditional plant breeding method for heat tolerance is based on the selection of advanced breeding lines showing greater yield performances than the current cultivars in a hot target production area. The plant evaluation is based on assessment of different quantitative and qualitative parameters such as pollen viability, fruit set, yield productivity per plant, total number of fruits, fruit shape, and soluble solid content^7^.

High-temperature tolerance is controlled by multiple genes which induce several physiological and biochemical changes, making the heat stress response hard to investigate^8^. For these reasons, conventional breeding approaches resulted inadequate to meet the growing demand for heat tolerant (HT) varieties, since time-consuming phenotypic evaluation rounds and complex selection schemes are needed^9^. In this regard, understanding the molecular mechanisms regulating the relationships among agronomic important traits can rapidly increase the selection of new genotypes.

In the past 10 years, significant progress has been made in precision breeding and in selection methods for early release of crop varieties^10^. Innovative breeding strategies such as marker-assisted selection (MAS), high-throughput genotyping and phenotyping platforms, reverse breeding, and genomic selection, are increasingly being used to complement the conventional approaches to bring the release of crop varieties to an important step forward^11–14^.

Tomato is a relative short life cycle crop and a high self-fertility species with a well know genome structure, offering the possibility to design more tailored crop improvement approach^15^. Several QTLs for abiotic environmental stresses (such as salinity, drought, and heat) and for fruit-related characteristics have been reported in this species^16–20^. However, QTLs related to complex traits are hard to introgress in cultivated crops, also by using MAS. This is especially due to the large number of small effect genes controlling heat tolerance^21,22^. Genomic selection (GS) is providing new opportunities for increasing the efficiency of plant breeding programs by overcome limiting factors^23–26^. The approach is based on estimating breeding values of an individual using genomics and phenotypic information. A training population (TRN) is evaluated at genotypic and phenotypic level to obtain a training set (TRS) and a test set (TST) used only for validation (i.e., evaluation of the accuracy). The trained GS model will be then applied to predict breeding values of individuals in the next selection step^27^. The basic idea of a GS approach is to use genome-wide marker data to predict genetic architecture of the TRN to effectively select the best individuals with superior traits. Thus, GS results in a robust approach to enhance the rate of genetic gain *per* unit of time and reduce the time required for screening superior individuals in breeding programs^14^. Successfully examples of GS approaches have been reported in several crops^26,28–30^, including tomato^31–34^. In addition, empirical studies have also demonstrated that GS has higher genetic gain than MAS for complex traits controlled by large number of QTLs^35^. However, although genotyping cost have decreased significantly in recent years, it is still a very onerous phase to carry out that slow down the overall GS-based breeding process. In theory, the GS prediction accuracy improves with higher genetic markers density, but several empirical studies showed that in population with long haplotypes overlap among individuals, accuracy seems to be relatively stable when a well calibrate set of markers is chosen^36–41^.

At light of these observations and to better investigate the basis of tomato heat stress tolerance, a F4 segregating population was phenotypically evaluated for quantitative and qualitative traits under heat stress condition. Then, a genotyping by sequencing (GBS) approach has been employed to produce de novo molecular markers useful to build up a suitable GS models for accelerating the selection of heat tolerant individuals. Several critical parameters such as size of training population, number and quality of markers were carefully evaluated. Subsequently, trained models were applied to the next generations to identify superior genotypes and a calibrated SNP subset was selected and tested to build up a model prediction. Finally, the annotation of SNPs located in gene regions was integrated with a QTL analyses to identify genes putatively involved in heat stress tolerance.

## Results

### Analysis of phenotypic traits in tomato segregating population for heat stress tolerance

In this study, a F4 tomato population deriving from single seed selection schema performed on the heat tolerant tomato variety JAG8810, was grown under high temperatures. Tomato plants were transplanted in June in the open fields under plastic tunnel with a delay of one month compared to the usual transplanting period (April) of the area to impose spontaneously occurring heat stressing conditions. As reported in Supplementary Fig. 1, the temperature was mostly upper than 30 °C during the day and exceeded the critical threshold level of 35 °C in 11 out of 81 days. Under these conditions, the JAGF4 lines were phenotyped for 8 traits, mainly related to fruit production and fruit quality. A wide range of variability in the eight production-related traits was observed and reported in Table 1 and Table S1.

**Table 1:** Mean, minimum and maximum values of traits related to fruit set, total fruit number per plant, yield per plant, and soluble solid content

Trait	Mean	Min	Max
FS (%)	38.9	8	78
TFN	158.2	32	354
YP (Kg/plant)	8.0	0.8	16
SSC	4.4	3	6.8

*FS (%)* fruit set percentage, *TFN* total fruit number per plant, *YP* yield production per plant, *SSC* soluble solid content

**Fig. 1:**
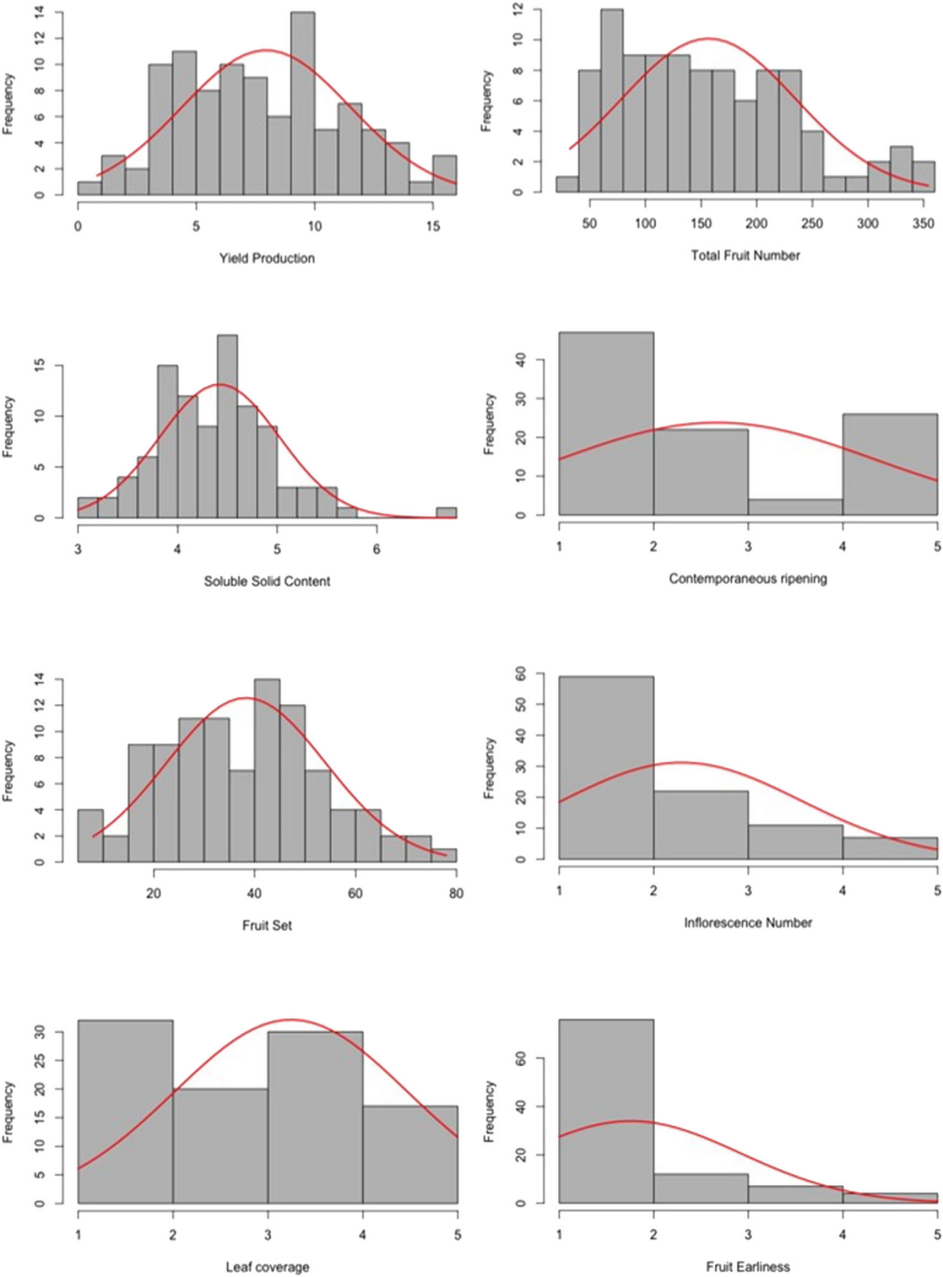
Frequency distribution of the phenotypic value of eight traits of interest. X-axis show phenotypic values whereas in the Y-axis the frequency is reported

Distributions without any significant skewness were observed for all analyzed traits except for inflorescence number and fruit earliness (Fig. 1).

**Fig. 2:**
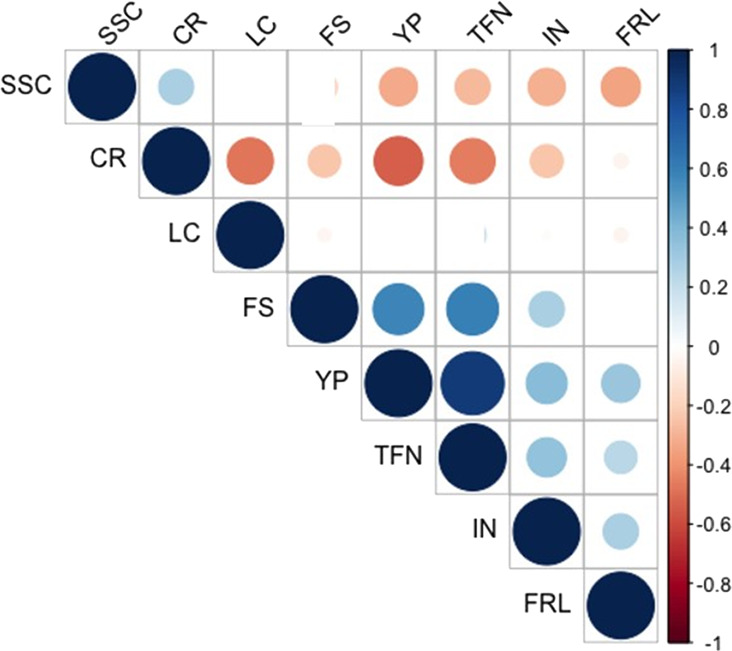
Pearson’s rank correlation coefficients between pairs of traits. Only correlation coefficients with *P* value < 0.05 after Bonferroni correction are shown. YP: yield production per plant; TFN: total fruit number per plant; SSC: Soluble solid content; CR: contemporaneous ripening; FS: percentage of fruit set; IN: inflorescence number; LC: leaf coverage; FRL: fruit earliness

Interestingly, significant correlations among parameters related to fruit production were found (Fig. 2). In particular, Pearson’s correlation coefficient (*r*) ranged from the lowest correlation (*r* = −0.53) between yield production per plant (YP) and contemporaneous ripening (CR) to the highest correlation (*r* = 0.89) between YP and total fruit number per plant (TFN) (Table S2). YP also significantly correlated with percentage of fruit set (FS) (*r* = 0.58), inflorescence number (IN) (*r* = 0.38) and fruit earliness (FRL) (*r* = 0.33) but negative correlated with soluble solid content (SSC) (*r* = −0.33). SSC negatively correlated with other yield-related traits such as TFN (*r* = −0.28), IN (*r* = −0.30) and FRL (*r* = −0.35). However, we found a weak positive correlation between SSC and CR (*r* = 0.27), which instead negative correlated with leaf coverage (LC) (*r* = −0.46).

### High-throughput genotyping of JAGF4 and JAGF5 populations

The hybrid variety JAG8810, 100 JAGF4, and 54 JAGF5 single individuals were genotyped by GBS approach producing a total of 117,798,988 and 136,926,433 reads in JAGF4 and JAGF5 populations respectively. After quality check, all reads were mapped onto the reference genome (*Solanum lycopersicum* cv. Heinz 1706 v3.0), and the results were used for SNP and INDELS discovery. Overall, 135,255 SNPs in JAGF4 and 176,596 SNPs in JAGF5 populations were found (Table 2).

**Table 2:** SNP distribution along tomato chromosomes in JAGF4 and JAGF5 populations

Chromosome	Length (bp)	SNP number JAGF4	SNP number JAGF5	% SNP JAGF4	% SNP JAGF5	H JAGF4	PIC JAGF4	H JAGF5	PIC JAGF5
Chr0	20852292	47913	47753	35.42	27.04	0.316	0.249	0.292	0.229
Chr1	98455869	5794	7238	4.28	4.1	0.252	0.199	0.21	0.166
Chr2	5597758	4119	4922	3.04	2.79	0.264	0.206	0.223	0.174
Chr3	72290146	4596	5882	3.4	3.33	0.269	0.211	0.202	0.158
Chr4	66557038	9892	12359	7.31	7	0.396	0.302	0.361	0.276
Chr5	66723567	13763	17481	10.18	9.9	0.410	0.313	0.369	0.28
Chr6	49794276	22500	27373	16.64	15.5	0.343	0.269	0.332	0.258
Chr7	68175699	2692	5142	2	2.91	0.188	0.149	0.113	0.09
Chr8	65987440	3676	4533	2.72	2.57	0.236	0.186	0.214	0.168
Chr9	72906345	2519	19947	1.86	11.29	0.171	0.136	0.028	0.022
Chr10	65633393	2776	3956	2.05	2.24	0.193	0.153	0.158	0.126
Chr11	56597135	10946	13278	8.09	7.52	0.339	0.262	0.305	0.238
Chr12	68126176	4069	6732	3.01	3.81	0.257	0.200	0.15	0.117

The chromosome length, the percentage of SNP per chromosome, the heterozygous values (H) and the polymorphism information content (PIC) are also reported

**Fig. 3:**
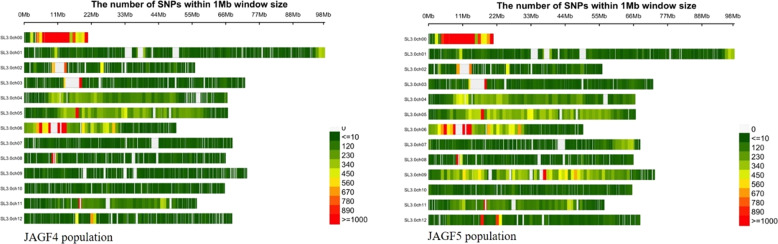
Distribution of SNPs density on the 13 chromosomes of tomato in JAGF4 and JAGF5 population. SL3.0Ch00 ~ SL3.0Ch12 in vertical axis are the serial number of 13 chromosomes. The horizontal axis shows chromosome length (Mb); 0 ~ > 1000 = depicts SNP density (the number of SNPs per window)

Their distribution along chromosomes is displayed in Fig. 3 showing a similar SNP density between two populations except for chromosome 9 where a higher SNP density was evident in JAGF5 compared with JAGF4 population. SNP number *per* chromosome ranged from 2519 (chromosome 9) to 22,500 (chromosome 6) with an average of 7278 in JAGF4 population. Instead, SNP number *per* chromosome varied from 3956 (chromosome 10) to 27,373 (chromosome 6) with an average of 10,736 in JAGF5 (Table 2). In JAGF4 the minimum average polymorphism information content (PIC) and heterozygous values (H) were encountered on chromosome 9 (0.136 and 0.171, respectively), while the maximum values were found on chromosome 5 (0.313 and 0.410, respectively). Similarly, in JAGF5 the minimum values were encountered on chromosome 9 (0.022 and 0.028 respectively) whereas the maximum PIC and H values were found on chromosome 5 (0.28 and 0.369, respectively).

**Fig. 4:**
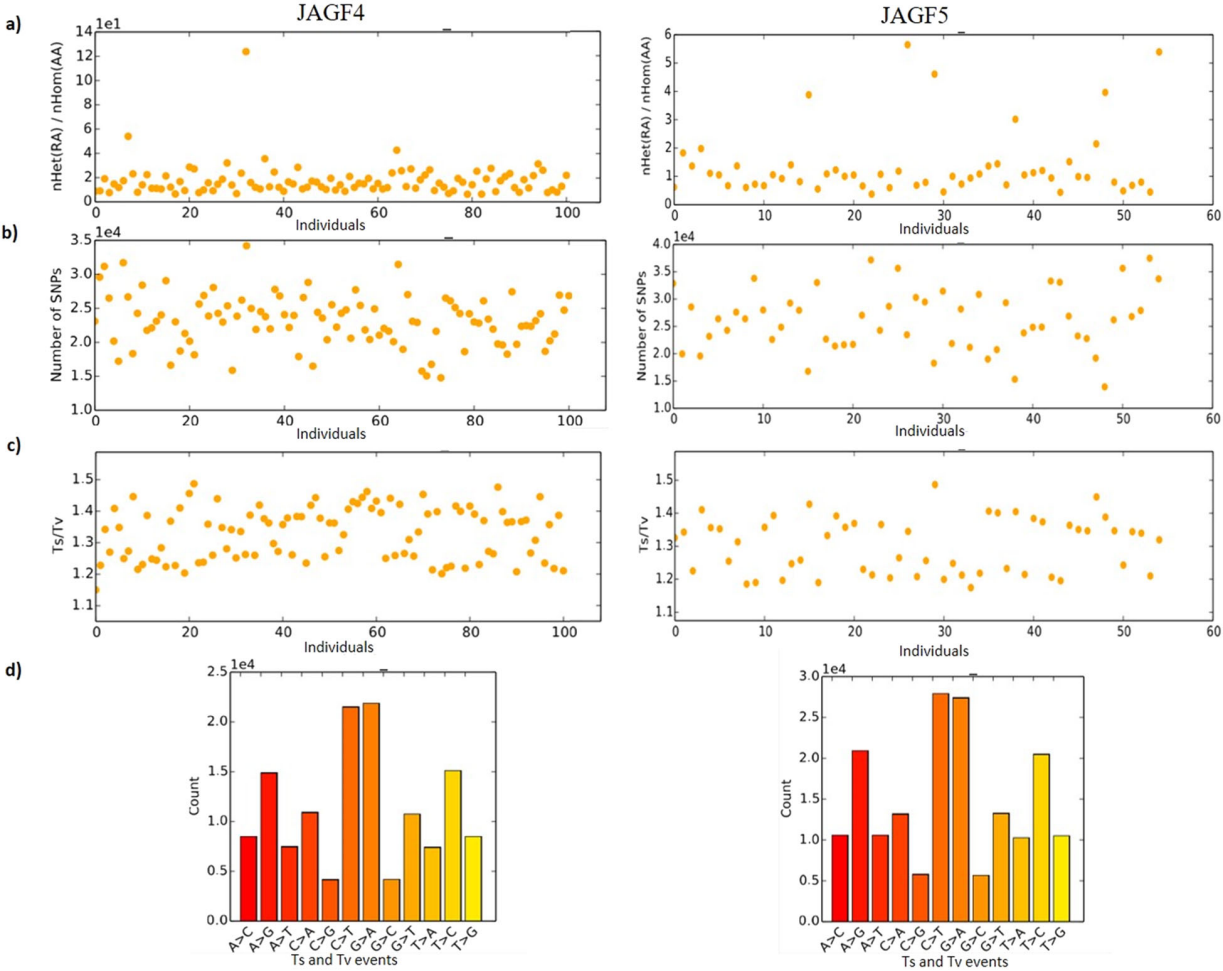
Nucleotide variants analysis. The heterozygous/homozygous number ratio (**a**), the SNP numbers (**b**), the observed transitions/transversions (Ts/Tv) ratios (**c**) per sample in JAGF4 and JAGF5 populations were showed. In **d** the specific Ts and Tv events were also displayed

The heterozygous/homozygous number ratio and the SNP number *per* sample were also displayed in Fig. 4a and Fig. 4b showing similar trends in the two populations. Also, the observed transitions/transversions (Ts/Tv) ratios in two studied populations were similar (Fig. 4c). In particular, we found a transitions/transversions (Ts/Tv) ratio of 1.19 in JAGF4 and 1.21 in JAGF5 with a total of 73,392 transitions and 61,863 transversions events in JAGF4 and 96,710 transitions and 79,886 transversions events in JAGF5 (Table S3). Among transitions events, C>T and G>A were the most abundant, whereas C>A and G>T were the most frequently transversion events in both populations (Fig. 4d). After removing, SNPs with minor allele frequency (MAF) less than 5%, a total of 101,797 and 109,967 SNPs in JAGF4 and JAGF5 population, respectively, were obtained.

### GS model training using the JAGF4 population

The JAGF4 genotypic and phenotypic data were used to train a prediction model for identifying the most suitable candidates for next-generation selection round (GS train). Given the positive correlations observed among the yield-related traits and the negative correlation between yield and the soluble solid content (SSC), we investigated the optimal design of GS models for YP and SSC traits using a TRN population of 90 and 100 individuals, respectively. As for the training of the GS models, the interaction between different sets of TRN composition and marker dataset were examined using a 5 × 5 contingency table. First, SNP dataset was filtered for five levels of percentage of eliminated missing values (PEMV) (70, 75, 80, 85, 90%) obtaining five different marker datasets for each trait (Table S4). Five TRN subsets of different composition were then obtained for each trait varying the TRS\TST ratio. TRS datasets ranging from 60 to 80 and 55 to 75 sampled genotypes for SSC and YP, respectively, were obtained randomly removing 5 genotypes to each round.

**Fig. 5:**
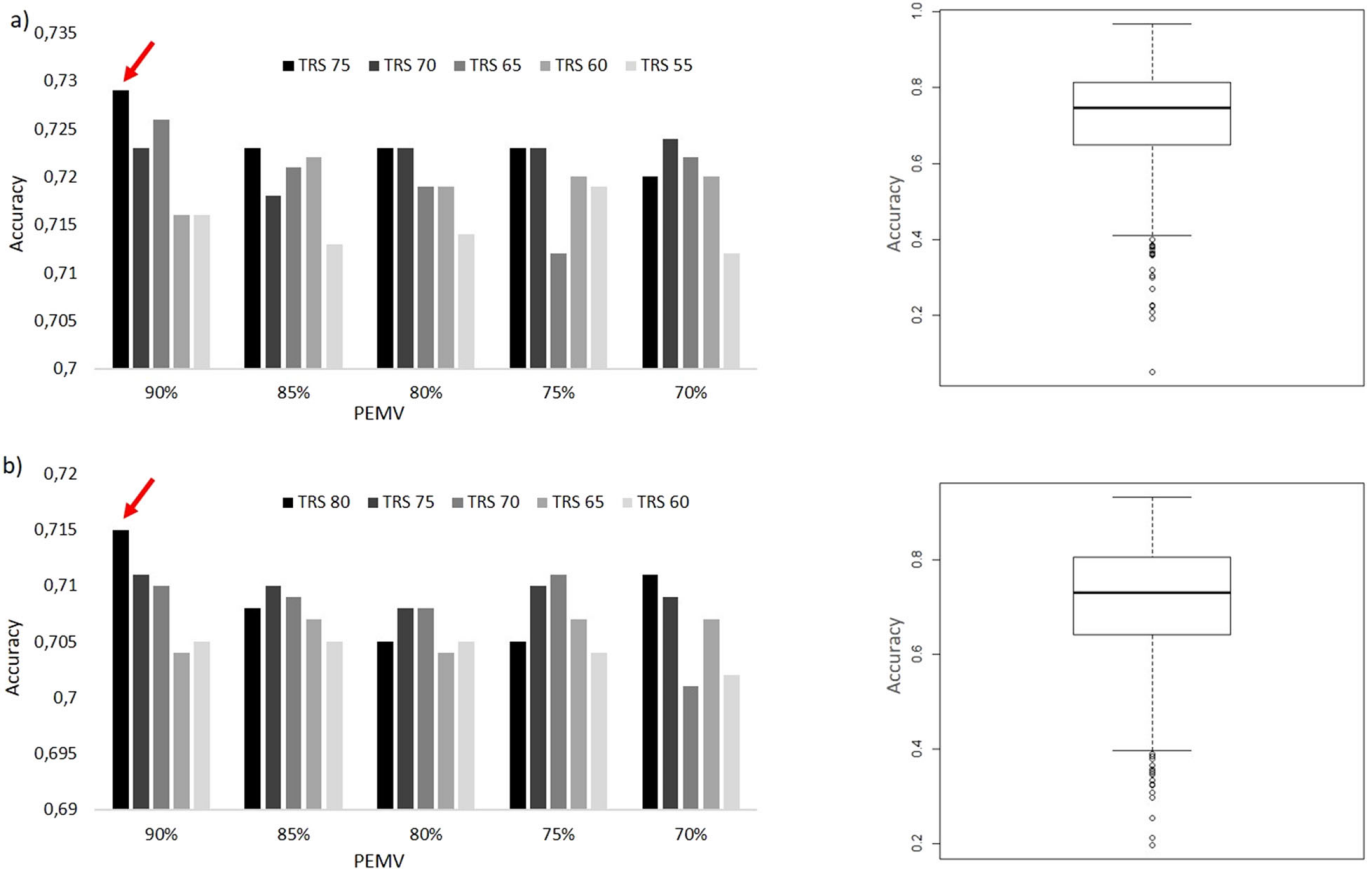
On the left prediction accuracies under different proportion of TRS and PEMVs. The red arrow indicates the best combination for YP (**a**) and SSC (**b**). On the right, the plot between correlation values and 1000 cycles of iteration was displayed for the best combinations

In this way, twenty-five datasets using 5 TRS\TST subsets of different composition and 5 PEMV filtered marker sets were assessed for each trait. To evaluate the prediction ability of RR-blup models to estimate the genetic breeding values (GEBVs), 1000 iteration cycles were run. The results revealed that predictive ability did steadily increase up until the maximum PEMV and larger TRS in the hold-out validation strategy for both traits. Indeed, prediction accuracy was maximum for the following combinations: PEMV 90% with a SNPs dataset of 14,286 and TRS\TST composition in 80\20 ratio for SSC and PEMV 90% with a SNP dataset of 14,210 and TRS\TST composition in 75\15 ratio for YP (Fig. 5). Genomic prediction accuracy in our trained models, defined here as the correlation between GEBV and phenotype value in the TST, were 0.729 for YP and 0.715 for SSC, respectively. The correlation values between GEBVs and real phenotypic values for each of the 1000 iteration cycles were also displayed in Fig. 5.

### GS model application in following generations to identify elite lines

The trained models build up in JAGF4 population were applied in next selection round to estimate GEBVs of F5 progeny population (JAGF5). After JAGF5 SNP dataset analysis, 10,648 common SNPs between the two populations were selected among high-quality SNPs and used for predicting GEBVs in JAGF5 and JAG8810 hybrid. In particular, the GEBVs of JAGF5 not phenotyped individuals were calculated by using the GS models trained on the JAGF4 individuals. Results obtained in this selection round are summarized in Table 3. To select the best and worst predicted performers, the JAG8810 GEBVs (7.9 and 4.6 for YP and SSC respectively) were used as threshold. Individuals with GEBVs higher than JAG8810 were predicted to be the best JAGF5 performers within the studied population. In the last step, the F6 offspring deriving from F5 plants and JAG8810 were phenotyped under heat stress condition to validate the goodness of prediction at phenotypic level for both analyzed traits (YP and SSC) and the best performers were selected among plants expressing phenotypic values higher than JAG8810 (Table 3).

**Table 3:** Summary of prediction and validation values in JAGF5 and JAGF6 populations

	YP GEBVs JAGF5	SSC GEBVs JAGF5	YP values JAGF6	SSC values JAGF6
JAG8810	7.9	4.6	3.5	4.3
% Best	57.4	42.6	16.7	70.4
% Worst	42.6	57.4	83.3	29.6

GEBVs and phenotypic values of JAG8810 were reported for YP and SSC. % of the best and worst values of predicted GEBVs in JAGF5 and phenotypic values in JAGF6 population were also reported

Correlations between the GEBVs calculated on F5 genomic data and the phenotypic values obtained in F6 offspring were 0.67 and 0.68 for YP and SSC, respectively. Once the model accuracy was validated also in the next selection round, we identified 8 best performers for YP and 23 for SSC which were predicted among the JAGF5 population and confirmed in the phenotyped JAGF6 (Table 4).

**Table 4:** Best JAGF5 predicted lines and JAGF6 best performers for YP and SSC were reported

	YP		SSC	
Lines	Best JAGF5	Best JAGF6	Best JAGF5	Best JAGF6
S2	✓	✓	✗	✓
S5	✓	✗	✗	✓
S20	✗	✗	✓	✓
S22	✓	✓	✗	✓
S30	✓	✓	✗	✗
S31	✓	✗	✓	✓
S33	✗	✗	✓	✓
S34	✓	✗	✗	✓
S36	✗	✗	✓	✓
S39	✗	✗	✓	✓
S41	✓	✓	✓	✗
S60	✓	✗	✗	✓
S62	✗	✗	✗	✓
S67	✓	✗	✓	✓
S68	✗	✗	✗	✓
S75	✓	✗	✗	✗
S76	✗	✗	✓	✓
S77_1	✓	✗	✗	✗
S77_2	✓	✗	✗	✗
S91	✓	✗	✗	✗
S92	✓	✗	✗	✗
S96	✗	✗	✗	✗
S97	✗	✗	✓	✓
S98	✓	✗	✗	✗
S99	✗	✗	✓	✓
S104	✓	✗	✗	✗
S105	✓	✗	✗	✓
S112	✗	✗	✓	✓
S117	✗	✗	✓	✓
S118	✓	✗	✗	✗
S121	✗	✗	✓	✓
S126	✗	✗	✓	✓
S127	✗	✗	✓	✓
S128	✗	✗	✓	✓
S130	✗	✗	✓	✓
S132_1	✓	✗	✗	✗
S132_2	✓	✗	✗	✓
S139	✗	✗	✓	✓
S141	✓	✗	✗	✗
S151	✗	✗	✓	✓
S153	✓	✓	✗	✓
S159	✓	✗	✗	✓
S160	✗	✗	✓	✓
S179	✗	✗	✓	✓
S180	✓	✗	✗	✓
S185_1	✓	✗	✗	✗
S185_2	✓	✗	✗	✓
S194	✓	✗	✗	✗
S196	✓	✓	✗	✗
S205	✓	✓	✗	✓
S218	✗	✗	✓	✓
S225_1	✓	✓	✗	✓
S225_2	✓	✗	✗	✓
S239	✗	✗	✓	✓

✓indicate lines with GEBV and/or phenotypic values higher than JAG8810; ✗indicate lines with GEBV and/or phenotypic values lower than JAG8810

**Fig. 6:**
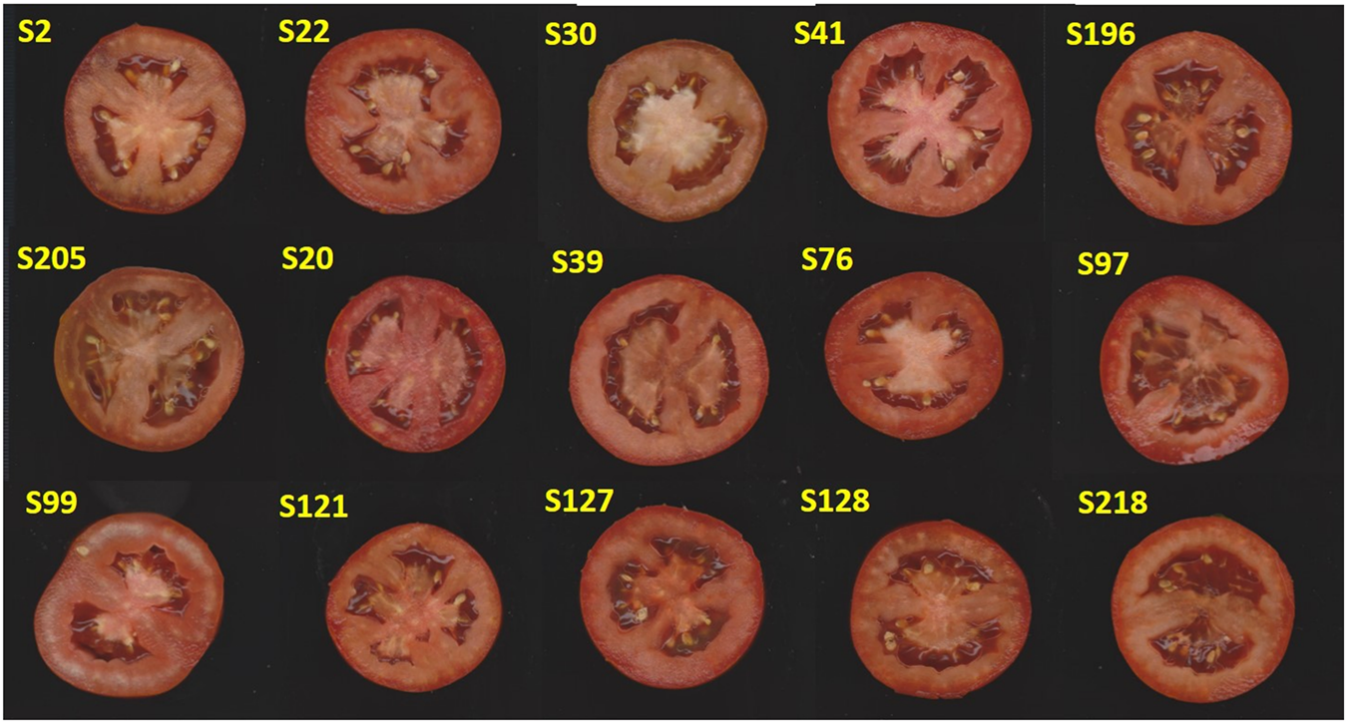
Fruit longitudinal section of selected elite lines. Fruit longitudinal sections were shown

Selected genotypes could be used as extremely promising elite lines in advanced breeding programs (Fig. 6).

### Variant effect prediction and QTL analysis to highligh loci involved in heat tolerance

**Fig. 7:**
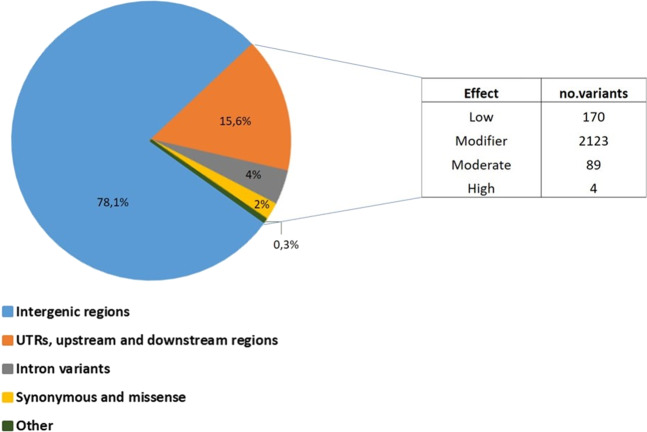
Annotation of 10.648 high-confidence SNPs using SnpEff showing the classification of SNPs based on effects. The effect of SNP located in genic regions was also reported

To identify novel alleles contributing to heat tolerance, we performed an integrative analysis on our SNP dataset. A genomic variant annotation (defined as assignment of variant function) of all 10,648 high confidence SNPs was conducted. As showed in Fig. 7, the largest proportion of the SNPs was categorized as intergenic regions (78.1%) whereas only 21.9% of SNPs were in gene body regions. In particular, variants located in UTRs, upstream and downstream regions accounted for 15.6%. A small proportion of SNPs were categorized as intron variants (4%), synonymous variants and missense variants (2%). Instead, the category “other” included splice acceptor variant, splice donor variant, splice region variants, and stop gained which accounted for only 0.3%. Within the derived SNPs subset of 2,278 SNPs in gene body regions, the variants with a modifier and low effect on the proteins were 2185 (accounted for 96%). Those with a moderate effect were 89 (3.9%) whereas 4 SNPs (0.1%) showed a disruptive or high impact effect on the corresponding proteins.

The 93 SNPs classified as having moderate and high impact were further analyzed in more detail. They targeted 52 genes for which the Solyc ID, the number of variants per gene, the predicted impact of the mutation, and the protein function are reported in Table 5. The 52 genes affected by these variations are distributed on all chromosomes except chromosome 7 and 9 and some of them could be involved in abiotic stress responses.

**Table 5:** Solyc ID, number of variants, SNP impact and function of the 93 selected variants identified were reported

Solyc ID	Number of SNP	SNP impact	Function
Solyc00g008040.1	3	Moderate	alpha/beta-Hydrolases superfamily protein
Solyc00g012704.1	10	Moderate	Pex2/Pex12 N-terminal domain-containing protein
Solyc01g013910.1	1	Moderate	Endoribonuclease Dicer 2b
Solyc01g107700.3	1	Moderate	Kynurenine formamidase
Solyc01g108047.1	4	Moderate	GATA transcription factor-like protein
Solyc01g108083.1	7	Moderate	Glucosamine--fructose-6-phosphateaminotransferase (Isomerizing)
Solyc01g108320.3	3	Moderate	Peroxidase
Solyc01g108460.2	1	Moderate	Serine carboxypeptidase 1
Solyc01g109150.4	2	Moderate	cytochrome P450 CYP74C4
Solyc01g109160.4	1	Moderate	cytochrome P450 CYP74C4
Solyc01g110820.3	1	Moderate	RNA-binding protein 40;
Solyc01g110995.1	1	Moderate	4-coumarate:CoA ligase 1
Solyc01g111240.3	3	Moderate	Translocase of chloroplast 90, chloroplastic
Solyc01g111430.2	2	Moderate	Pentatricopeptide repeat-containing protein
Solyc01g111620.3	1	Moderate	AT4G35080-like protein (Fragment); Nickel/cobalt transporter, high-affinity
Solyc01g111630.3	1	Moderate	D-3-phosphoglycerate dehydrogenase
Solyc01g111870.2	1	Moderate	Serine/threonine-protein kinase
Solyc01g111980.3	4	Moderate	Lysine/histidine transporter
Solyc01g112130.3	1	Moderate	Dimethylaniline monooxygenase 5
Solyc01g112330.1	1	Moderate	ARID/BRIGHT DNA-binding domain-containing protein
Solyc02g085970.1	1	Moderate	FACT complex subunit SSRP1
Solyc03g046596.1	1	Moderate	BCL-2-associated athanogene 6
Solyc03g070435.1	1	Moderate	Alpha-mannosidase
Solyc03g113310.1	1	Moderate	Pseudouridine synthase family protein
Solyc03g113890.1	1	Moderate	Zinc finger protein 6
Solyc03g114040.3	1	Moderate	Os04g0347800 protein (Fragment)
Solyc04g015130.3	2	Moderate	Ribosomal protein S6 kinase alpha-3
Solyc04g074880.3	1	Moderate	Purine permease family protein
Solyc04g080610.3	1	Moderate	Ornithine carbamoyltransferase
Solyc05g025530.1	1	Moderate	DNA-directed RNA polymerase subunit beta
Solyc05g025540.1	1	Moderate	Molybdenum cofactor sulfurase
Solyc06g006057.1	2	Moderate	Leucine-rich receptor-like protein kinase family protein
Solyc06g007310.3	1	Moderate	Deoxyribonuclease tatD
Solyc06g007530.2	1	Moderate	B3 domain-containing protein Os05g0481400
Solyc06g008720.3	1	Moderate	Zinc ion binding protein
Solyc06g009920.1	4	Moderate	ATPase E1-E2 type family protein / haloacid dehalogenase-like hydrolase family protein
Solyc06g036260.3	1	Moderate	Beta-carotene hydroxylase 1
Solyc06g036485.1	2	Moderate	Kinase family protein
Solyc06g051190.2	1	Moderate	RNA-dependent RNA polymerase family protein
Solyc08g062990.1	1	Moderate	Protein kinase superfamily protein
Solyc10g061840.2	2	Moderate	Carboxyl methyltransferase 4
Solyc10g044430.1	2	Moderate	FMN-linked oxidoreductases superfamily protein
Solyc11g028270.1	1	Moderate	Bidirectional sugar transporter SWEET
Solyc11g039917.1	2	Moderate	Retrovirus-related Pol polyprotein from transposon gypsy
Solyc11g039410.2	1	Moderate	NAD(P)H-quinone oxidoreductase subunit K, chloroplastic
Solyc11g056540.1	4	Moderate	Dynein-1-alpha heavy chain, flagellar inner arm I1 complex
Solyc11g061890.2	1	Moderate	Adenylate isopentenyltransferase
Solyc12g076355.1	1	Moderate	3-oxo-5-alpha-steroid 4-dehydrogenase family protein
Solyc00g013184.1	1	High	ubiquinone biosynthesis COQ9-like protein
Solyc05g026265.1	1	High	Retrovirus-related Pol polyprotein from transposon TNT 1-94
Solyc11g045677.1	1	High	vesicle-associated protein 1–4
Solyc11g045260.1	1	High	Photosystem II CP43 reaction center protein

To highlight putative important loci, a QTL analysis for YP based on JAGF4 population dataset was also performed (Supplementary Fig. 2). Three major QTLs on chromosome 3, 6, and 11 explaining 70, 86, and 53% of the phenotypic variance respectively were identified. Interestingly, the QTL region on chromosome 3 underlined Solyc03g046596.1 and Solyc03g070435.1 variant genes with moderate impact, coding for BCL-2-associated athanogene 6 and Alpha-mannosidase, respectively (Table 5). The large QTL chromosome region on chromosome 6 contained Solyc06g007530.2, Solyc06g008720.3, Solyc06g009920.1, Solyc06g036260.3, and Solyc06g036485.1 variant genes whereas on chromosome 11 QTL region Solyc11g028270.1, Solyc11g045677.1, and Solyc11g045260.1 variant genes, with moderate or high impact, coding for Bidirectional sugar transporter SWEET, vesicle-associated protein 1–4 and Photosystem II CP43 reaction center protein respectively, were found (Table 5). In addition, a QTL on chromosome 9 explaining the 51% of the phenotypic variance was identified. This QTL underlined 4 genes, Solyc09g042480.2, Solyc09g031970.3, Solyc09g031975.1, and Solyc09g031780.3, showing SNPs with a modifier impact and coding for chromatin remodeling complex subunit, glycogen/starch/alpha-glucan phosphorylase, 60S acidic ribosomal protein family and chloroplast inner envelope family protein respectively.

Finally, we re-trained the GS models on the JAGF4 population retrieving only SNPs located in gene body regions and this resulted in a new optimized dataset of 2278 SNPs. To evaluate the prediction ability of the revised RR-blup models, 1000 cycles of iteration were run, using the optimized parameters previously described. Limiting the SNP dataset to a subset of 2278 SNPs in gene body regions did not significantly affected the accuracies of trained GS models if compared with previous models trained using the overall SNPs dataset (0.721 vs 0.729 for YP). By contrast a slight worsen accuracy (0.701) was observed using a dataset of 2278 SNPs randomly chosen from the starting dataset of 10,648 SNPs.

## Discussion

Currently, tomato breeding is worldwide prospectively adapting conventional goals to the predicted scenario of changing environmental conditions as consequence of the global warming. In this view, the development of new high-yielding varieties tolerant to challenging high-temperature events appear to be of increasing interest. In this paper, we propose for the first time a GS approach based on a Single Seed Descendent (SSD) schema implemented in a population deriving from a heat tolerant tomato variety. The obtained F4 segregating progenies were screened for agronomic performance under heat stress conditions and genotyped to build up a GS model. Phenotypic evaluation revealed that traits related to fruit production such as FS, YP, and TFN, which are usually highly affected by high temperatures in tomatoes^42,43^, showed a wide variation. Yield positively correlated with other production-related traits such as TFN, FS, IN, FRL, confirming that they are primary traits involved in final yield performance under heat tolerance^5,44–46^. By contrast, yield-related traits resulted negatively correlated to SSC and CR indicating that increase in yield attributes results in decrease in quality components. Fruit ripening is a complex process involving numerous physiological and biochemical changes and uneven ripening probably reduced yield being out from sugar upsetting the source/sink relationships. It is widely reported that tomato varieties with high SSC tend to be less productive^47^, although it represents an important tomato selection parameter. The number of diverse factors involved in yield under heat stress conditions clearly suggested that, overall, the trait is under complex genetic control and addressing at the same time most of that factors would increase the chance to have success in term of selection gain.

Although GS has been previously applied for key productive traits such as YP and SSC in tomato^31–33^, there are no reports on GS implementation under heat stress conditions to our knowledge. Recent studies have demonstrated that the establishment of GS experiment optimal parameters such as TRN size, TRS and TST relatedness, marker density, precision of the phenotyping is relevant for a reliable prediction^48–53^. Training populations with strong relationship to the training set ensure higher prediction accuracies^50^. TRN design where individuals from the same family are used as both the TRN and TST has been extensively used in several breeding programs^54^ such as in wheat^55–57^, maize^58^, rye^59^. Balancing LD present in initial segregating populations^60,61^ can help to obtain higher prediction accuracy also with a relatively small population size. Therefore, we trained our model in F4 segregating generation for both maintaining TRN–TST relationship and capturing long chromosome stretches in linkage disequilibrium (LD). We obtained a good prediction values (0.729 for YP and 0.715 for SSC) besides the limited population size (100 lines), highlighting the significance of full training-panel design. However, this type of design has some disadvantages since prediction models developed from single biparental populations have limited applications outside of particular selection scheme. Therefore, TRN designs that combine data from both related and unrelated families would be useful for plant breeders. In this case, the TRN must be created by using progenies of different genetic backgrounds, including full sibs, half-sibs, and other individuals with related ancestry. Of course, this TRN design has the great advantage of adapting well to implement GS in a wide range of breeding programs, but several studies have demonstrated that the prediction accuracy of GS substantially reduces especially when TRNs consisting of only unrelated individuals to the TST^62–65^^.^

At the same time our TRN was tested in combination with other parameters to improve the precision of model developed in JAGF4. In particular, genotyping-by-sequencing (GBS) detected high-density markers with similar chromosome distribution as well as the heterozygous/homozygous ratio in both studied populations. The effects of Training Set size and GBS marker subsets filtering (by MAF and PEMV) on the model accuracy were evaluated in JAGF4 population to train the GS models. Starting from the complete dataset of 135,255 SNPs it was first filtered by MAF obtaining a dataset of 101,797 SNPs. Then our dataset was gradually reduced as PEMV increase, revealing that using a reduced subset of markers of ~14,000 filtered by PEMV of 90% did not impacted negatively on the overall prediction accuracy. This is an important point to take in account, since the use of fewer markers would result in significant cost reductions for genotyping but would also impact the extent of LD that is picked up by the prediction models. Moreover, our result clearly indicated that the marker dataset composed from the 10,648 high quality SNPs shared between the two generations (JAGF4 and JAGF5) was able to accurately track the genome of plants along the generations in order to successfully select individuals with the highest GEBV in JAGF5 and JAGF6. Our findings are in agreement with other studies that have shown that a reduced numbers of SNPs can be used in following stage of GS selection^39–41,66,67^. Indeed, our approach allowed the development of a GS model and its validation in field experimental trials reducing the number of generations and the size of the evaluated population. The GS models for YP and SSC obtained in this study efficiently predicted potential elite lines in F5 generation and accelerate selection for heat tolerant genotypes.

In addition, the GS performed on a subset of 2,278 variants mapped in gene body regions, suggests that the marker location is an important component of predictive ability of a genomic model since the accuracy was similar to the one obtained with the full dataset. Most of the 2,278 identified variants were located in UTRs, upstream and downstream regions that might highly affect transcription and/or translation and therefore might be useful for future applications^68^. Moreover, the prediction of SNPs impact and the QTL analysis allowed the identification of four genes with high or moderate impact variants (Solyc03g046596, Solyc06g036260, Solyc11g045677, and Solyc11g028270), involved in thermotolerance and regulation of floral development and fruit set. The BAG (Bcl-2-associated-athanogene) Solyc03g046596 protein is a nucleotide exchange factors^69^, involved both in programmed cell death^70,71^ and in basal thermotolerance^72^. BAG6 is also involved in the heat stress signal transduction pathway^73,74^ and in the re-folding of heat-denatured proteins^75^. The Beta-carotene hydroxylase Solyc06g036260 gene, involved in the xanthophyll cycle (the reversible interconversion of two carotenoids, violaxanthin, and zeaxanthin), has a key role in photoprotection^76,77^ and therefore it represents a promising target for genetic engineering to enhance heat stress tolerance^78,79^. Two interesting genes, located on chromosome 11, were also found. The Solyc11g045677, showing a SNP with high impact, encodes the vesicle-associated protein 1–4, important for maintaining homeostasis, cell growth and development, and polarity^80^ during the regulation of abiotic stress response^81–83^. The Solyc11g028270, with a moderate impact variant, encodes for Bidirectional sugar transporter SWEET, involved in the regulation of plant growth and development, a key traits for manipulating the carbohydrate partitioning process^84–86^ to improve abiotic stress tolerance and yield. Moreover, on chromosome 9 QTL region is located the Solyc09g042480 gene, involved in chromatin remodeling complex (CHR) synthesis, that display a SNP with a modifier impact. It is widely reported that CHRs are implicated in the plant response to heat stress^87,88^. Therefore, genetic variability highlighted into the 5 tomato loci could account for altered control of critical processes leading to heat tolerance.

## Conclusions

In the present study, different strategies were integrated to promote both the identification of traits involved in high temperature response and the selection of superior genotypes tolerant to heat stress in tomato. Optimized genomic prediction models for yield production and soluble solid content were developed, showing that GS is a valuable strategy to accelerate breeding for heat tolerance in tomato. In addition, the prediction of variant impact performed on our marker dataset integrated with the QTL analysis allowed us to select putative loci involved in high temperatures response to be further investigate. The GS model build up with the subset of SNPs located in gene body regions showed an accuracy similar to the one obtained with the full dataset, suggesting that the marker location is an important component of predictive ability of our model.

## Materials and methods

### Field trials and phenotypic evaluation of segregating populations

The tomato variety JAG8810, kindly obtained from Bayer station (Latina, Italy) was self-pollinated from F2 to F4 generations through a Single Seed Descendent (SSD) schema (Supplementary Fig. 3) in greenhouse of the Department of Agriculture of the University of Naples Federico II (N 40° 48.′ 49.352′′; E 14° 20′ 40.073′′). In 2017, 100 F4 lines (JAGF4) were grown in open field in Southern Italy region (Battipaglia-Campania; N 40° 58.′ 56.69′′; E 14° 96′ 10.02”), usually characterized by high temperatures during the flowering and fruit set periods (from June to August). Five seedlings for each JAGF4 line were transplanted in field under plastic tunnel in a completely randomized design in the beginning of May 2017 in order to expose plants to high temperatures. Tomato plants were grown following the standard cultural practices of the area and temperature and climatic data were recorded using the weather station VantagePro2 (Davis Instrument Corporation).

Three random plants per genotype were analyzed for traits related to flowering, fruit production and quality. For four of these traits (fruit earliness (FRL), leaf coverage (LC), inflorescences number (IN) and contemporaneous ripening (CR) a score (from one to five) was calculated. The percentage of fruit set (FS) were evaluated on inflorescences produced from the second to the fifth truss on three plants per line randomly chosen. Finally, at fruit red ripe stage, total fruit number (TFN), soluble solid content (SSC-3 fruits per plant) and yield production per plant (YP) were measured. Correlation analyses was carried out using R package “corrplot”^89^. Fifty-four JAGF6 offspring plants deriving from the JAGF5 population and the hybrid JAG8810 were grown and phenotyped in 2019 as described for JAGF4 population (Battipaglia-Campania; N 40° 58.′ 56.69′′; E 14° 96′ 10.02′′) except for the planting in double rows. At fruit red ripe stage, the Soluble Solid Content (SSC-3 fruits per plant) and yield production per plant (YP) were measured in order to validate the model.

### DNA extraction and sequencing

DNA was extracted from 100 mg of young leaf tissues from JAG8810, 100 JAGF4, and 54 JAGF5 single individuals, using the Qiagen DNeasy Plant kit (Qiagen, Hilden, Germany). The DNA concentration was estimated using the Qubit fluorometer (Invitrogen, Carlsbad, CA, USA) and the 260/280 and 260/230 ratios were assayed using the NanoDrop 1000 spectrophotometer (Thermo Fischer, Waltham, MA, USA). DNA samples were sent to the IGA technology services (Udine, IT) for genotyping by sequencing (GBS) approach. In silico analysis on the reference genome was used to select the best combination of the two restriction enzymes *Sph I* and *Mbo I* and the best fragment size distribution to obtain the desired number of loci. Libraries were processed with Illumina cBot for cluster generation on the flowcell, following the manufacturer’s instructions and sequenced with V4 chemistry paired end 125 bp mode on HiSeq2500 instrument (Illumina, San Diego, CA).

### Read mapping, variant calling, and annotation

A quality check was performed on the raw sequencing data to remove low quality portions while preserving the longest high quality part of NGS reads. The minimum length was set to 35 bp and the quality score to 25. The software BBDuk was used for this scope.

The high-quality reads were aligned against the *Solanum lycopersicum* reference genome sequence (SL3.0) with BWA aligner^90^. Default parameters and selection of uniquely aligned reads (i.e., reads with a mapping quality >4) were used. Filtering of detected loci was carried out using the Populations program included in Stacks v2.0^91^. Populations was run with option –*r* = 0.75 in order to retain only loci that are represented in at least the 75% of the population. Within the Stacks v2.0 package, the “ref_map.pl” program was used for variant calling^90^. This program aligned data to the reference genome. A VCF file was created and filtered by Minimum Read Depth (DP) > = 4. Variant Calling Filter Files (VCFs) containing all the identified variants were obtained. The VCF file were subjected to a filtering procedure using Vcftools v.0.1.13 (http://vcftools.sourceforge.net/) setting the percentage of eliminated missing values at different levels and the minor allele frequency at 0.05.

SNP data in VCF format are publicly available at FIGSHARE with the following link (https://figshare.com/s/9c64512c04c5bc220e22). Users can download and use the data for research purpose with acknowledgment to authors. In order to perform a variant annotation of the Variant Calling Files (VCF), the SnpEff^92^ was employed on JAGF4 population dataset, which let the association of each variant to the annotated genes and to predict their effect on the protein function. Variants were classified based on their location (e.g., exon, intron, intergenic regions, splice sites, etc.) and the effect of their ‘impact’*:* High, Moderate, Low and Modifier.

### Genomic selection model construction and optimization

The 100 phenotyped and genotyped JAGF4 individuals were used to build up a TRN. The TRN was divided in TRS e TST in a hold-out validation scheme, meaning that the model was build using X individuals of the TRS and validated in the TST with remaining 100-X lines. Genomic breeding values (GEBVs) for YP and SSC were estimated using ridge regression best linear unbiased prediction^93^.

The predictive ability of genomic selection models was evaluated calculating the Pearson correlation between the phenotypic values and the GEBVs (predicted) values across 1000 cycles of iteration.

The hold-out validation was implemented with five different samples sizes in test set (15, 20, 25, 30, and 35 for YP and 20, 25, 30, 35, 40 for SSC). For each sample size, five different set of SNPs filtered by minor-allele frequency (MAF) > 0.05, based on different levels of percentage of eliminated missing values (PEMVs: 90, 85, 80, 75, 70%), were used. After filtering, remaining missing values were imputed using the EM method with the A.mat function of the “rrBLUP” package in R^93^. Therefore, a total 25 models with different sample sizes and number of SNPs were compared. For each of the 25 models, random sampling was repeated 1000 times. The best combination was selected to calculate the final accuracies of the trained models. After JAGF5 SNP dataset analysis, 10,648 common SNPs between the two populations were selected among high-quality SNPs (Table S5). This SNP dataset and the phenotypic values of the 100 F4 individuals were used as TRS to estimate GEBVs of F5 progeny population. A phenotypic characterization under heat stress conditions in F6 offspring deriving from F5 population was employed to validate the model.

### SNP dataset evaluation

Starting from the previously SNP dataset used for the JAGF5 prediction model, SNP located in intergenic region were removed and the predictive ability of genomic selection models was evaluated calculating the Pearson correlation between the phenotypic values and the GEBVs (predicted) across 1000 cycles of iteration in the original training population (F4).

### Quantitative trait locus (QTL) analysis

QTL analyses for YP was performed on JAGF4 population using R-based software package, R/qtl^94,95^ in R version 4.0.5. The dataset of 10,648 common SNPs between JAGF4 and JAGF5 individuals was used. Frist SNP markers were coded as AA, BB, AB, and NA respectively for major allele, minor allele, heterozygosity and missing data. Then, genetic maps were constructed by est.map using the Kosambi’s function of R-qtl for the conversion of recombination frequency to genetic distance^96^. Composite interval mapping (CIM) was applied for QTL detection using the maximum likelihood via the expectation-maximization algorithm^97^ with a window size (window) of 15 cM and the marker covariate (n.marcovar) equals to 4. Finally, significance thresholds were generated for each trait by using the permutation test (α = 0.05, *n* = 1000). The percentage phenotypic variance explained by a QTL was estimated via the following formula: h2 = 1–10 ^(−2* LOD/n)^, where “n” is the sample size and “LOD” the single-QTL model LOD score, using single interval mapping (SIM). The whole genome was scanned in steps of 1 cM by the scanone function. The 95% Bayes credible interval was assessed using the bayesint function, using the LOD score of CIM.

## Supplementary information

Table S5

Table S1

Table S2

Table S3

Table S4

Supplementary Figures

## Data Availability

Data obtained are publicly available at 10.6084/m9.figshare.14937795.
