# Supplementary material for: Tomato genomic prediction for good performance under high-temperature and identification of loci involved in thermotolerance response
Source: Hortic Res. 2021 Oct 1;8:212. doi: 10.1038/s41438-021-00647-3 (PMC8484564; doi:10.1038/s41438-021-00647-3)
Supplement: Supplementary file 1 — Table S5 [file 41438_2021_647_MOESM1_ESM.pdf]

Table S5. List of 10,648 highly informative SNPs common to the two populations (JAGF4 and JAGF5)

SNP Number	Chromosome	Genome position
1	Chr0	1127847
2	Chr0	2076463
3	Chr0	2076464
4	Chr0	2076854
5	Chr0	2076879
6	Chr0	2110256
7	Chr0	2124927
8	Chr0	2124942
9	Chr0	2129157
10	Chr0	3848596
11	Chr0	4103937
12	Chr0	4103922
13	Chr0	4103908
14	Chr0	4103898
15	Chr0	4103892
16	Chr0	4103886
17	Chr0	4103877
18	Chr0	4103857
19	Chr0	4234007
20	Chr0	4234009
21	Chr0	4234051
22	Chr0	4234054
23	Chr0	4234233
24	Chr0	4234263
25	Chr0	4234275
26	Chr0	4289462
27	Chr0	4289450
28	Chr0	4289155
29	Chr0	4294313
30	Chr0	4294258
31	Chr0	4294225
32	Chr0	4293991
33	Chr0	4293984
34	Chr0	4293980
35	Chr0	4293975
36	Chr0	4293900
37	Chr0	4301731
38	Chr0	4301732
39	Chr0	4301755
40	Chr0	4301756
41	Chr0	4301805
42	Chr0	4301812
43	Chr0	4301817
44	Chr0	4301824

45	Chr0	4302021
46	Chr0	4302057
47	Chr0	4302086
48	Chr0	4331234
49	Chr0	4331230
50	Chr0	4331215
51	Chr0	4331194
52	Chr0	4331188
53	Chr0	4331176
54	Chr0	4330963
55	Chr0	4330955
56	Chr0	4330906
57	Chr0	4582338
58	Chr0	4582358
59	Chr0	4582402
60	Chr0	4582413
61	Chr0	4582416
62	Chr0	4782674
63	Chr0	4797620
64	Chr0	4996408
65	Chr0	4996418
66	Chr0	4996421
67	Chr0	4996422
68	Chr0	4996444
69	Chr0	4996450
70	Chr0	4996458
71	Chr0	4996728
72	Chr0	4996760
73	Chr0	4996816
74	Chr0	4996830
75	Chr0	5104605
76	Chr0	5104577
77	Chr0	5104573
78	Chr0	5104558
79	Chr0	5104522
80	Chr0	5104229
81	Chr0	5104220
82	Chr0	5104217
83	Chr0	5104199
84	Chr0	5104197
85	Chr0	5104195
86	Chr0	5104174
87	Chr0	5104165
88	Chr0	5110118
89	Chr0	5110131
90	Chr0	5110157
91	Chr0	5110159

92	Chr0	5110162
93	Chr0	5110179
94	Chr0	5110187
95	Chr0	5110392
96	Chr0	5110409
97	Chr0	5110411
98	Chr0	5110412
99	Chr0	5110413
100	Chr0	5110414
101	Chr0	5110422
102	Chr0	5110424
103	Chr0	5110433
104	Chr0	5110437
105	Chr0	5110445
106	Chr0	5110450
107	Chr0	5110463
108	Chr0	5110464
109	Chr0	5845193
110	Chr0	5904146
111	Chr0	5930645
112	Chr0	5930644
113	Chr0	5935601
114	Chr0	5986861
115	Chr0	5986722
116	Chr0	5986366
117	Chr0	5991474
118	Chr0	6024464
119	Chr0	6024450
120	Chr0	6025141
121	Chr0	6025156
122	Chr0	6025344
123	Chr0	6034168
124	Chr0	6075375
125	Chr0	6075385
126	Chr0	6129726
127	Chr0	6129747
128	Chr0	6132800
129	Chr0	6132791
130	Chr0	6132752
131	Chr0	6132709
132	Chr0	6276530
133	Chr0	6288627
134	Chr0	6288984
135	Chr0	6288985
136	Chr0	6288990
137	Chr0	6300589
138	Chr0	6300635

139	Chr0	6300828
140	Chr0	6314736
141	Chr0	6314785
142	Chr0	6318892
143	Chr0	6318866
144	Chr0	6348587
145	Chr0	6354555
146	Chr0	6354524
147	Chr0	6383323
148	Chr0	6424145
149	Chr0	6496768
150	Chr0	6501011
151	Chr0	6857097
152	Chr0	6861697
153	Chr0	6864758
154	Chr0	6872163
155	Chr0	6911707
156	Chr0	6918292
157	Chr0	6921557
158	Chr0	6921585
159	Chr0	6923968
160	Chr0	6935634
161	Chr0	6935639
162	Chr0	6935667
163	Chr0	6935712
164	Chr0	6936025
165	Chr0	6936042
166	Chr0	6936047
167	Chr0	7004426
168	Chr0	7004413
169	Chr0	7004407
170	Chr0	7004394
171	Chr0	7004392
172	Chr0	7004373
173	Chr0	7004363
174	Chr0	7004357
175	Chr0	7004355
176	Chr0	7038933
177	Chr0	7041350
178	Chr0	7131165
179	Chr0	7153066
180	Chr0	7153344
181	Chr0	7464934
182	Chr0	7464921
183	Chr0	7499894
184	Chr0	7499891
185	Chr0	7499615

186	Chr0	7505473
187	Chr0	7505763
188	Chr0	7505823
189	Chr0	7505834
190	Chr0	7508826
191	Chr0	7537557
192	Chr0	7544590
193	Chr0	7544831
194	Chr0	7544863
195	Chr0	7572119
196	Chr0	7572155
197	Chr0	7572205
198	Chr0	7632303
199	Chr0	7632603
200	Chr0	7667821
201	Chr0	7668763
202	Chr0	7671247
203	Chr0	7671522
204	Chr0	7673059
205	Chr0	7677381
206	Chr0	7693730
207	Chr0	7714626
208	Chr0	7947672
209	Chr0	8397727
210	Chr0	8402971
211	Chr0	8418113
212	Chr0	8418044
213	Chr0	8417857
214	Chr0	8460450
215	Chr0	8460417
216	Chr0	8460252
217	Chr0	8460189
218	Chr0	8564653
219	Chr0	8580282
220	Chr0	8616954
221	Chr0	8616901
222	Chr0	8634323
223	Chr0	8634318
224	Chr0	8634299
225	Chr0	8634099
226	Chr0	8640520
227	Chr0	8640116
228	Chr0	8642973
229	Chr0	8651191
230	Chr0	8651227
231	Chr0	8651250
232	Chr0	8683745

233	Chr0	8683757
234	Chr0	8683767
235	Chr0	8683771
236	Chr0	8835564
237	Chr0	8836560
238	Chr0	8884122
239	Chr0	8884138
240	Chr0	8884425
241	Chr0	8884428
242	Chr0	9342745
243	Chr0	9453977
244	Chr0	9454146
245	Chr0	9454082
246	Chr0	9537484
247	Chr0	9537488
248	Chr0	9557442
249	Chr0	9659835
250	Chr0	9681801
251	Chr0	9681840
252	Chr0	9681864
253	Chr0	9707887
254	Chr0	9775335
255	Chr0	9775476
256	Chr0	9815088
257	Chr0	9815090
258	Chr0	9815097
259	Chr0	9815101
260	Chr0	9815102
261	Chr0	9815141
262	Chr0	9815392
263	Chr0	9815414
264	Chr0	9815457
265	Chr0	9898459
266	Chr0	9980132
267	Chr0	9979726
268	Chr0	9982016
269	Chr0	9982643
270	Chr0	9994895
271	Chr0	9996541
272	Chr0	9999789
273	Chr0	10009835
274	Chr0	10010951
275	Chr0	10012563
276	Chr0	10162339
277	Chr0	10162076
278	Chr0	10195738
279	Chr0	10209717

280	Chr0	10303063
281	Chr0	10370128
282	Chr0	10418680
283	Chr0	10444145
284	Chr0	10445996
285	Chr0	10447126
286	Chr0	10448578
287	Chr0	10483144
288	Chr0	10483095
289	Chr0	10482770
290	Chr0	10506399
291	Chr0	10543234
292	Chr0	10549444
293	Chr0	10549455
294	Chr0	10549503
295	Chr0	10557924
296	Chr0	10581592
297	Chr0	10584050
298	Chr0	10587627
299	Chr0	10588069
300	Chr0	10592303
301	Chr0	10592285
302	Chr0	10592238
303	Chr0	10592229
304	Chr0	10592217
305	Chr0	10592000
306	Chr0	10591976
307	Chr0	10591969
308	Chr0	10591921
309	Chr0	10591909
310	Chr0	10625036
311	Chr0	10632413
312	Chr0	10632258
313	Chr0	10636531
314	Chr0	10637297
315	Chr0	10981513
316	Chr0	11396655
317	Chr0	11631443
318	Chr0	11631665
319	Chr0	11690156
320	Chr0	11690188
321	Chr0	11690189
322	Chr0	11690448
323	Chr0	11692254
324	Chr0	11693912
325	Chr0	11740227
326	Chr0	11967107

327	Chr0	11967104
328	Chr0	12032616
329	Chr0	12221967
330	Chr0	12353143
331	Chr0	12448349
332	Chr0	12448353
333	Chr0	12923191
334	Chr0	12923213
335	Chr0	12923240
336	Chr0	12923573
337	Chr0	12923092
338	Chr0	12923063
339	Chr0	13014000
340	Chr0	13013718
341	Chr0	13017738
342	Chr0	13017757
343	Chr0	13143357
344	Chr0	13143336
345	Chr0	13456023
346	Chr0	13485331
347	Chr0	13486435
348	Chr0	13555327
349	Chr0	13603048
350	Chr0	14184563
351	Chr0	14227604
352	Chr0	14227949
353	Chr0	14227965
354	Chr0	14227986
355	Chr0	14227996
356	Chr0	14228028
357	Chr0	14523456
358	Chr0	14670711
359	Chr0	14670689
360	Chr0	14670679
361	Chr0	14670678
362	Chr0	14670671
363	Chr0	14670481
364	Chr0	14670432
365	Chr0	15296393
366	Chr0	15724280
367	Chr0	15723908
368	Chr0	15950857
369	Chr0	15997402
370	Chr0	15999728
371	Chr0	16224905
372	Chr0	16224878
373	Chr0	16226259

374	Chr0	16321216
375	Chr0	16321244
376	Chr0	16321254
377	Chr0	16320804
378	Chr0	16320721
379	Chr0	16394072
380	Chr0	16606278
381	Chr0	16606298
382	Chr0	16609038
383	Chr0	16609278
384	Chr0	16609287
385	Chr0	16719096
386	Chr0	16817864
387	Chr0	17122899
388	Chr0	17163868
389	Chr0	17249268
390	Chr0	17249219
391	Chr0	17277993
392	Chr0	17508544
393	Chr0	17508161
394	Chr0	17649582
395	Chr0	17771370
396	Chr0	17885850
397	Chr0	17885833
398	Chr0	17885831
399	Chr0	17885586
400	Chr0	17885564
401	Chr0	17885529
402	Chr0	17885519
403	Chr0	17885501
404	Chr0	17885494
405	Chr0	17885492
406	Chr0	17885489
407	Chr0	17885481
408	Chr0	18342384
409	Chr0	19065250
410	Chr0	19156150
411	Chr0	19156162
412	Chr0	19156163
413	Chr0	19208556
414	Chr0	19343346
415	Chr0	19602634
416	Chr0	19890629
417	Chr0	19890583
418	Chr0	20036845
419	Chr0	20037089
420	Chr0	20116817

421	Chr0	20161002
422	Chr0	20204452
423	Chr0	20248213
424	Chr0	20248222
425	Chr0	20248236
426	Chr0	20268346
427	Chr0	20726491
428	Chr0	20749787
429	Chr0	20749573
430	Chr0	20749542
431	Chr0	20749525
432	Chr0	20749494
433	Chr0	20749493
434	Chr0	20767551
435	Chr0	20767592
436	Chr0	20767828
437	Chr0	20850811
438	Chr0	20850891
439	Chr0	20850381
440	Chr1	2022150
441	Chr1	2029918
442	Chr1	2372656
443	Chr1	2372666
444	Chr1	2372302
445	Chr1	2536015
446	Chr1	2974527
447	Chr1	3668652
448	Chr1	3705308
449	Chr1	3705623
450	Chr1	5175781
451	Chr1	5175746
452	Chr1	5175442
453	Chr1	5175424
454	Chr1	5175420
455	Chr1	5175413
456	Chr1	5895113
457	Chr1	5895129
458	Chr1	5895405
459	Chr1	5895454
460	Chr1	7653446
461	Chr1	7653405
462	Chr1	7653398
463	Chr1	7653354
464	Chr1	7995961
465	Chr1	9089405
466	Chr1	9993185
467	Chr1	9993239

468	Chr1	9993243
469	Chr1	9993271
470	Chr1	9993458
471	Chr1	9993487
472	Chr1	9993493
473	Chr1	9993513
474	Chr1	9993151
475	Chr1	9993142
476	Chr1	9993112
477	Chr1	9993099
478	Chr1	9993077
479	Chr1	9992815
480	Chr1	9992789
481	Chr1	10320106
482	Chr1	10423750
483	Chr1	10614186
484	Chr1	10614239
485	Chr1	10614249
486	Chr1	10893580
487	Chr1	10893525
488	Chr1	10893177
489	Chr1	10893171
490	Chr1	10893162
491	Chr1	12001594
492	Chr1	14060368
493	Chr1	15146163
494	Chr1	16812324
495	Chr1	16812650
496	Chr1	16812655
497	Chr1	18489668
498	Chr1	19267489
499	Chr1	19556234
500	Chr1	19556504
501	Chr1	19556537
502	Chr1	19824501
503	Chr1	20785084
504	Chr1	22766273
505	Chr1	23194589
506	Chr1	23409639
507	Chr1	25247020
508	Chr1	25745258
509	Chr1	26046366
510	Chr1	27114549
511	Chr1	29916447
512	Chr1	30286327
513	Chr1	30659769
514	Chr1	31678373

515	Chr1	32758324
516	Chr1	35607374
517	Chr1	35854195
518	Chr1	35860978
519	Chr1	35861193
520	Chr1	35951024
521	Chr1	36929319
522	Chr1	37074431
523	Chr1	39006718
524	Chr1	39019236
525	Chr1	39473278
526	Chr1	39664377
527	Chr1	39664375
528	Chr1	39664105
529	Chr1	39664059
530	Chr1	39664047
531	Chr1	39664024
532	Chr1	40458579
533	Chr1	42274869
534	Chr1	43656655
535	Chr1	43723492
536	Chr1	43965251
537	Chr1	43965253
538	Chr1	43965268
539	Chr1	43965275
540	Chr1	43965291
541	Chr1	43965309
542	Chr1	43965501
543	Chr1	43965566
544	Chr1	43965602
545	Chr1	44426871
546	Chr1	44479894
547	Chr1	44479920
548	Chr1	44480140
549	Chr1	44480148
550	Chr1	46261287
551	Chr1	46566203
552	Chr1	50800077
553	Chr1	51076232
554	Chr1	51080516
555	Chr1	51080057
556	Chr1	52042374
557	Chr1	52184376
558	Chr1	53207625
559	Chr1	53313743
560	Chr1	53313679
561	Chr1	53425943

562	Chr1	54245393
563	Chr1	55541209
564	Chr1	56028578
565	Chr1	57250352
566	Chr1	57274541
567	Chr1	57274555
568	Chr1	57274556
569	Chr1	57274561
570	Chr1	57274892
571	Chr1	57274895
572	Chr1	57274919
573	Chr1	57274944
574	Chr1	57274988
575	Chr1	59131366
576	Chr1	59131365
577	Chr1	59969937
578	Chr1	60345014
579	Chr1	61625125
580	Chr1	61871602
581	Chr1	62486083
582	Chr1	62655363
583	Chr1	62921991
584	Chr1	63818879
585	Chr1	63819200
586	Chr1	63819209
587	Chr1	63819221
588	Chr1	63819222
589	Chr1	63819226
590	Chr1	65491623
591	Chr1	65620424
592	Chr1	65935517
593	Chr1	65935437
594	Chr1	65935420
595	Chr1	65935419
596	Chr1	65935175
597	Chr1	68187600
598	Chr1	68322769
599	Chr1	69166549
600	Chr1	69713473
601	Chr1	69713481
602	Chr1	69713699
603	Chr1	69739892
604	Chr1	70403658
605	Chr1	70813825
606	Chr1	70813794
607	Chr1	70813791
608	Chr1	71033764

609	Chr1	71033833
610	Chr1	71033850
611	Chr1	71033853
612	Chr1	71034134
613	Chr1	73024757
614	Chr1	73120228
615	Chr1	73120585
616	Chr1	73120149
617	Chr1	73120086
618	Chr1	73120085
619	Chr1	73119804
620	Chr1	73889989
621	Chr1	75417239
622	Chr1	75417237
623	Chr1	75417203
624	Chr1	76977085
625	Chr1	76977053
626	Chr1	76977046
627	Chr1	76977031
628	Chr1	76977030
629	Chr1	76977005
630	Chr1	76976981
631	Chr1	76976834
632	Chr1	76976818
633	Chr1	76976786
634	Chr1	76976779
635	Chr1	77228457
636	Chr1	80981816
637	Chr1	80981901
638	Chr1	80986337
639	Chr1	81018744
640	Chr1	81133741
641	Chr1	81152869
642	Chr1	81152887
643	Chr1	81153217
644	Chr1	81153287
645	Chr1	81192988
646	Chr1	82714626
647	Chr1	82795063
648	Chr1	82963632
649	Chr1	82963284
650	Chr1	83079197
651	Chr1	83584640
652	Chr1	86623050
653	Chr1	90100552
654	Chr1	91317730
655	Chr1	93383378

656	Chr1	95014072
657	Chr1	95051879
658	Chr1	95311282
659	Chr1	95311246
660	Chr1	95310961
661	Chr1	95310941
662	Chr1	95320224
663	Chr1	95320194
664	Chr1	95320186
665	Chr1	95319997
666	Chr1	95319995
667	Chr1	95332619
668	Chr1	95332624
669	Chr1	95332640
670	Chr1	95332832
671	Chr1	95332838
672	Chr1	95332865
673	Chr1	95332919
674	Chr1	95332933
675	Chr1	95432481
676	Chr1	95432482
677	Chr1	95432499
678	Chr1	95434357
679	Chr1	95434631
680	Chr1	95525993
681	Chr1	95525976
682	Chr1	95525961
683	Chr1	95525757
684	Chr1	95546262
685	Chr1	95568243
686	Chr1	95568225
687	Chr1	95568208
688	Chr1	95567922
689	Chr1	95690010
690	Chr1	95689649
691	Chr1	95692938
692	Chr1	95692925
693	Chr1	95692905
694	Chr1	95692892
695	Chr1	95692883
696	Chr1	95692667
697	Chr1	95692655
698	Chr1	95692653
699	Chr1	95769120
700	Chr1	95769188
701	Chr1	95769201
702	Chr1	95769514

703	Chr1	95769529
704	Chr1	95900540
705	Chr1	95941433
706	Chr1	95941045
707	Chr1	95945903
708	Chr1	95945583
709	Chr1	95945555
710	Chr1	96043988
711	Chr1	96043949
712	Chr1	96043870
713	Chr1	96043542
714	Chr1	96048491
715	Chr1	96048742
716	Chr1	96048804
717	Chr1	96119480
718	Chr1	96119453
719	Chr1	96119210
720	Chr1	96119178
721	Chr1	96119134
722	Chr1	96124712
723	Chr1	96124745
724	Chr1	96124928
725	Chr1	96124935
726	Chr1	96124944
727	Chr1	96124971
728	Chr1	96124989
729	Chr1	96124999
730	Chr1	96127128
731	Chr1	96127086
732	Chr1	96126851
733	Chr1	96126806
734	Chr1	96126785
735	Chr1	96251529
736	Chr1	96251543
737	Chr1	96251570
738	Chr1	96251572
739	Chr1	96251573
740	Chr1	96251582
741	Chr1	96251593
742	Chr1	96251815
743	Chr1	96277860
744	Chr1	96277905
745	Chr1	96306527
746	Chr1	96306524
747	Chr1	96306489
748	Chr1	96388980
749	Chr1	96388978

750	Chr1	96388975
751	Chr1	96388961
752	Chr1	96388693
753	Chr1	96428850
754	Chr1	96428890
755	Chr1	96429255
756	Chr1	96429282
757	Chr1	96429310
758	Chr1	96448745
759	Chr1	96448652
760	Chr1	96448649
761	Chr1	96495104
762	Chr1	96495410
763	Chr1	96529193
764	Chr1	96529176
765	Chr1	96529131
766	Chr1	96619054
767	Chr1	96619044
768	Chr1	96652740
769	Chr1	96652751
770	Chr1	96681990
771	Chr1	96682012
772	Chr1	96682015
773	Chr1	96682032
774	Chr1	96682048
775	Chr1	96682050
776	Chr1	96682054
777	Chr1	96682061
778	Chr1	96682066
779	Chr1	96682394
780	Chr1	96682400
781	Chr1	96682402
782	Chr1	96682426
783	Chr1	96685360
784	Chr1	96694134
785	Chr1	96694144
786	Chr1	96694191
787	Chr1	96694461
788	Chr1	96694491
789	Chr1	96694512
790	Chr1	96809236
791	Chr1	96809266
792	Chr1	96809280
793	Chr1	96809297
794	Chr1	96809549
795	Chr1	96919589
796	Chr1	96919573

797	Chr1	96919543
798	Chr1	96919534
799	Chr1	96919210
800	Chr1	96919208
801	Chr1	96919134
802	Chr1	96939507
803	Chr1	96939488
804	Chr1	97026510
805	Chr1	97026519
806	Chr1	97026527
807	Chr1	97026575
808	Chr1	97026576
809	Chr1	97026582
810	Chr1	97026588
811	Chr1	97026887
812	Chr1	97111785
813	Chr1	97111813
814	Chr1	97111826
815	Chr1	97112132
816	Chr1	97112135
817	Chr1	97112137
818	Chr1	97112144
819	Chr1	97113522
820	Chr1	97113556
821	Chr1	97113562
822	Chr1	97113606
823	Chr1	97113847
824	Chr1	97113909
825	Chr1	97152148
826	Chr1	97152095
827	Chr1	97152094
828	Chr1	97152087
829	Chr1	97152069
830	Chr1	97225993
831	Chr1	97267345
832	Chr1	97267600
833	Chr1	97267669
834	Chr1	97267316
835	Chr1	97267285
836	Chr1	97267281
837	Chr1	97267259
838	Chr1	97267243
839	Chr1	97267239
840	Chr1	97267101
841	Chr1	97267056
842	Chr1	97268955
843	Chr1	97269007

844	Chr1	97269167
845	Chr1	97298317
846	Chr1	97298319
847	Chr1	97298330
848	Chr1	97298333
849	Chr1	97298337
850	Chr1	97298338
851	Chr1	97298531
852	Chr1	97298552
853	Chr1	97299328
854	Chr1	97299273
855	Chr1	97298919
856	Chr1	97332589
857	Chr1	97332525
858	Chr1	97333762
859	Chr1	97333405
860	Chr1	97375035
861	Chr1	97375096
862	Chr1	97375337
863	Chr1	97375345
864	Chr1	97375395
865	Chr1	97375415
866	Chr1	97375416
867	Chr1	97375417
868	Chr1	97395517
869	Chr1	97395531
870	Chr1	97448267
871	Chr1	97448234
872	Chr1	97448002
873	Chr1	97447963
874	Chr1	97468748
875	Chr1	97468746
876	Chr1	97468689
877	Chr1	97468484
878	Chr1	97468435
879	Chr1	97469360
880	Chr1	97469376
881	Chr1	97469608
882	Chr1	97469656
883	Chr1	97470048
884	Chr1	97474086
885	Chr1	97474105
886	Chr1	97474125
887	Chr1	97474263
888	Chr1	97474309
889	Chr1	97535818
890	Chr1	97557018

891	Chr1	97556973
892	Chr1	97556967
893	Chr1	97556634
894	Chr1	97584668
895	Chr1	97584993
896	Chr1	97585034
897	Chr1	97592411
898	Chr1	97592386
899	Chr1	97592195
900	Chr1	97596440
901	Chr1	97596423
902	Chr1	97596413
903	Chr1	97596393
904	Chr1	97596142
905	Chr1	97596137
906	Chr1	97596075
907	Chr1	97596069
908	Chr1	97630004
909	Chr1	97629993
910	Chr1	97629931
911	Chr1	97656763
912	Chr1	97656779
913	Chr1	97656785
914	Chr1	97721902
915	Chr1	97721908
916	Chr1	97756450
917	Chr1	97756360
918	Chr1	97756192
919	Chr1	97756114
920	Chr1	97765083
921	Chr1	97765107
922	Chr1	97765148
923	Chr1	97764992
924	Chr1	97764969
925	Chr1	97764965
926	Chr1	97811116
927	Chr1	97811150
928	Chr1	97811332
929	Chr1	97944366
930	Chr1	97944250
931	Chr1	98031294
932	Chr1	98031295
933	Chr1	98031366
934	Chr1	98031513
935	Chr1	98031585
936	Chr1	98040875
937	Chr1	98078188

938	Chr1	98078152
939	Chr1	98110705
940	Chr1	98110700
941	Chr1	98110681
942	Chr1	98110675
943	Chr1	98110674
944	Chr1	98137504
945	Chr1	98137525
946	Chr1	98137532
947	Chr1	98137580
948	Chr1	98137592
949	Chr1	98137830
950	Chr1	98193345
951	Chr1	98193348
952	Chr1	98193356
953	Chr1	98193362
954	Chr1	98193414
955	Chr1	98193421
956	Chr1	98193592
957	Chr1	98193610
958	Chr1	98193623
959	Chr1	98245001
960	Chr1	98244990
961	Chr1	98244973
962	Chr1	98244930
963	Chr1	98308864
964	Chr1	98329896
965	Chr1	98329640
966	Chr1	98329620
967	Chr1	98329608
968	Chr1	98329579
969	Chr1	98348254
970	Chr1	98348242
971	Chr1	98347968
972	Chr1	98347962
973	Chr1	98351518
974	Chr2	783935
975	Chr2	959528
976	Chr2	2259141
977	Chr2	2258781
978	Chr2	3900126
979	Chr2	3900372
980	Chr2	6363881
981	Chr2	9659644
982	Chr2	9667357
983	Chr2	9698857
984	Chr2	13195661

985	Chr2	13201727
986	Chr2	13204724
987	Chr2	13218902
988	Chr2	13221266
989	Chr2	13225630
990	Chr2	13227670
991	Chr2	13227944
992	Chr2	13368905
993	Chr2	13369446
994	Chr2	14206377
995	Chr2	16545861
996	Chr2	17894143
997	Chr2	17963395
998	Chr2	18599339
999	Chr2	18599310
1000	Chr2	18599300
1001	Chr2	18599294
1002	Chr2	18599280
1003	Chr2	22302863
1004	Chr2	22432954
1005	Chr2	23077548
1006	Chr2	24000154
1007	Chr2	24000159
1008	Chr2	24000172
1009	Chr2	24000180
1010	Chr2	24000193
1011	Chr2	24000198
1012	Chr2	24000210
1013	Chr2	24000229
1014	Chr2	24000230
1015	Chr2	24000244
1016	Chr2	24000251
1017	Chr2	24000465
1018	Chr2	24000468
1019	Chr2	24000480
1020	Chr2	24000487
1021	Chr2	24000495
1022	Chr2	24000500
1023	Chr2	24000541
1024	Chr2	24000547
1025	Chr2	24000549
1026	Chr2	24000554
1027	Chr2	24340721
1028	Chr2	25039720
1029	Chr2	25039711
1030	Chr2	25039417
1031	Chr2	25039416

1032	Chr2	25039392
1033	Chr2	25039368
1034	Chr2	25039358
1035	Chr2	25039348
1036	Chr2	25259615
1037	Chr2	28340399
1038	Chr2	30340830
1039	Chr2	30384530
1040	Chr2	30418108
1041	Chr2	30433000
1042	Chr2	30489611
1043	Chr2	30583220
1044	Chr2	31041459
1045	Chr2	32321335
1046	Chr2	33624819
1047	Chr2	33624810
1048	Chr2	33624773
1049	Chr2	33624769
1050	Chr2	33624744
1051	Chr2	33624732
1052	Chr2	33624730
1053	Chr2	33624503
1054	Chr2	33624465
1055	Chr2	33633900
1056	Chr2	33651696
1057	Chr2	34910876
1058	Chr2	35010031
1059	Chr2	35063588
1060	Chr2	36730471
1061	Chr2	36776298
1062	Chr2	36865494
1063	Chr2	37588582
1064	Chr2	37588622
1065	Chr2	37908048
1066	Chr2	40007236
1067	Chr2	40036322
1068	Chr2	40160666
1069	Chr2	40229416
1070	Chr2	40269150
1071	Chr2	40269160
1072	Chr2	40269195
1073	Chr2	40268817
1074	Chr2	40283304
1075	Chr2	40619917
1076	Chr2	40620395
1077	Chr2	40694542
1078	Chr2	40693720

1079	Chr2	47612205
1080	Chr2	48114799
1081	Chr2	48137321
1082	Chr2	49099719
1083	Chr2	49154656
1084	Chr2	49327150
1085	Chr2	49396385
1086	Chr2	49397841
1087	Chr2	49397518
1088	Chr2	49399755
1089	Chr2	49400049
1090	Chr2	49400070
1091	Chr2	49403148
1092	Chr2	49403458
1093	Chr2	49428214
1094	Chr2	49429284
1095	Chr2	49429583
1096	Chr2	49438901
1097	Chr2	49489767
1098	Chr2	49521106
1099	Chr2	50107254
1100	Chr2	50440964
1101	Chr2	50440957
1102	Chr2	50440942
1103	Chr2	50440937
1104	Chr2	50440935
1105	Chr2	50440926
1106	Chr2	50440918
1107	Chr2	50440916
1108	Chr2	50440875
1109	Chr2	50440871
1110	Chr3	50824
1111	Chr3	50804
1112	Chr3	50803
1113	Chr3	107476
1114	Chr3	212543
1115	Chr3	212816
1116	Chr3	368422
1117	Chr3	368641
1118	Chr3	455542
1119	Chr3	455538
1120	Chr3	455537
1121	Chr3	455216
1122	Chr3	455159
1123	Chr3	515903
1124	Chr3	968615
1125	Chr3	2123758

1126	Chr3	3037310
1127	Chr3	3037326
1128	Chr3	3037329
1129	Chr3	3037356
1130	Chr3	4047503
1131	Chr3	4047502
1132	Chr3	4047032
1133	Chr3	4655937
1134	Chr3	5227471
1135	Chr3	6031546
1136	Chr3	6031293
1137	Chr3	6031281
1138	Chr3	6416534
1139	Chr3	6478091
1140	Chr3	6478086
1141	Chr3	6949380
1142	Chr3	6949676
1143	Chr3	6949684
1144	Chr3	6949709
1145	Chr3	6949332
1146	Chr3	9847183
1147	Chr3	9847155
1148	Chr3	9847133
1149	Chr3	9847126
1150	Chr3	9847109
1151	Chr3	9846841
1152	Chr3	11277304
1153	Chr3	11276950
1154	Chr3	11276940
1155	Chr3	11276924
1156	Chr3	11276917
1157	Chr3	12568339
1158	Chr3	13379069
1159	Chr3	18080364
1160	Chr3	18081331
1161	Chr3	18086214
1162	Chr3	18086601
1163	Chr3	18102733
1164	Chr3	18102723
1165	Chr3	18418512
1166	Chr3	18418260
1167	Chr3	18696490
1168	Chr3	18696522
1169	Chr3	18696863
1170	Chr3	18696895
1171	Chr3	19787239
1172	Chr3	22500817

1173	Chr3	22500849
1174	Chr3	22500856
1175	Chr3	22500876
1176	Chr3	22501183
1177	Chr3	22501190
1178	Chr3	22501202
1179	Chr3	22501239
1180	Chr3	22501243
1181	Chr3	22501266
1182	Chr3	22501268
1183	Chr3	22501275
1184	Chr3	27803873
1185	Chr3	27803581
1186	Chr3	30230326
1187	Chr3	32880017
1188	Chr3	35755707
1189	Chr3	35756066
1190	Chr3	35756067
1191	Chr3	39603132
1192	Chr3	50054734
1193	Chr3	51757112
1194	Chr3	51757424
1195	Chr3	51757441
1196	Chr3	55170653
1197	Chr3	56095858
1198	Chr3	56095732
1199	Chr3	56095716
1200	Chr3	56095689
1201	Chr3	57593220
1202	Chr3	57593216
1203	Chr3	57593177
1204	Chr3	57593170
1205	Chr3	57593165
1206	Chr3	57593164
1207	Chr3	57592923
1208	Chr3	57592921
1209	Chr3	57592910
1210	Chr3	57592900
1211	Chr3	57592880
1212	Chr3	57592867
1213	Chr3	57592858
1214	Chr3	57592850
1215	Chr3	57592847
1216	Chr3	60951550
1217	Chr3	63543355
1218	Chr3	64633255
1219	Chr3	64633560

1220	Chr3	64633579
1221	Chr3	64642725
1222	Chr3	64642755
1223	Chr3	64878737
1224	Chr3	64883828
1225	Chr3	64917120
1226	Chr3	64917159
1227	Chr3	64962017
1228	Chr3	64961969
1229	Chr3	64961713
1230	Chr3	64985315
1231	Chr3	65004659
1232	Chr3	65012529
1233	Chr3	65115918
1234	Chr3	65247902
1235	Chr3	65247987
1236	Chr3	65306977
1237	Chr3	65346875
1238	Chr3	65346735
1239	Chr3	65369991
1240	Chr3	65420272
1241	Chr3	65527596
1242	Chr3	65569737
1243	Chr3	65570017
1244	Chr3	65641118
1245	Chr3	65762125
1246	Chr3	65778035
1247	Chr3	65817864
1248	Chr3	65817596
1249	Chr3	65892596
1250	Chr3	65985153
1251	Chr3	66019137
1252	Chr3	66053284
1253	Chr3	66067306
1254	Chr3	66071183
1255	Chr3	66070934
1256	Chr3	66070883
1257	Chr3	66075969
1258	Chr3	66075749
1259	Chr3	66075748
1260	Chr3	66196222
1261	Chr3	66196224
1262	Chr3	66196241
1263	Chr3	66213738
1264	Chr3	66213793
1265	Chr3	66254937
1266	Chr3	68262180

1267	Chr3	68262491
1268	Chr3	68282138
1269	Chr3	68282081
1270	Chr3	68317793
1271	Chr3	68317830
1272	Chr3	68317888
1273	Chr3	68688687
1274	Chr3	68688689
1275	Chr4	4382840
1276	Chr4	5294087
1277	Chr4	5294097
1278	Chr4	5884406
1279	Chr4	7278790
1280	Chr4	7296694
1281	Chr4	7335252
1282	Chr4	7335263
1283	Chr4	7458318
1284	Chr4	7458289
1285	Chr4	7457903
1286	Chr4	7624290
1287	Chr4	7697429
1288	Chr4	7697486
1289	Chr4	7807958
1290	Chr4	7808204
1291	Chr4	7808231
1292	Chr4	7812571
1293	Chr4	7812529
1294	Chr4	7812135
1295	Chr4	7819396
1296	Chr4	7819397
1297	Chr4	7885750
1298	Chr4	7885951
1299	Chr4	7885972
1300	Chr4	7885991
1301	Chr4	7886023
1302	Chr4	7885587
1303	Chr4	7885316
1304	Chr4	7943697
1305	Chr4	7943686
1306	Chr4	7943455
1307	Chr4	7943442
1308	Chr4	7951826
1309	Chr4	8091076
1310	Chr4	8091077
1311	Chr4	8091103
1312	Chr4	8091115
1313	Chr4	8091130

1314	Chr4	8091304
1315	Chr4	8091331
1316	Chr4	8091359
1317	Chr4	8091386
1318	Chr4	8092621
1319	Chr4	8092631
1320	Chr4	8092640
1321	Chr4	8092711
1322	Chr4	8102544
1323	Chr4	8102575
1324	Chr4	8102598
1325	Chr4	8102851
1326	Chr4	8102893
1327	Chr4	8102894
1328	Chr4	8363217
1329	Chr4	8363177
1330	Chr4	8363131
1331	Chr4	8362890
1332	Chr4	8362874
1333	Chr4	8362843
1334	Chr4	8362828
1335	Chr4	8369826
1336	Chr4	8370079
1337	Chr4	8370089
1338	Chr4	8369765
1339	Chr4	8369745
1340	Chr4	8369710
1341	Chr4	8369704
1342	Chr4	8369494
1343	Chr4	8369443
1344	Chr4	8406174
1345	Chr4	8466767
1346	Chr4	8534305
1347	Chr4	8534306
1348	Chr4	8534351
1349	Chr4	8534606
1350	Chr4	8534614
1351	Chr4	8534649
1352	Chr4	8543456
1353	Chr4	8543468
1354	Chr4	8543478
1355	Chr4	8543824
1356	Chr4	8543839
1357	Chr4	8543882
1358	Chr4	8595311
1359	Chr4	8595678
1360	Chr4	8595731

1361	Chr4	8614827
1362	Chr4	8614461
1363	Chr4	8617033
1364	Chr4	8617466
1365	Chr4	8851835
1366	Chr4	8851766
1367	Chr4	8851765
1368	Chr4	9031703
1369	Chr4	9031758
1370	Chr4	9031961
1371	Chr4	9031970
1372	Chr4	9031983
1373	Chr4	9033341
1374	Chr4	9033291
1375	Chr4	9033025
1376	Chr4	9032974
1377	Chr4	9093924
1378	Chr4	9093963
1379	Chr4	9216409
1380	Chr4	9216708
1381	Chr4	9224957
1382	Chr4	9224956
1383	Chr4	9224938
1384	Chr4	9224903
1385	Chr4	9224690
1386	Chr4	9224644
1387	Chr4	9251524
1388	Chr4	9251277
1389	Chr4	9251237
1390	Chr4	9251231
1391	Chr4	9251222
1392	Chr4	9269559
1393	Chr4	9269533
1394	Chr4	9269479
1395	Chr4	9269284
1396	Chr4	9306419
1397	Chr4	9306411
1398	Chr4	9306399
1399	Chr4	9306342
1400	Chr4	9305975
1401	Chr4	9305952
1402	Chr4	9343603
1403	Chr4	9343600
1404	Chr4	9343564
1405	Chr4	9343308
1406	Chr4	9343303
1407	Chr4	9415457

1408	Chr4	9415537
1409	Chr4	9415426
1410	Chr4	9415402
1411	Chr4	9415344
1412	Chr4	9415339
1413	Chr4	9415168
1414	Chr4	9415112
1415	Chr4	9472907
1416	Chr4	9473246
1417	Chr4	9480796
1418	Chr4	9480810
1419	Chr4	9481076
1420	Chr4	9482476
1421	Chr4	9482562
1422	Chr4	9482807
1423	Chr4	9516743
1424	Chr4	9550315
1425	Chr4	9584087
1426	Chr4	9583667
1427	Chr4	9598795
1428	Chr4	9599115
1429	Chr4	9650495
1430	Chr4	9650772
1431	Chr4	9660761
1432	Chr4	9661027
1433	Chr4	9661045
1434	Chr4	9700723
1435	Chr4	9707150
1436	Chr4	9707196
1437	Chr4	9707212
1438	Chr4	9707420
1439	Chr4	9707470
1440	Chr4	9728107
1441	Chr4	9728136
1442	Chr4	9728137
1443	Chr4	9728488
1444	Chr4	9728494
1445	Chr4	9728526
1446	Chr4	9728061
1447	Chr4	9728022
1448	Chr4	9742444
1449	Chr4	9742451
1450	Chr4	9742747
1451	Chr4	9742834
1452	Chr4	9758416
1453	Chr4	9758441
1454	Chr4	9801532

1455	Chr4	9801772
1456	Chr4	9801805
1457	Chr4	9801810
1458	Chr4	9801819
1459	Chr4	9819731
1460	Chr4	9819722
1461	Chr4	9819696
1462	Chr4	9839752
1463	Chr4	9839780
1464	Chr4	9840104
1465	Chr4	9840117
1466	Chr4	9840142
1467	Chr4	9839694
1468	Chr4	9839683
1469	Chr4	9839640
1470	Chr4	9839424
1471	Chr4	9839401
1472	Chr4	9839354
1473	Chr4	9839336
1474	Chr4	9912134
1475	Chr4	9912139
1476	Chr4	9912159
1477	Chr4	9912165
1478	Chr4	9912451
1479	Chr4	10396983
1480	Chr4	10396985
1481	Chr4	10397001
1482	Chr4	10397009
1483	Chr4	10397025
1484	Chr4	10397215
1485	Chr4	10396845
1486	Chr4	10396644
1487	Chr4	10396610
1488	Chr4	10396600
1489	Chr4	10396594
1490	Chr4	10396582
1491	Chr4	10396559
1492	Chr4	10436906
1493	Chr4	10436954
1494	Chr4	10436961
1495	Chr4	10437246
1496	Chr4	10437249
1497	Chr4	10437251
1498	Chr4	10437276
1499	Chr4	10446664
1500	Chr4	10495782
1501	Chr4	10495727

1502	Chr4	10544717
1503	Chr4	10544397
1504	Chr4	10544386
1505	Chr4	10563010
1506	Chr4	10563053
1507	Chr4	10563070
1508	Chr4	10563078
1509	Chr4	10563420
1510	Chr4	10577625
1511	Chr4	10605448
1512	Chr4	10605471
1513	Chr4	10605476
1514	Chr4	10605849
1515	Chr4	10605886
1516	Chr4	10605897
1517	Chr4	10621325
1518	Chr4	10621369
1519	Chr4	10621553
1520	Chr4	10621559
1521	Chr4	10621582
1522	Chr4	10621593
1523	Chr4	10621600
1524	Chr4	10681052
1525	Chr4	10681077
1526	Chr4	10681085
1527	Chr4	10797207
1528	Chr4	10797249
1529	Chr4	10797255
1530	Chr4	10797259
1531	Chr4	10797291
1532	Chr4	10797483
1533	Chr4	10797507
1534	Chr4	10797514
1535	Chr4	10903801
1536	Chr4	10903982
1537	Chr4	10903991
1538	Chr4	10904034
1539	Chr4	10914427
1540	Chr4	10914507
1541	Chr4	10914512
1542	Chr4	10914747
1543	Chr4	10914760
1544	Chr4	10914812
1545	Chr4	10914089
1546	Chr4	10914035
1547	Chr4	10914021
1548	Chr4	10927689

1549	Chr4	10927700
1550	Chr4	10927703
1551	Chr4	10927719
1552	Chr4	10927859
1553	Chr4	10927913
1554	Chr4	10960813
1555	Chr4	10960792
1556	Chr4	10960602
1557	Chr4	10960544
1558	Chr4	10974836
1559	Chr4	10974879
1560	Chr4	10975132
1561	Chr4	10975196
1562	Chr4	11002257
1563	Chr4	11002240
1564	Chr4	11002214
1565	Chr4	11002196
1566	Chr4	11002188
1567	Chr4	11002022
1568	Chr4	11002010
1569	Chr4	11001962
1570	Chr4	11003390
1571	Chr4	11003391
1572	Chr4	11003682
1573	Chr4	11003686
1574	Chr4	11003726
1575	Chr4	11003749
1576	Chr4	11003757
1577	Chr4	11007236
1578	Chr4	11007248
1579	Chr4	11007280
1580	Chr4	11007465
1581	Chr4	11007482
1582	Chr4	11007483
1583	Chr4	11071194
1584	Chr4	11071114
1585	Chr4	11070912
1586	Chr4	11070905
1587	Chr4	11070847
1588	Chr4	11203766
1589	Chr4	11203800
1590	Chr4	11204040
1591	Chr4	11204052
1592	Chr4	11204113
1593	Chr4	11327533
1594	Chr4	11327571
1595	Chr4	11327599

1596	Chr4	11327925
1597	Chr4	11352198
1598	Chr4	11352557
1599	Chr4	11352607
1600	Chr4	11352615
1601	Chr4	11352633
1602	Chr4	11356124
1603	Chr4	11356167
1604	Chr4	11356186
1605	Chr4	11356397
1606	Chr4	11356437
1607	Chr4	11362314
1608	Chr4	11362294
1609	Chr4	11362286
1610	Chr4	11362244
1611	Chr4	11369563
1612	Chr4	11369643
1613	Chr4	11369930
1614	Chr4	11369522
1615	Chr4	11369481
1616	Chr4	11369238
1617	Chr4	11369231
1618	Chr4	11386760
1619	Chr4	11386729
1620	Chr4	11386726
1621	Chr4	11391741
1622	Chr4	11403375
1623	Chr4	11403380
1624	Chr4	11403685
1625	Chr4	11418273
1626	Chr4	11418310
1627	Chr4	11418338
1628	Chr4	11418345
1629	Chr4	11418665
1630	Chr4	11418666
1631	Chr4	11418708
1632	Chr4	11418716
1633	Chr4	11433471
1634	Chr4	11433750
1635	Chr4	11433763
1636	Chr4	11433816
1637	Chr4	11432997
1638	Chr4	11435686
1639	Chr4	11435354
1640	Chr4	11435351
1641	Chr4	11435341
1642	Chr4	11438569

1643	Chr4	11444496
1644	Chr4	11444533
1645	Chr4	11444535
1646	Chr4	11444581
1647	Chr4	11444790
1648	Chr4	11444792
1649	Chr4	11444829
1650	Chr4	11444841
1651	Chr4	11444850
1652	Chr4	11535375
1653	Chr4	11535165
1654	Chr4	11561532
1655	Chr4	11561548
1656	Chr4	11561786
1657	Chr4	11574476
1658	Chr4	12117103
1659	Chr4	12143513
1660	Chr4	12143225
1661	Chr4	12192619
1662	Chr4	12192581
1663	Chr4	12201651
1664	Chr4	12201354
1665	Chr4	12201346
1666	Chr4	12201326
1667	Chr4	12268120
1668	Chr4	12268094
1669	Chr4	12267907
1670	Chr4	12267860
1671	Chr4	12301309
1672	Chr4	12306147
1673	Chr4	12306125
1674	Chr4	12472527
1675	Chr4	12472558
1676	Chr4	12493321
1677	Chr4	12493310
1678	Chr4	12493009
1679	Chr4	12550483
1680	Chr4	12550482
1681	Chr4	12550302
1682	Chr4	12618353
1683	Chr4	12623308
1684	Chr4	12623288
1685	Chr4	12623278
1686	Chr4	12622861
1687	Chr4	12622858
1688	Chr4	12629132
1689	Chr4	12629074

1690	Chr4	12634262
1691	Chr4	12634591
1692	Chr4	12634623
1693	Chr4	12634630
1694	Chr4	12634494
1695	Chr4	12634433
1696	Chr4	12637341
1697	Chr4	12637380
1698	Chr4	12637741
1699	Chr4	12637746
1700	Chr4	12637764
1701	Chr4	12637272
1702	Chr4	12637036
1703	Chr4	12636961
1704	Chr4	12636943
1705	Chr4	12721831
1706	Chr4	12789257
1707	Chr4	12789271
1708	Chr4	12789326
1709	Chr4	12789337
1710	Chr4	12789352
1711	Chr4	12789566
1712	Chr4	12789573
1713	Chr4	12804311
1714	Chr4	12804316
1715	Chr4	12804333
1716	Chr4	12804364
1717	Chr4	12804389
1718	Chr4	12804650
1719	Chr4	12804658
1720	Chr4	12895811
1721	Chr4	12896118
1722	Chr4	12895737
1723	Chr4	12895700
1724	Chr4	12895675
1725	Chr4	12912476
1726	Chr4	12912488
1727	Chr4	12912560
1728	Chr4	12917782
1729	Chr4	12917466
1730	Chr4	12930071
1731	Chr4	12930085
1732	Chr4	12930305
1733	Chr4	12930308
1734	Chr4	12930346
1735	Chr4	12930366
1736	Chr4	12952505

1737	Chr4	12952499
1738	Chr4	12952454
1739	Chr4	12952252
1740	Chr4	12952231
1741	Chr4	12952191
1742	Chr4	12983158
1743	Chr4	12983174
1744	Chr4	12986580
1745	Chr4	12986819
1746	Chr4	12986833
1747	Chr4	12986861
1748	Chr4	13003216
1749	Chr4	13003279
1750	Chr4	13003661
1751	Chr4	13003672
1752	Chr4	13033224
1753	Chr4	13033264
1754	Chr4	13033496
1755	Chr4	13367764
1756	Chr4	13378921
1757	Chr4	13378956
1758	Chr4	13387566
1759	Chr4	13416943
1760	Chr4	13416521
1761	Chr4	13416499
1762	Chr4	13526855
1763	Chr4	13526919
1764	Chr4	13527096
1765	Chr4	13527118
1766	Chr4	13550671
1767	Chr4	13550720
1768	Chr4	13550731
1769	Chr4	13550981
1770	Chr4	13551000
1771	Chr4	13583974
1772	Chr4	13675571
1773	Chr4	13675579
1774	Chr4	13675597
1775	Chr4	13675880
1776	Chr4	13675905
1777	Chr4	13675913
1778	Chr4	13675926
1779	Chr4	13675936
1780	Chr4	13675951
1781	Chr4	13679300
1782	Chr4	13779881
1783	Chr4	13779858

1784	Chr4	13810193
1785	Chr4	14274141
1786	Chr4	14274162
1787	Chr4	14274180
1788	Chr4	14279261
1789	Chr4	14279265
1790	Chr4	14279272
1791	Chr4	14279465
1792	Chr4	14279530
1793	Chr4	14279554
1794	Chr4	14300520
1795	Chr4	14300937
1796	Chr4	14309745
1797	Chr4	14520127
1798	Chr4	14520136
1799	Chr4	14520510
1800	Chr4	14520575
1801	Chr4	14520905
1802	Chr4	14520895
1803	Chr4	14520668
1804	Chr4	14520659
1805	Chr4	14522714
1806	Chr4	14623294
1807	Chr4	14786072
1808	Chr4	14786086
1809	Chr4	14786090
1810	Chr4	14827068
1811	Chr4	14847241
1812	Chr4	14847166
1813	Chr4	14847150
1814	Chr4	14877480
1815	Chr4	14877525
1816	Chr4	14877544
1817	Chr4	14877553
1818	Chr4	14877875
1819	Chr4	14891474
1820	Chr4	14933220
1821	Chr4	15012709
1822	Chr4	15012799
1823	Chr4	15013104
1824	Chr4	15013357
1825	Chr4	15013167
1826	Chr4	15013134
1827	Chr4	15013130
1828	Chr4	15013129
1829	Chr4	15018143
1830	Chr4	15018128

1831	Chr4	15099205
1832	Chr4	15099234
1833	Chr4	15099242
1834	Chr4	15099250
1835	Chr4	15099481
1836	Chr4	15099533
1837	Chr4	15102592
1838	Chr4	15102338
1839	Chr4	15104646
1840	Chr4	15135218
1841	Chr4	15135537
1842	Chr4	15135548
1843	Chr4	15135557
1844	Chr4	15135568
1845	Chr4	15135585
1846	Chr4	15135605
1847	Chr4	15135179
1848	Chr4	15135169
1849	Chr4	15135157
1850	Chr4	15135099
1851	Chr4	15135097
1852	Chr4	15134840
1853	Chr4	15134830
1854	Chr4	15148592
1855	Chr4	15148525
1856	Chr4	15148510
1857	Chr4	15163897
1858	Chr4	15163576
1859	Chr4	15163527
1860	Chr4	15163522
1861	Chr4	15191793
1862	Chr4	15191822
1863	Chr4	15191823
1864	Chr4	15253641
1865	Chr4	15253891
1866	Chr4	15253941
1867	Chr4	15341360
1868	Chr4	15341131
1869	Chr4	15341115
1870	Chr4	15341107
1871	Chr4	15341106
1872	Chr4	15341099
1873	Chr4	15420594
1874	Chr4	15452072
1875	Chr4	15451705
1876	Chr4	15459796
1877	Chr4	15520918

1878	Chr4	15520868
1879	Chr4	15533475
1880	Chr4	15533499
1881	Chr4	15550828
1882	Chr4	15551083
1883	Chr4	15551156
1884	Chr4	15551159
1885	Chr4	15551177
1886	Chr4	15550711
1887	Chr4	15550496
1888	Chr4	15666041
1889	Chr4	15666031
1890	Chr4	15700778
1891	Chr4	15700598
1892	Chr4	15700571
1893	Chr4	15759714
1894	Chr4	15806824
1895	Chr4	15806888
1896	Chr4	15806911
1897	Chr4	15818451
1898	Chr4	15818502
1899	Chr4	15818764
1900	Chr4	15818765
1901	Chr4	15818352
1902	Chr4	15818142
1903	Chr4	15833473
1904	Chr4	15833533
1905	Chr4	15899220
1906	Chr4	15967902
1907	Chr4	15978614
1908	Chr4	15978639
1909	Chr4	15978655
1910	Chr4	15978992
1911	Chr4	15979007
1912	Chr4	15979094
1913	Chr4	16001112
1914	Chr4	16001170
1915	Chr4	16001381
1916	Chr4	16001440
1917	Chr4	16007426
1918	Chr4	16007174
1919	Chr4	16007151
1920	Chr4	16007850
1921	Chr4	16007862
1922	Chr4	16018161
1923	Chr4	16107659
1924	Chr4	16107664

1925	Chr4	16107682
1926	Chr4	16108069
1927	Chr4	16129212
1928	Chr4	16129199
1929	Chr4	16129159
1930	Chr4	16129124
1931	Chr4	16163498
1932	Chr4	16186402
1933	Chr4	16186433
1934	Chr4	16186437
1935	Chr4	16186737
1936	Chr4	16186265
1937	Chr4	16185896
1938	Chr4	16202425
1939	Chr4	16216648
1940	Chr4	16304239
1941	Chr4	16355882
1942	Chr4	16355937
1943	Chr4	16380284
1944	Chr4	16419275
1945	Chr4	16419251
1946	Chr4	16419244
1947	Chr4	16459630
1948	Chr4	16459632
1949	Chr4	16459655
1950	Chr4	16463117
1951	Chr4	16567215
1952	Chr4	16567005
1953	Chr4	16567003
1954	Chr4	16566996
1955	Chr4	16661255
1956	Chr4	16661219
1957	Chr4	16661178
1958	Chr4	16660992
1959	Chr4	16769853
1960	Chr4	16769602
1961	Chr4	16769574
1962	Chr4	16783283
1963	Chr4	16783430
1964	Chr4	16783461
1965	Chr4	16818840
1966	Chr4	16818862
1967	Chr4	16819204
1968	Chr4	16819217
1969	Chr4	16840273
1970	Chr4	16840283
1971	Chr4	16841380

1972	Chr4	16841410
1973	Chr4	16841682
1974	Chr4	16841684
1975	Chr4	16841735
1976	Chr4	16847821
1977	Chr4	16847870
1978	Chr4	16889917
1979	Chr4	16889954
1980	Chr4	16900898
1981	Chr4	16900670
1982	Chr4	16900649
1983	Chr4	16907483
1984	Chr4	16907473
1985	Chr4	16907392
1986	Chr4	16907165
1987	Chr4	16907128
1988	Chr4	16920531
1989	Chr4	16920854
1990	Chr4	16920860
1991	Chr4	16995952
1992	Chr4	17009260
1993	Chr4	17012638
1994	Chr4	17012822
1995	Chr4	17012830
1996	Chr4	17012842
1997	Chr4	17012453
1998	Chr4	17012265
1999	Chr4	17059336
2000	Chr4	17165197
2001	Chr4	17165246
2002	Chr4	17165541
2003	Chr4	17165588
2004	Chr4	17184856
2005	Chr4	17184849
2006	Chr4	17184843
2007	Chr4	17184810
2008	Chr4	17184785
2009	Chr4	17190085
2010	Chr4	17190029
2011	Chr4	17190017
2012	Chr4	17189716
2013	Chr4	17189641
2014	Chr4	17217966
2015	Chr4	17236088
2016	Chr4	17236115
2017	Chr4	17236149
2018	Chr4	17236156

2019	Chr4	17236363
2020	Chr4	17244554
2021	Chr4	17244493
2022	Chr4	17244270
2023	Chr4	17244267
2024	Chr4	17258264
2025	Chr4	17257892
2026	Chr4	17257885
2027	Chr4	17257876
2028	Chr4	17259598
2029	Chr4	17259343
2030	Chr4	17259342
2031	Chr4	17259326
2032	Chr4	17277694
2033	Chr4	17278085
2034	Chr4	17278091
2035	Chr4	17284732
2036	Chr4	17284947
2037	Chr4	17284979
2038	Chr4	17284984
2039	Chr4	17337041
2040	Chr4	17337040
2041	Chr4	17337031
2042	Chr4	17337022
2043	Chr4	17336967
2044	Chr4	17336788
2045	Chr4	17336765
2046	Chr4	17345189
2047	Chr4	17345190
2048	Chr4	17345234
2049	Chr4	17345451
2050	Chr4	17366878
2051	Chr4	17367267
2052	Chr4	17412011
2053	Chr4	17411974
2054	Chr4	17411967
2055	Chr4	17411705
2056	Chr4	17411669
2057	Chr4	17411652
2058	Chr4	17460773
2059	Chr4	17460694
2060	Chr4	17490595
2061	Chr4	17509269
2062	Chr4	17509302
2063	Chr4	17509304
2064	Chr4	17509607
2065	Chr4	17551105

2066	Chr4	17563849
2067	Chr4	17563770
2068	Chr4	17786865
2069	Chr4	17787136
2070	Chr4	17833326
2071	Chr4	17833316
2072	Chr4	17833083
2073	Chr4	17833068
2074	Chr4	17833039
2075	Chr4	17833036
2076	Chr4	17854602
2077	Chr4	17854595
2078	Chr4	17854567
2079	Chr4	17854302
2080	Chr4	17854226
2081	Chr4	17897743
2082	Chr4	17898649
2083	Chr4	17898671
2084	Chr4	17898677
2085	Chr4	17898739
2086	Chr4	17899073
2087	Chr4	17899424
2088	Chr4	17899440
2089	Chr4	17899450
2090	Chr4	17899395
2091	Chr4	17899346
2092	Chr4	17905738
2093	Chr4	17905781
2094	Chr4	17943377
2095	Chr4	17976736
2096	Chr4	17976759
2097	Chr4	17976785
2098	Chr4	17976631
2099	Chr4	17976620
2100	Chr4	17976276
2101	Chr4	17976274
2102	Chr4	17995037
2103	Chr4	17994985
2104	Chr4	17994729
2105	Chr4	17994706
2106	Chr4	17994693
2107	Chr4	18058749
2108	Chr4	18058739
2109	Chr4	18058710
2110	Chr4	18128894
2111	Chr4	18150042
2112	Chr4	18149973

2113	Chr4	18149963
2114	Chr4	18191569
2115	Chr4	18191643
2116	Chr4	18191277
2117	Chr4	18191270
2118	Chr4	18289421
2119	Chr4	18289675
2120	Chr4	18364047
2121	Chr4	18363985
2122	Chr4	18418826
2123	Chr4	18419130
2124	Chr4	18419133
2125	Chr4	18428480
2126	Chr4	18428517
2127	Chr4	18428712
2128	Chr4	18428776
2129	Chr4	18455626
2130	Chr4	18455589
2131	Chr4	18455545
2132	Chr4	18510147
2133	Chr4	18537896
2134	Chr4	18537893
2135	Chr4	18537725
2136	Chr4	18537722
2137	Chr4	18537710
2138	Chr4	18600713
2139	Chr4	18600699
2140	Chr4	18614143
2141	Chr4	18614151
2142	Chr4	18712692
2143	Chr4	18714170
2144	Chr4	18714104
2145	Chr4	18714081
2146	Chr4	18713810
2147	Chr4	18713768
2148	Chr4	18719286
2149	Chr4	18719304
2150	Chr4	18719319
2151	Chr4	18719690
2152	Chr4	18746748
2153	Chr4	18746726
2154	Chr4	18746723
2155	Chr4	18746485
2156	Chr4	18947467
2157	Chr4	18947809
2158	Chr4	18947841
2159	Chr4	18947867

2160	Chr4	18947363
2161	Chr4	18983046
2162	Chr4	18983050
2163	Chr4	18983269
2164	Chr4	19027942
2165	Chr4	19035020
2166	Chr4	19034961
2167	Chr4	19034741
2168	Chr4	19039621
2169	Chr4	19039662
2170	Chr4	19069090
2171	Chr4	19068883
2172	Chr4	19068868
2173	Chr4	19106210
2174	Chr4	19106211
2175	Chr4	19106221
2176	Chr4	19106551
2177	Chr4	19106556
2178	Chr4	19106597
2179	Chr4	19106609
2180	Chr4	19106634
2181	Chr4	19132376
2182	Chr4	19132357
2183	Chr4	19132157
2184	Chr4	19132139
2185	Chr4	19132074
2186	Chr4	19155859
2187	Chr4	19155794
2188	Chr4	19155566
2189	Chr4	19180790
2190	Chr4	19180754
2191	Chr4	19180705
2192	Chr4	19180510
2193	Chr4	19180481
2194	Chr4	19180433
2195	Chr4	19205580
2196	Chr4	19205335
2197	Chr4	19205323
2198	Chr4	19267612
2199	Chr4	19267386
2200	Chr4	19289066
2201	Chr4	19289125
2202	Chr4	19289158
2203	Chr4	19290310
2204	Chr4	19290301
2205	Chr4	19290098
2206	Chr4	19290041

2207	Chr4	19290033
2208	Chr4	19335302
2209	Chr4	19335549
2210	Chr4	19335561
2211	Chr4	19355917
2212	Chr4	19355745
2213	Chr4	19355728
2214	Chr4	19370738
2215	Chr4	19370780
2216	Chr4	19370790
2217	Chr4	19370918
2218	Chr4	19370994
2219	Chr4	19378263
2220	Chr4	19378264
2221	Chr4	19378271
2222	Chr4	19392683
2223	Chr4	19392674
2224	Chr4	19392621
2225	Chr4	19392612
2226	Chr4	19392609
2227	Chr4	19392363
2228	Chr4	19392308
2229	Chr4	19392281
2230	Chr4	19432972
2231	Chr4	19432997
2232	Chr4	19436142
2233	Chr4	19469973
2234	Chr4	19470161
2235	Chr4	19470207
2236	Chr4	19470208
2237	Chr4	19469903
2238	Chr4	19469854
2239	Chr4	19503684
2240	Chr4	19503670
2241	Chr4	19503613
2242	Chr4	19503345
2243	Chr4	19504742
2244	Chr4	19504764
2245	Chr4	19505087
2246	Chr4	19505098
2247	Chr4	19512747
2248	Chr4	19512764
2249	Chr4	19584841
2250	Chr4	19596466
2251	Chr4	19600590
2252	Chr4	19602376
2253	Chr4	19602382

2254	Chr4	19602387
2255	Chr4	19617236
2256	Chr4	19804555
2257	Chr4	19804636
2258	Chr4	19804926
2259	Chr4	19808777
2260	Chr4	19989979
2261	Chr4	19989609
2262	Chr4	20169353
2263	Chr4	20169293
2264	Chr4	20168995
2265	Chr4	20168958
2266	Chr4	20168927
2267	Chr4	20170828
2268	Chr4	20170628
2269	Chr4	20170478
2270	Chr4	20170432
2271	Chr4	20181818
2272	Chr4	20181767
2273	Chr4	20191495
2274	Chr4	20237002
2275	Chr4	20279823
2276	Chr4	20292596
2277	Chr4	20306098
2278	Chr4	20393937
2279	Chr4	20394351
2280	Chr4	20399028
2281	Chr4	20398691
2282	Chr4	20405061
2283	Chr4	20405065
2284	Chr4	20405358
2285	Chr4	20405359
2286	Chr4	20405368
2287	Chr4	20405376
2288	Chr4	20405028
2289	Chr4	20404791
2290	Chr4	20411721
2291	Chr4	20427262
2292	Chr4	20427080
2293	Chr4	20427002
2294	Chr4	20483377
2295	Chr4	20488090
2296	Chr4	20488099
2297	Chr4	20519265
2298	Chr4	20519256
2299	Chr4	20519218
2300	Chr4	20527499

2301	Chr4	20671815
2302	Chr4	20671800
2303	Chr4	20671779
2304	Chr4	20671771
2305	Chr4	20742190
2306	Chr4	20752276
2307	Chr4	20758930
2308	Chr4	20766080
2309	Chr4	20765838
2310	Chr4	20776116
2311	Chr4	20776181
2312	Chr4	20776483
2313	Chr4	20776511
2314	Chr4	20776523
2315	Chr4	20780127
2316	Chr4	20787405
2317	Chr4	20787491
2318	Chr4	20791665
2319	Chr4	20791510
2320	Chr4	20791491
2321	Chr4	20798521
2322	Chr4	20798507
2323	Chr4	20823052
2324	Chr4	20823120
2325	Chr4	20898058
2326	Chr4	20898345
2327	Chr4	20898381
2328	Chr4	20964920
2329	Chr4	20964613
2330	Chr4	20981534
2331	Chr4	20981574
2332	Chr4	20981928
2333	Chr4	20986327
2334	Chr4	20999513
2335	Chr4	21000408
2336	Chr4	21000452
2337	Chr4	20999941
2338	Chr4	20999907
2339	Chr4	20999868
2340	Chr4	21019525
2341	Chr4	21022590
2342	Chr4	21022263
2343	Chr4	21032275
2344	Chr4	21032255
2345	Chr4	21031973
2346	Chr4	21031969
2347	Chr4	21031964

2348	Chr4	21031942
2349	Chr4	21050498
2350	Chr4	21062212
2351	Chr4	21156236
2352	Chr4	21156207
2353	Chr4	21156172
2354	Chr4	21155905
2355	Chr4	21155887
2356	Chr4	21210356
2357	Chr4	21210304
2358	Chr4	21240620
2359	Chr4	21240555
2360	Chr4	21259746
2361	Chr4	21275086
2362	Chr4	21278429
2363	Chr4	21317440
2364	Chr4	21317442
2365	Chr4	21317860
2366	Chr4	21317409
2367	Chr4	21317395
2368	Chr4	21317060
2369	Chr4	21365221
2370	Chr4	21365295
2371	Chr4	21365443
2372	Chr4	21465704
2373	Chr4	21465732
2374	Chr4	21465784
2375	Chr4	21466056
2376	Chr4	21466079
2377	Chr4	21479852
2378	Chr4	21479897
2379	Chr4	21503953
2380	Chr4	21503922
2381	Chr4	21503880
2382	Chr4	21546506
2383	Chr4	21546504
2384	Chr4	21546140
2385	Chr4	21546123
2386	Chr4	21546122
2387	Chr4	21572447
2388	Chr4	21572514
2389	Chr4	21574353
2390	Chr4	21574500
2391	Chr4	21751443
2392	Chr4	21751450
2393	Chr4	21751474
2394	Chr4	21751629

2395	Chr4	21751664
2396	Chr4	21786527
2397	Chr4	21848641
2398	Chr4	21848626
2399	Chr4	21848595
2400	Chr4	21848578
2401	Chr4	21848316
2402	Chr4	21862253
2403	Chr4	21939591
2404	Chr4	21939590
2405	Chr4	21939434
2406	Chr4	21939423
2407	Chr4	21939386
2408	Chr4	21939369
2409	Chr4	21939343
2410	Chr4	21939336
2411	Chr4	22010307
2412	Chr4	22010589
2413	Chr4	22010590
2414	Chr4	22010610
2415	Chr4	22010613
2416	Chr4	22055008
2417	Chr4	22054977
2418	Chr4	22087090
2419	Chr4	22087456
2420	Chr4	22087486
2421	Chr4	22265724
2422	Chr4	22265313
2423	Chr4	22265976
2424	Chr4	22275608
2425	Chr4	22285132
2426	Chr4	22331633
2427	Chr4	22344825
2428	Chr4	22344859
2429	Chr4	22344888
2430	Chr4	22351569
2431	Chr4	22353898
2432	Chr4	22354304
2433	Chr4	22369342
2434	Chr4	22433458
2435	Chr4	22463561
2436	Chr4	22561652
2437	Chr4	22561749
2438	Chr4	22561061
2439	Chr4	22561012
2440	Chr4	22560982
2441	Chr4	22586581

2442	Chr4	22610280
2443	Chr4	22610279
2444	Chr4	22610258
2445	Chr4	22610195
2446	Chr4	22649826
2447	Chr4	22650132
2448	Chr4	22652107
2449	Chr4	22660289
2450	Chr4	22660549
2451	Chr4	22660557
2452	Chr4	22660592
2453	Chr4	22660605
2454	Chr4	22683405
2455	Chr4	22683205
2456	Chr4	22683195
2457	Chr4	22683154
2458	Chr4	22739062
2459	Chr4	22739091
2460	Chr4	22745646
2461	Chr4	22745640
2462	Chr4	22745601
2463	Chr4	22745294
2464	Chr4	22751962
2465	Chr4	22758236
2466	Chr4	22758463
2467	Chr4	22758477
2468	Chr4	22758519
2469	Chr4	22758564
2470	Chr4	22866160
2471	Chr4	22866149
2472	Chr4	22866125
2473	Chr4	22886660
2474	Chr4	22886587
2475	Chr4	22894489
2476	Chr4	22938350
2477	Chr4	22938351
2478	Chr4	22938430
2479	Chr4	22938649
2480	Chr4	22938067
2481	Chr4	23011472
2482	Chr4	23011469
2483	Chr4	23011450
2484	Chr4	23011448
2485	Chr4	23011118
2486	Chr4	23011108
2487	Chr4	23016485
2488	Chr4	23016823

2489	Chr4	23017441
2490	Chr4	23017438
2491	Chr4	23017408
2492	Chr4	23017403
2493	Chr4	23056528
2494	Chr4	23056556
2495	Chr4	23056033
2496	Chr4	23117059
2497	Chr4	23117022
2498	Chr4	23127723
2499	Chr4	23126879
2500	Chr4	23126866
2501	Chr4	23156276
2502	Chr4	23156323
2503	Chr4	23156332
2504	Chr4	23156666
2505	Chr4	23156668
2506	Chr4	23156681
2507	Chr4	23156690
2508	Chr4	23156162
2509	Chr4	23155839
2510	Chr4	23195525
2511	Chr4	23197357
2512	Chr4	23197308
2513	Chr4	23196915
2514	Chr4	23247308
2515	Chr4	23247624
2516	Chr4	23316056
2517	Chr4	23316116
2518	Chr4	23316340
2519	Chr4	23541483
2520	Chr4	23541500
2521	Chr4	23551001
2522	Chr4	23550981
2523	Chr4	23550902
2524	Chr4	23550662
2525	Chr4	23550644
2526	Chr4	23550566
2527	Chr4	23590843
2528	Chr4	23590859
2529	Chr4	23629071
2530	Chr4	23629038
2531	Chr4	23628869
2532	Chr4	23628821
2533	Chr4	23628813
2534	Chr4	23689727
2535	Chr4	23689718

2536	Chr4	23709443
2537	Chr4	23709496
2538	Chr4	23736174
2539	Chr4	23735859
2540	Chr4	23735523
2541	Chr4	23735496
2542	Chr4	23744483
2543	Chr4	23754608
2544	Chr4	23754894
2545	Chr4	23783656
2546	Chr4	23783741
2547	Chr4	23815841
2548	Chr4	23818285
2549	Chr4	23818345
2550	Chr4	23825476
2551	Chr4	23825658
2552	Chr4	23825671
2553	Chr4	23825751
2554	Chr4	23825369
2555	Chr4	23825361
2556	Chr4	23825360
2557	Chr4	23825346
2558	Chr4	23851338
2559	Chr4	23851302
2560	Chr4	23851006
2561	Chr4	23854822
2562	Chr4	23854800
2563	Chr4	23858092
2564	Chr4	23858057
2565	Chr4	23857779
2566	Chr4	23979898
2567	Chr4	23990344
2568	Chr4	23990235
2569	Chr4	23990182
2570	Chr4	23989921
2571	Chr4	23989896
2572	Chr4	23989892
2573	Chr4	24019930
2574	Chr4	24027430
2575	Chr4	24055635
2576	Chr4	24169077
2577	Chr4	24169121
2578	Chr4	24169378
2579	Chr4	24169438
2580	Chr4	24204847
2581	Chr4	24366434
2582	Chr4	24366446

2583	Chr4	24434179
2584	Chr4	24434167
2585	Chr4	24434102
2586	Chr4	24433939
2587	Chr4	24433914
2588	Chr4	24439828
2589	Chr4	24465310
2590	Chr4	24464939
2591	Chr4	24495560
2592	Chr4	24495514
2593	Chr4	24495512
2594	Chr4	24532719
2595	Chr4	24532732
2596	Chr4	24532779
2597	Chr4	24533163
2598	Chr4	24533176
2599	Chr4	24562526
2600	Chr4	24562710
2601	Chr4	24563622
2602	Chr4	24563639
2603	Chr4	24563653
2604	Chr4	24563659
2605	Chr4	24563672
2606	Chr4	24563995
2607	Chr4	24564005
2608	Chr4	24564024
2609	Chr4	24573311
2610	Chr4	24573338
2611	Chr4	24573348
2612	Chr4	24573396
2613	Chr4	24621314
2614	Chr4	24621032
2615	Chr4	24620995
2616	Chr4	24620970
2617	Chr4	24629086
2618	Chr4	24629121
2619	Chr4	24629407
2620	Chr4	24646025
2621	Chr4	24646042
2622	Chr4	24646090
2623	Chr4	24654657
2624	Chr4	24655022
2625	Chr4	24655065
2626	Chr4	24681558
2627	Chr4	24681506
2628	Chr4	24681248
2629	Chr4	24688484

2630	Chr4	24688428
2631	Chr4	24688412
2632	Chr4	24795543
2633	Chr4	24795914
2634	Chr4	24795950
2635	Chr4	24797099
2636	Chr4	24797171
2637	Chr4	24810270
2638	Chr4	24810237
2639	Chr4	24810225
2640	Chr4	24810206
2641	Chr4	24810200
2642	Chr4	24809883
2643	Chr4	24831518
2644	Chr4	24831852
2645	Chr4	24831853
2646	Chr4	24836955
2647	Chr4	24837053
2648	Chr4	24837378
2649	Chr4	24837390
2650	Chr4	24837408
2651	Chr4	24837410
2652	Chr4	24836901
2653	Chr4	24836900
2654	Chr4	24836893
2655	Chr4	24847996
2656	Chr4	24848387
2657	Chr4	24856878
2658	Chr4	24885296
2659	Chr4	24885313
2660	Chr4	24902707
2661	Chr4	24902713
2662	Chr4	24902731
2663	Chr4	24957446
2664	Chr4	25017807
2665	Chr4	25065429
2666	Chr4	25065696
2667	Chr4	25065475
2668	Chr4	25077589
2669	Chr4	25079923
2670	Chr4	25080177
2671	Chr4	25080210
2672	Chr4	25080229
2673	Chr4	25162688
2674	Chr4	25307812
2675	Chr4	25307800
2676	Chr4	25307757

2677	Chr4	25307735
2678	Chr4	25307717
2679	Chr4	25313883
2680	Chr4	25313489
2681	Chr4	25313469
2682	Chr4	25333596
2683	Chr4	25349089
2684	Chr4	25349031
2685	Chr4	25348741
2686	Chr4	25375367
2687	Chr4	25429222
2688	Chr4	25459135
2689	Chr4	25459166
2690	Chr4	25493653
2691	Chr4	25536514
2692	Chr4	25548252
2693	Chr4	25548171
2694	Chr4	25553221
2695	Chr4	25656742
2696	Chr4	25656771
2697	Chr4	25682985
2698	Chr4	25751640
2699	Chr4	25756826
2700	Chr4	25801132
2701	Chr4	25800862
2702	Chr4	25800823
2703	Chr4	25845887
2704	Chr4	25845868
2705	Chr4	25845490
2706	Chr4	25885890
2707	Chr4	25885880
2708	Chr4	25932093
2709	Chr4	25933078
2710	Chr4	25933455
2711	Chr4	25960441
2712	Chr4	25960733
2713	Chr4	25960802
2714	Chr4	25974079
2715	Chr4	25974884
2716	Chr4	25974905
2717	Chr4	26027754
2718	Chr4	26027793
2719	Chr4	26105654
2720	Chr4	26105956
2721	Chr4	26226491
2722	Chr4	26302226
2723	Chr4	26301919

2724	Chr4	26301917
2725	Chr4	26472740
2726	Chr4	26505425
2727	Chr4	26505690
2728	Chr4	26524759
2729	Chr4	26542620
2730	Chr4	26556750
2731	Chr4	26563370
2732	Chr4	26563367
2733	Chr4	26563350
2734	Chr4	26563347
2735	Chr4	26563334
2736	Chr4	26563326
2737	Chr4	26563322
2738	Chr4	26590359
2739	Chr4	26590306
2740	Chr4	26590302
2741	Chr4	26602361
2742	Chr4	26602378
2743	Chr4	26602737
2744	Chr4	26604791
2745	Chr4	26604786
2746	Chr4	26604419
2747	Chr4	26609490
2748	Chr4	26609388
2749	Chr4	26609059
2750	Chr4	26609021
2751	Chr4	26682982
2752	Chr4	26691707
2753	Chr4	26752962
2754	Chr4	26759570
2755	Chr4	26759602
2756	Chr4	26759620
2757	Chr4	26791107
2758	Chr4	26829715
2759	Chr4	26830015
2760	Chr4	26829623
2761	Chr4	26857907
2762	Chr4	26857519
2763	Chr4	26857248
2764	Chr4	26973271
2765	Chr4	27056657
2766	Chr4	27056628
2767	Chr4	27071031
2768	Chr4	27093427
2769	Chr4	27125111
2770	Chr4	27148802

2771	Chr4	27148500
2772	Chr4	27203076
2773	Chr4	27293271
2774	Chr4	27293088
2775	Chr4	27424061
2776	Chr4	27423766
2777	Chr4	27468180
2778	Chr4	27468202
2779	Chr4	27495413
2780	Chr4	27495751
2781	Chr4	27524122
2782	Chr4	27524386
2783	Chr4	27572539
2784	Chr4	27572419
2785	Chr4	27572413
2786	Chr4	27581375
2787	Chr4	27596064
2788	Chr4	27611957
2789	Chr4	27620858
2790	Chr4	27633154
2791	Chr4	27680673
2792	Chr4	27680674
2793	Chr4	27719915
2794	Chr4	27719690
2795	Chr4	27743691
2796	Chr4	27769377
2797	Chr4	27818906
2798	Chr4	27818868
2799	Chr4	27861670
2800	Chr4	27894802
2801	Chr4	27924735
2802	Chr4	27953718
2803	Chr4	27965508
2804	Chr4	27980820
2805	Chr4	27980495
2806	Chr4	27987771
2807	Chr4	27987749
2808	Chr4	27998934
2809	Chr4	27998843
2810	Chr4	28025938
2811	Chr4	28025942
2812	Chr4	28056159
2813	Chr4	28055951
2814	Chr4	28181822
2815	Chr4	28181818
2816	Chr4	28181807
2817	Chr4	28181790

2818	Chr4	28181448
2819	Chr4	28181445
2820	Chr4	28205379
2821	Chr4	28246017
2822	Chr4	28328507
2823	Chr4	28328509
2824	Chr4	28331776
2825	Chr4	28331785
2826	Chr4	28331403
2827	Chr4	28331363
2828	Chr4	28331024
2829	Chr4	28331020
2830	Chr4	28331003
2831	Chr4	28454579
2832	Chr4	28454155
2833	Chr4	28455619
2834	Chr4	28455567
2835	Chr4	28480874
2836	Chr4	28480876
2837	Chr4	28480887
2838	Chr4	28488345
2839	Chr4	28510638
2840	Chr4	28559093
2841	Chr4	28559376
2842	Chr4	28768338
2843	Chr4	28768400
2844	Chr4	28768402
2845	Chr4	28768050
2846	Chr4	28767998
2847	Chr4	28767974
2848	Chr4	28772361
2849	Chr4	28776170
2850	Chr4	28927892
2851	Chr4	28927902
2852	Chr4	28953601
2853	Chr4	28986687
2854	Chr4	29217186
2855	Chr4	29220183
2856	Chr4	29219781
2857	Chr4	29274282
2858	Chr4	29274246
2859	Chr4	29329131
2860	Chr4	29328468
2861	Chr4	29328449
2862	Chr4	29400758
2863	Chr4	29401027
2864	Chr4	29401034

2865	Chr4	29446780
2866	Chr4	29486959
2867	Chr4	29503173
2868	Chr4	29503145
2869	Chr4	29544530
2870	Chr4	29547873
2871	Chr4	29547816
2872	Chr4	29547515
2873	Chr4	29559629
2874	Chr4	29650518
2875	Chr4	29697009
2876	Chr4	29697008
2877	Chr4	29696694
2878	Chr4	29709115
2879	Chr4	29757377
2880	Chr4	29763122
2881	Chr4	29763162
2882	Chr4	29763337
2883	Chr4	29763362
2884	Chr4	29766758
2885	Chr4	29778337
2886	Chr4	29795684
2887	Chr4	29795785
2888	Chr4	29840570
2889	Chr4	29840849
2890	Chr4	29853319
2891	Chr4	30020152
2892	Chr4	30130749
2893	Chr4	30149156
2894	Chr4	30161904
2895	Chr4	30161254
2896	Chr4	30161251
2897	Chr4	30171973
2898	Chr4	30201467
2899	Chr4	30232161
2900	Chr4	30364179
2901	Chr4	30364494
2902	Chr4	30395619
2903	Chr4	30395908
2904	Chr4	30553253
2905	Chr4	30553203
2906	Chr4	30553179
2907	Chr4	30619457
2908	Chr4	30638927
2909	Chr4	30639085
2910	Chr4	30663431
2911	Chr4	30663711

2912	Chr4	30719682
2913	Chr4	30719400
2914	Chr4	30719359
2915	Chr4	30735106
2916	Chr4	30735128
2917	Chr4	30735189
2918	Chr4	30737933
2919	Chr4	30737912
2920	Chr4	30737911
2921	Chr4	30737626
2922	Chr4	30737593
2923	Chr4	30917684
2924	Chr4	30917670
2925	Chr4	30917669
2926	Chr4	30917309
2927	Chr4	30930642
2928	Chr4	30979692
2929	Chr4	31011537
2930	Chr4	31011799
2931	Chr4	31011833
2932	Chr4	31026606
2933	Chr4	31037261
2934	Chr4	31036807
2935	Chr4	31040081
2936	Chr4	31089938
2937	Chr4	31089641
2938	Chr4	31102420
2939	Chr4	31102397
2940	Chr4	31102081
2941	Chr4	31120004
2942	Chr4	31120008
2943	Chr4	31120399
2944	Chr4	31120404
2945	Chr4	31130029
2946	Chr4	31165919
2947	Chr4	31205659
2948	Chr4	31252979
2949	Chr4	31252781
2950	Chr4	31305695
2951	Chr4	31305710
2952	Chr4	31305715
2953	Chr4	31305979
2954	Chr4	31306019
2955	Chr4	31345863
2956	Chr4	31345834
2957	Chr4	31351413
2958	Chr4	31351703

2959	Chr4	31388890
2960	Chr4	31388894
2961	Chr4	31388932
2962	Chr4	31402312
2963	Chr4	31402345
2964	Chr4	31402573
2965	Chr4	31457192
2966	Chr4	31457207
2967	Chr4	31457231
2968	Chr4	31467919
2969	Chr4	31467934
2970	Chr4	31467973
2971	Chr4	31469893
2972	Chr4	31469582
2973	Chr4	31486669
2974	Chr4	31648365
2975	Chr4	31680972
2976	Chr4	31680635
2977	Chr4	31700390
2978	Chr4	31700734
2979	Chr4	31705425
2980	Chr4	31705804
2981	Chr4	31729953
2982	Chr4	31730361
2983	Chr4	31812949
2984	Chr4	32091060
2985	Chr4	32146951
2986	Chr4	32146806
2987	Chr4	32157600
2988	Chr4	32216071
2989	Chr4	32215995
2990	Chr4	32254210
2991	Chr4	32266929
2992	Chr4	32316138
2993	Chr4	32323930
2994	Chr4	32340888
2995	Chr4	32341170
2996	Chr4	32376239
2997	Chr4	32376697
2998	Chr4	32499277
2999	Chr4	32499285
3000	Chr4	32569087
3001	Chr4	32590818
3002	Chr4	32638771
3003	Chr4	32664619
3004	Chr4	32664875
3005	Chr4	32664884

3006	Chr4	32664963
3007	Chr4	32706519
3008	Chr4	32706823
3009	Chr4	32706848
3010	Chr4	32749821
3011	Chr4	32829327
3012	Chr4	32829338
3013	Chr4	32829372
3014	Chr4	32912614
3015	Chr4	32912816
3016	Chr4	32915424
3017	Chr4	32965838
3018	Chr4	32969944
3019	Chr4	32970149
3020	Chr4	33082489
3021	Chr4	33115117
3022	Chr4	33115054
3023	Chr4	33115049
3024	Chr4	33138676
3025	Chr4	33138642
3026	Chr4	33138437
3027	Chr4	33191477
3028	Chr4	33191480
3029	Chr4	33191842
3030	Chr4	33239085
3031	Chr4	33239369
3032	Chr4	33239414
3033	Chr4	33244316
3034	Chr4	33284897
3035	Chr4	33284900
3036	Chr4	33347496
3037	Chr4	33375931
3038	Chr4	33375256
3039	Chr4	33418121
3040	Chr4	33418150
3041	Chr4	33418400
3042	Chr4	33449718
3043	Chr4	33499565
3044	Chr4	33514545
3045	Chr4	33541087
3046	Chr4	33715166
3047	Chr4	33720082
3048	Chr4	33720312
3049	Chr4	33720353
3050	Chr4	33738437
3051	Chr4	33760912
3052	Chr4	33793177

3053	Chr4	33793437
3054	Chr4	33822823
3055	Chr4	33833851
3056	Chr4	33884547
3057	Chr4	33884641
3058	Chr4	33884882
3059	Chr4	33981339
3060	Chr4	34074358
3061	Chr4	34075207
3062	Chr4	34075225
3063	Chr4	34130863
3064	Chr4	34131182
3065	Chr4	34177199
3066	Chr4	34186672
3067	Chr4	34258423
3068	Chr4	34258406
3069	Chr4	34258380
3070	Chr4	34266818
3071	Chr4	34266843
3072	Chr4	34280239
3073	Chr4	34302143
3074	Chr4	34302120
3075	Chr4	34395858
3076	Chr4	34420594
3077	Chr4	34546207
3078	Chr4	34578149
3079	Chr4	34588664
3080	Chr4	34590093
3081	Chr4	34686530
3082	Chr4	34785705
3083	Chr4	34805212
3084	Chr4	34805170
3085	Chr4	34805139
3086	Chr4	34860008
3087	Chr4	34860006
3088	Chr4	34883822
3089	Chr4	34913130
3090	Chr4	34913140
3091	Chr4	34913173
3092	Chr4	34924297
3093	Chr4	34928822
3094	Chr4	34928839
3095	Chr4	34934520
3096	Chr4	34965494
3097	Chr4	34965458
3098	Chr4	34974848
3099	Chr4	34974846

3100	Chr4	34974572
3101	Chr4	34987266
3102	Chr4	35044280
3103	Chr4	35044260
3104	Chr4	35044034
3105	Chr4	35072170
3106	Chr4	35072240
3107	Chr4	35072545
3108	Chr4	35072597
3109	Chr4	35072912
3110	Chr4	35072847
3111	Chr4	35081990
3112	Chr4	35081970
3113	Chr4	35081673
3114	Chr4	35081648
3115	Chr4	35087882
3116	Chr4	35088205
3117	Chr4	35088217
3118	Chr4	35234415
3119	Chr4	35234395
3120	Chr4	35266736
3121	Chr4	35300543
3122	Chr4	35304841
3123	Chr4	35328588
3124	Chr4	35328040
3125	Chr4	35401423
3126	Chr4	35401478
3127	Chr4	35612833
3128	Chr4	35680323
3129	Chr4	35694488
3130	Chr4	35694852
3131	Chr4	35694057
3132	Chr4	35743771
3133	Chr4	35743822
3134	Chr4	35752020
3135	Chr4	35790492
3136	Chr4	35790127
3137	Chr4	35790121
3138	Chr4	35789749
3139	Chr4	35827230
3140	Chr4	35868959
3141	Chr4	35869335
3142	Chr4	36113030
3143	Chr4	36112989
3144	Chr4	36112955
3145	Chr4	36112738
3146	Chr4	36126181

3147	Chr4	36130753
3148	Chr4	36199275
3149	Chr4	36202565
3150	Chr4	36202887
3151	Chr4	36202913
3152	Chr4	36202454
3153	Chr4	36202225
3154	Chr4	36202175
3155	Chr4	36215027
3156	Chr4	36262356
3157	Chr4	36262335
3158	Chr4	36262008
3159	Chr4	36261924
3160	Chr4	36270675
3161	Chr4	36270651
3162	Chr4	36270585
3163	Chr4	36350641
3164	Chr4	36362090
3165	Chr4	36445119
3166	Chr4	36445381
3167	Chr4	36488032
3168	Chr4	36493375
3169	Chr4	36513666
3170	Chr4	36513732
3171	Chr4	36586802
3172	Chr4	36586792
3173	Chr4	36586747
3174	Chr4	36614556
3175	Chr4	36614551
3176	Chr4	36614350
3177	Chr4	36614333
3178	Chr4	36634763
3179	Chr4	36817267
3180	Chr4	36816913
3181	Chr4	36816912
3182	Chr4	36816863
3183	Chr4	36816834
3184	Chr4	36816827
3185	Chr4	36859623
3186	Chr4	36859187
3187	Chr4	36863241
3188	Chr4	37047842
3189	Chr4	37047507
3190	Chr4	37047506
3191	Chr4	37062404
3192	Chr4	37062798
3193	Chr4	37098279

3194	Chr4	37098358
3195	Chr4	37098930
3196	Chr4	37107564
3197	Chr4	37107592
3198	Chr4	37183822
3199	Chr4	37183862
3200	Chr4	37183864
3201	Chr4	37215703
3202	Chr4	37215721
3203	Chr4	37215125
3204	Chr4	37217447
3205	Chr4	37217454
3206	Chr4	37259882
3207	Chr4	37259535
3208	Chr4	37269170
3209	Chr4	37268782
3210	Chr4	37268705
3211	Chr4	37295157
3212	Chr4	37303999
3213	Chr4	37319967
3214	Chr4	37362015
3215	Chr4	37455067
3216	Chr4	37455085
3217	Chr4	37463513
3218	Chr4	37463883
3219	Chr4	37464855
3220	Chr4	37465203
3221	Chr4	37465259
3222	Chr4	37465287
3223	Chr4	37557630
3224	Chr4	37558007
3225	Chr4	37609758
3226	Chr4	37611969
3227	Chr4	37651217
3228	Chr4	37650831
3229	Chr4	37658336
3230	Chr4	37658313
3231	Chr4	37657966
3232	Chr4	37657949
3233	Chr4	37663028
3234	Chr4	37662974
3235	Chr4	37673134
3236	Chr4	37678015
3237	Chr4	37677971
3238	Chr4	37677968
3239	Chr4	37677935
3240	Chr4	37677737

3241	Chr4	37681773
3242	Chr4	37681753
3243	Chr4	37681732
3244	Chr4	37712842
3245	Chr4	37722052
3246	Chr4	37722518
3247	Chr4	37750603
3248	Chr4	37750615
3249	Chr4	37774019
3250	Chr4	37839513
3251	Chr4	37839545
3252	Chr4	37839546
3253	Chr4	37955777
3254	Chr4	37955406
3255	Chr4	37975987
3256	Chr4	37997689
3257	Chr4	38023556
3258	Chr4	38023237
3259	Chr4	38023234
3260	Chr4	38034147
3261	Chr4	38080578
3262	Chr4	38125606
3263	Chr4	38125622
3264	Chr4	38232198
3265	Chr4	38232256
3266	Chr4	38245391
3267	Chr4	38254357
3268	Chr4	38303045
3269	Chr4	38351913
3270	Chr4	38372565
3271	Chr4	38372506
3272	Chr4	38449476
3273	Chr4	38449767
3274	Chr4	38449777
3275	Chr4	38464147
3276	Chr4	38475662
3277	Chr4	38482097
3278	Chr4	38522059
3279	Chr4	38586172
3280	Chr4	38586206
3281	Chr4	38586258
3282	Chr4	38603376
3283	Chr4	38603108
3284	Chr4	38603096
3285	Chr4	38604515
3286	Chr4	38604445
3287	Chr4	38616717

3288	Chr4	38617716
3289	Chr4	38687555
3290	Chr4	38761941
3291	Chr4	38794938
3292	Chr4	38852914
3293	Chr4	38852595
3294	Chr4	38869920
3295	Chr4	38955336
3296	Chr4	38955751
3297	Chr4	38960048
3298	Chr4	39015728
3299	Chr4	39015740
3300	Chr4	39030052
3301	Chr4	39049480
3302	Chr4	39049425
3303	Chr4	39059089
3304	Chr4	39079956
3305	Chr4	39079931
3306	Chr4	39159651
3307	Chr4	39164536
3308	Chr4	39164535
3309	Chr4	39191025
3310	Chr4	39205721
3311	Chr4	39222666
3312	Chr4	39222844
3313	Chr4	39245731
3314	Chr4	39256385
3315	Chr4	39256375
3316	Chr4	39380917
3317	Chr4	39380902
3318	Chr4	39396301
3319	Chr4	39446137
3320	Chr4	39482078
3321	Chr4	39634167
3322	Chr4	39646434
3323	Chr4	39646374
3324	Chr4	39675004
3325	Chr4	39674990
3326	Chr4	39674756
3327	Chr4	39674753
3328	Chr4	39881416
3329	Chr4	39890355
3330	Chr4	39889640
3331	Chr4	39931388
3332	Chr4	40028773
3333	Chr4	40110956
3334	Chr4	40111127

3335	Chr4	40111134
3336	Chr4	40216289
3337	Chr4	40226419
3338	Chr4	40293569
3339	Chr4	40323062
3340	Chr4	40358355
3341	Chr4	40358709
3342	Chr4	40382656
3343	Chr4	40382746
3344	Chr4	40384637
3345	Chr4	40384642
3346	Chr4	40384942
3347	Chr4	40393846
3348	Chr4	40393568
3349	Chr4	40416210
3350	Chr4	40421805
3351	Chr4	40421501
3352	Chr4	40467082
3353	Chr4	40471511
3354	Chr4	40471502
3355	Chr4	40513866
3356	Chr4	40517594
3357	Chr4	40517977
3358	Chr4	40517482
3359	Chr4	40517255
3360	Chr4	40517209
3361	Chr4	40546048
3362	Chr4	40546041
3363	Chr4	40545741
3364	Chr4	40552384
3365	Chr4	40552368
3366	Chr4	40552350
3367	Chr4	40575405
3368	Chr4	40594533
3369	Chr4	40594523
3370	Chr4	40594257
3371	Chr4	40616818
3372	Chr4	40616836
3373	Chr4	40668959
3374	Chr4	40688412
3375	Chr4	40688451
3376	Chr4	40688471
3377	Chr4	40688656
3378	Chr4	40694121
3379	Chr4	40727508
3380	Chr4	40727943
3381	Chr4	40728178

3382	Chr4	40805366
3383	Chr4	40805571
3384	Chr4	40805572
3385	Chr4	40805605
3386	Chr4	40805621
3387	Chr4	40805622
3388	Chr4	40805628
3389	Chr4	40805666
3390	Chr4	40823325
3391	Chr4	40823322
3392	Chr4	40846122
3393	Chr4	40846158
3394	Chr4	40850844
3395	Chr4	40899564
3396	Chr4	40985732
3397	Chr4	40985776
3398	Chr4	40985409
3399	Chr4	40985218
3400	Chr4	41013381
3401	Chr4	41013044
3402	Chr4	41028355
3403	Chr4	41073039
3404	Chr4	41073021
3405	Chr4	41082098
3406	Chr4	41082252
3407	Chr4	41082259
3408	Chr4	41360628
3409	Chr4	41383407
3410	Chr4	41383180
3411	Chr4	41389850
3412	Chr4	41418978
3413	Chr4	41421728
3414	Chr4	41505477
3415	Chr4	41505704
3416	Chr4	41513451
3417	Chr4	41513281
3418	Chr4	41605107
3419	Chr4	41605087
3420	Chr4	41659502
3421	Chr4	41659558
3422	Chr4	41683667
3423	Chr4	41684261
3424	Chr4	41689202
3425	Chr4	41690698
3426	Chr4	41690632
3427	Chr4	41695075
3428	Chr4	41764598

3429	Chr4	42010540
3430	Chr4	42010475
3431	Chr4	42221189
3432	Chr4	42301421
3433	Chr4	42311004
3434	Chr4	42311029
3435	Chr4	42330385
3436	Chr4	42330333
3437	Chr4	42355559
3438	Chr4	42355576
3439	Chr4	42354840
3440	Chr4	42597668
3441	Chr4	42630621
3442	Chr4	42631011
3443	Chr4	42658410
3444	Chr4	42722351
3445	Chr4	42722375
3446	Chr4	42721897
3447	Chr4	42834871
3448	Chr4	42932756
3449	Chr4	42932698
3450	Chr4	42957905
3451	Chr4	43015359
3452	Chr4	43015745
3453	Chr4	43029673
3454	Chr4	43074868
3455	Chr4	43074721
3456	Chr4	43074676
3457	Chr4	43095539
3458	Chr4	43114154
3459	Chr4	43119496
3460	Chr4	43119185
3461	Chr4	43156239
3462	Chr4	43161941
3463	Chr4	43161646
3464	Chr4	43185237
3465	Chr4	43206110
3466	Chr4	43212732
3467	Chr4	43220559
3468	Chr4	43290366
3469	Chr4	43295472
3470	Chr4	43386915
3471	Chr4	43483783
3472	Chr4	43522293
3473	Chr4	43522306
3474	Chr4	43555092
3475	Chr4	43555273

3476	Chr4	43624521
3477	Chr4	43651367
3478	Chr4	43657255
3479	Chr4	43728619
3480	Chr4	43799425
3481	Chr4	43826728
3482	Chr4	43854424
3483	Chr4	43854433
3484	Chr4	43854501
3485	Chr4	43854743
3486	Chr4	43854829
3487	Chr4	43854123
3488	Chr4	43886383
3489	Chr4	43886360
3490	Chr4	43885951
3491	Chr4	43885925
3492	Chr4	43891626
3493	Chr4	43891274
3494	Chr4	43891224
3495	Chr4	43946141
3496	Chr4	43969089
3497	Chr4	44132460
3498	Chr4	44132130
3499	Chr4	44144692
3500	Chr4	44145067
3501	Chr4	44181480
3502	Chr4	44181482
3503	Chr4	44181499
3504	Chr4	44181508
3505	Chr4	44180974
3506	Chr4	44227744
3507	Chr4	44307681
3508	Chr4	44307648
3509	Chr4	44311253
3510	Chr4	44310827
3511	Chr4	44337965
3512	Chr4	44337639
3513	Chr4	44374220
3514	Chr4	44374216
3515	Chr4	44429355
3516	Chr4	44429663
3517	Chr4	44429669
3518	Chr4	44429280
3519	Chr4	44429241
3520	Chr4	44428965
3521	Chr4	44428875
3522	Chr4	44445790

3523	Chr4	44494633
3524	Chr4	44540336
3525	Chr4	44540304
3526	Chr4	44551261
3527	Chr4	44572388
3528	Chr4	44572354
3529	Chr4	44669392
3530	Chr4	44684725
3531	Chr4	44684056
3532	Chr4	44707796
3533	Chr4	44707816
3534	Chr4	44708076
3535	Chr4	44708101
3536	Chr4	44735675
3537	Chr4	44764735
3538	Chr4	44763921
3539	Chr4	44765912
3540	Chr4	44787354
3541	Chr4	44819106
3542	Chr4	44838149
3543	Chr4	44838090
3544	Chr4	44883946
3545	Chr4	44883723
3546	Chr4	44883717
3547	Chr4	45025117
3548	Chr4	45025105
3549	Chr4	45093103
3550	Chr4	45093093
3551	Chr4	45219320
3552	Chr4	45259065
3553	Chr4	45259072
3554	Chr4	45271203
3555	Chr4	45312907
3556	Chr4	45456704
3557	Chr4	45456701
3558	Chr4	45617682
3559	Chr4	45654076
3560	Chr4	45653950
3561	Chr4	45653888
3562	Chr4	45673066
3563	Chr4	45673079
3564	Chr4	45911225
3565	Chr4	45911444
3566	Chr4	45979115
3567	Chr4	45979141
3568	Chr4	45979033
3569	Chr4	45978796

3570	Chr4	45978783
3571	Chr4	45978772
3572	Chr4	45986137
3573	Chr4	46050217
3574	Chr4	46050221
3575	Chr4	46050235
3576	Chr4	46049869
3577	Chr4	46049857
3578	Chr4	46049823
3579	Chr4	46049464
3580	Chr4	46049430
3581	Chr4	46099660
3582	Chr4	46151412
3583	Chr4	46151499
3584	Chr4	46164881
3585	Chr4	46165114
3586	Chr4	46164718
3587	Chr4	46205299
3588	Chr4	46205290
3589	Chr4	46205281
3590	Chr4	46204850
3591	Chr4	46295958
3592	Chr4	46349588
3593	Chr4	46350702
3594	Chr4	46390587
3595	Chr4	46401893
3596	Chr4	46401927
3597	Chr4	46401945
3598	Chr4	46401946
3599	Chr4	46402200
3600	Chr4	46417130
3601	Chr4	46417182
3602	Chr4	46421342
3603	Chr4	46421054
3604	Chr4	46421027
3605	Chr4	46459149
3606	Chr4	46459152
3607	Chr4	46459180
3608	Chr4	46526653
3609	Chr4	46527803
3610	Chr4	46527355
3611	Chr4	46526997
3612	Chr4	46526971
3613	Chr4	46586322
3614	Chr4	46586290
3615	Chr4	46586235
3616	Chr4	46586012

3617	Chr4	46609957
3618	Chr4	46626881
3619	Chr4	46626927
3620	Chr4	46633518
3621	Chr4	46634675
3622	Chr4	46634699
3623	Chr4	46634964
3624	Chr4	46640008
3625	Chr4	46642181
3626	Chr4	46642268
3627	Chr4	46649611
3628	Chr4	46649707
3629	Chr4	46724924
3630	Chr4	46724938
3631	Chr4	46814159
3632	Chr4	46814221
3633	Chr4	46814354
3634	Chr4	46814365
3635	Chr4	46814407
3636	Chr4	46835569
3637	Chr4	46898984
3638	Chr4	46898927
3639	Chr4	46898909
3640	Chr4	46898688
3641	Chr4	46942199
3642	Chr4	46942225
3643	Chr4	46942226
3644	Chr4	46952547
3645	Chr4	46952588
3646	Chr4	46975988
3647	Chr4	46975991
3648	Chr4	46976192
3649	Chr4	46976193
3650	Chr4	46976247
3651	Chr4	47022798
3652	Chr4	47022797
3653	Chr4	47022766
3654	Chr4	47022552
3655	Chr4	47022531
3656	Chr4	47119764
3657	Chr4	47122464
3658	Chr4	47122264
3659	Chr4	47122217
3660	Chr4	47122198
3661	Chr4	47124080
3662	Chr4	47138877
3663	Chr4	47139182

3664	Chr4	47195751
3665	Chr4	47195953
3666	Chr4	47195295
3667	Chr4	47195267
3668	Chr4	47195252
3669	Chr4	47207313
3670	Chr4	47254577
3671	Chr4	47254575
3672	Chr4	47254519
3673	Chr4	47254504
3674	Chr4	47325480
3675	Chr4	47406810
3676	Chr4	47406779
3677	Chr4	47468561
3678	Chr4	47468564
3679	Chr4	47558454
3680	Chr4	47621386
3681	Chr4	47693337
3682	Chr4	47810122
3683	Chr4	47873738
3684	Chr4	47873777
3685	Chr4	47874098
3686	Chr4	47874111
3687	Chr4	47901155
3688	Chr4	47901169
3689	Chr4	47901489
3690	Chr4	47901506
3691	Chr4	47901555
3692	Chr4	47929798
3693	Chr4	47930142
3694	Chr4	47929719
3695	Chr4	47929718
3696	Chr4	47929391
3697	Chr4	47929381
3698	Chr4	48024449
3699	Chr4	48026450
3700	Chr4	48026683
3701	Chr4	48026709
3702	Chr4	48026303
3703	Chr4	48026286
3704	Chr4	48025993
3705	Chr4	48035148
3706	Chr4	48035090
3707	Chr4	48051617
3708	Chr4	48051595
3709	Chr4	48069852
3710	Chr4	48069818

3711	Chr4	48069548
3712	Chr4	48069500
3713	Chr4	48069453
3714	Chr4	48095530
3715	Chr4	48104995
3716	Chr4	48104951
3717	Chr4	48104927
3718	Chr4	48104592
3719	Chr4	48162590
3720	Chr4	48162267
3721	Chr4	48213540
3722	Chr4	48213516
3723	Chr4	48223194
3724	Chr4	48276925
3725	Chr4	48287909
3726	Chr4	48308554
3727	Chr4	48308553
3728	Chr4	48308532
3729	Chr4	48308191
3730	Chr4	48308172
3731	Chr4	48308171
3732	Chr4	48359226
3733	Chr4	48371687
3734	Chr4	48371693
3735	Chr4	48371704
3736	Chr4	48371978
3737	Chr4	48372008
3738	Chr4	48391604
3739	Chr4	48391643
3740	Chr4	48391868
3741	Chr4	48422331
3742	Chr4	48422025
3743	Chr4	48501862
3744	Chr4	48501870
3745	Chr4	48501899
3746	Chr4	48501906
3747	Chr4	48502234
3748	Chr4	48502293
3749	Chr4	48502307
3750	Chr4	48502317
3751	Chr4	48501799
3752	Chr4	48501758
3753	Chr4	48501415
3754	Chr4	48548536
3755	Chr4	48548388
3756	Chr4	48548385
3757	Chr4	48548328

3758	Chr4	48561695
3759	Chr4	48561729
3760	Chr4	48561767
3761	Chr4	48603852
3762	Chr4	48603607
3763	Chr4	48603578
3764	Chr4	48694104
3765	Chr4	48694082
3766	Chr4	48694069
3767	Chr4	48694053
3768	Chr4	48693685
3769	Chr4	48711833
3770	Chr4	48711806
3771	Chr4	48711504
3772	Chr4	48711497
3773	Chr4	48711471
3774	Chr4	48777314
3775	Chr4	48777376
3776	Chr4	48809665
3777	Chr4	48811041
3778	Chr4	48811013
3779	Chr4	48810817
3780	Chr4	48810765
3781	Chr4	48856926
3782	Chr4	48856927
3783	Chr4	48857171
3784	Chr4	48955800
3785	Chr4	48975405
3786	Chr4	48975382
3787	Chr4	48975368
3788	Chr4	48975117
3789	Chr4	48975054
3790	Chr4	48980609
3791	Chr4	48980665
3792	Chr4	48982434
3793	Chr4	48982728
3794	Chr4	48982735
3795	Chr4	48982737
3796	Chr4	48982800
3797	Chr4	48982802
3798	Chr4	49017697
3799	Chr4	49041721
3800	Chr4	49041717
3801	Chr4	49041715
3802	Chr4	49041666
3803	Chr4	49131061
3804	Chr4	49131404

3805	Chr4	49131612
3806	Chr4	49183810
3807	Chr4	49214869
3808	Chr4	49261396
3809	Chr4	49278734
3810	Chr4	49297448
3811	Chr4	49297173
3812	Chr4	49382364
3813	Chr4	49382383
3814	Chr4	49390041
3815	Chr4	49472307
3816	Chr4	49506578
3817	Chr4	49506567
3818	Chr4	49506553
3819	Chr4	49506385
3820	Chr4	49506314
3821	Chr4	49529139
3822	Chr4	49576716
3823	Chr4	49576715
3824	Chr4	49579662
3825	Chr4	49580084
3826	Chr4	49624918
3827	Chr4	49625231
3828	Chr4	49642594
3829	Chr4	49648733
3830	Chr4	49648945
3831	Chr4	49656892
3832	Chr4	49656890
3833	Chr4	49677715
3834	Chr4	49681152
3835	Chr4	49746423
3836	Chr4	49746117
3837	Chr4	49766763
3838	Chr4	49789368
3839	Chr4	49922090
3840	Chr4	49922097
3841	Chr4	50135078
3842	Chr4	50164968
3843	Chr4	50165345
3844	Chr4	50175259
3845	Chr4	50175062
3846	Chr4	50245599
3847	Chr4	50295303
3848	Chr4	50303902
3849	Chr4	50429227
3850	Chr4	50446609
3851	Chr4	50446918

3852	Chr4	50541883
3853	Chr4	50573120
3854	Chr4	50573357
3855	Chr4	50579883
3856	Chr4	50579811
3857	Chr4	50759057
3858	Chr4	50759089
3859	Chr4	50764086
3860	Chr4	50788121
3861	Chr4	51449139
3862	Chr4	52109929
3863	Chr4	60635088
3864	Chr4	60745558
3865	Chr4	60768696
3866	Chr4	60768621
3867	Chr4	60822964
3868	Chr4	60897885
3869	Chr4	60930383
3870	Chr4	60932762
3871	Chr4	60935019
3872	Chr4	60934995
3873	Chr4	60937153
3874	Chr4	61010967
3875	Chr4	61039163
3876	Chr4	61054639
3877	Chr4	61071744
3878	Chr4	61071467
3879	Chr4	62993832
3880	Chr4	63012069
3881	Chr4	63137292
3882	Chr4	63306820
3883	Chr4	63306771
3884	Chr4	63332993
3885	Chr4	63902161
3886	Chr4	64172502
3887	Chr4	64239118
3888	Chr4	64297095
3889	Chr4	64563526
3890	Chr4	64690281
3891	Chr4	64808407
3892	Chr4	64808378
3893	Chr4	64808369
3894	Chr4	64808219
3895	Chr4	64808217
3896	Chr4	64856191
3897	Chr4	64899984
3898	Chr4	64899702

3899	Chr4	64899691
3900	Chr4	64953140
3901	Chr4	64979320
3902	Chr4	64979270
3903	Chr4	64978976
3904	Chr4	64999575
3905	Chr4	65014111
3906	Chr4	65024050
3907	Chr4	65046829
3908	Chr4	65046871
3909	Chr4	65106970
3910	Chr4	65107344
3911	Chr4	66051037
3912	Chr4	66224315
3913	Chr5	3544001
3914	Chr5	3544002
3915	Chr5	3544299
3916	Chr5	3739893
3917	Chr5	3739859
3918	Chr5	3739608
3919	Chr5	3760617
3920	Chr5	3760620
3921	Chr5	3847958
3922	Chr5	3847709
3923	Chr5	3847684
3924	Chr5	3983751
3925	Chr5	3983426
3926	Chr5	3988418
3927	Chr5	4029591
3928	Chr5	4029258
3929	Chr5	4032637
3930	Chr5	4032607
3931	Chr5	4077844
3932	Chr5	4092536
3933	Chr5	4104036
3934	Chr5	4104078
3935	Chr5	4149138
3936	Chr5	4195320
3937	Chr5	4195611
3938	Chr5	4195641
3939	Chr5	4195646
3940	Chr5	4242084
3941	Chr5	4242361
3942	Chr5	4242414
3943	Chr5	5657108
3944	Chr5	6914293
3945	Chr5	6914308

3946	Chr5	6914328
3947	Chr5	6914644
3948	Chr5	6914730
3949	Chr5	8187329
3950	Chr5	8776330
3951	Chr5	8776309
3952	Chr5	8776292
3953	Chr5	8776277
3954	Chr5	8776072
3955	Chr5	8776056
3956	Chr5	8776036
3957	Chr5	8775999
3958	Chr5	8775992
3959	Chr5	8775985
3960	Chr5	8775984
3961	Chr5	9072027
3962	Chr5	9838003
3963	Chr5	9864868
3964	Chr5	10261686
3965	Chr5	10393005
3966	Chr5	10723110
3967	Chr5	10932198
3968	Chr5	11025830
3969	Chr5	11293883
3970	Chr5	11552186
3971	Chr5	11988283
3972	Chr5	11988303
3973	Chr5	11988318
3974	Chr5	12190103
3975	Chr5	12733175
3976	Chr5	12733174
3977	Chr5	12733160
3978	Chr5	12732873
3979	Chr5	12732841
3980	Chr5	12795063
3981	Chr5	12795007
3982	Chr5	12794743
3983	Chr5	12794716
3984	Chr5	12794703
3985	Chr5	13180469
3986	Chr5	13180483
3987	Chr5	13451984
3988	Chr5	13452323
3989	Chr5	13452354
3990	Chr5	13479092
3991	Chr5	13951275
3992	Chr5	14065563

3993	Chr5	14158551
3994	Chr5	14230796
3995	Chr5	14291239
3996	Chr5	14842271
3997	Chr5	14947783
3998	Chr5	14947781
3999	Chr5	15102358
4000	Chr5	15679275
4001	Chr5	15735723
4002	Chr5	15743554
4003	Chr5	15918064
4004	Chr5	15918029
4005	Chr5	16105546
4006	Chr5	16342020
4007	Chr5	16342058
4008	Chr5	16342269
4009	Chr5	16342275
4010	Chr5	16342280
4011	Chr5	16342291
4012	Chr5	16615381
4013	Chr5	16915799
4014	Chr5	16962755
4015	Chr5	16962752
4016	Chr5	16962749
4017	Chr5	16962722
4018	Chr5	16962718
4019	Chr5	16962716
4020	Chr5	16962709
4021	Chr5	17207794
4022	Chr5	17383575
4023	Chr5	17383746
4024	Chr5	17395819
4025	Chr5	17411036
4026	Chr5	17411084
4027	Chr5	17411088
4028	Chr5	17411098
4029	Chr5	17411491
4030	Chr5	17416041
4031	Chr5	17416069
4032	Chr5	17416418
4033	Chr5	17416422
4034	Chr5	17449654
4035	Chr5	17449624
4036	Chr5	17449613
4037	Chr5	17452620
4038	Chr5	17452642
4039	Chr5	17452674

4040	Chr5	17452892
4041	Chr5	17452896
4042	Chr5	17452904
4043	Chr5	17452917
4044	Chr5	17452930
4045	Chr5	17452957
4046	Chr5	17454679
4047	Chr5	17454382
4048	Chr5	17454361
4049	Chr5	18308704
4050	Chr5	19236087
4051	Chr5	19315909
4052	Chr5	19315967
4053	Chr5	19316279
4054	Chr5	19375029
4055	Chr5	19374765
4056	Chr5	19394895
4057	Chr5	19447624
4058	Chr5	19447160
4059	Chr5	19447096
4060	Chr5	19447088
4061	Chr5	19446895
4062	Chr5	19537360
4063	Chr5	19537538
4064	Chr5	19607935
4065	Chr5	19713713
4066	Chr5	19713740
4067	Chr5	19861006
4068	Chr5	19860647
4069	Chr5	19860645
4070	Chr5	19860619
4071	Chr5	19860611
4072	Chr5	19999121
4073	Chr5	20061561
4074	Chr5	20752256
4075	Chr5	21365841
4076	Chr5	21365819
4077	Chr5	21365444
4078	Chr5	21365403
4079	Chr5	21365396
4080	Chr5	21700269
4081	Chr5	22127235
4082	Chr5	22669368
4083	Chr5	22670265
4084	Chr5	22669884
4085	Chr5	22693750
4086	Chr5	22741648

4087	Chr5	22741629
4088	Chr5	22741592
4089	Chr5	22741578
4090	Chr5	22753948
4091	Chr5	22826173
4092	Chr5	22826509
4093	Chr5	22826516
4094	Chr5	22924941
4095	Chr5	22924965
4096	Chr5	22924878
4097	Chr5	22924877
4098	Chr5	22924836
4099	Chr5	22924833
4100	Chr5	22924828
4101	Chr5	22924811
4102	Chr5	22924573
4103	Chr5	22929268
4104	Chr5	23017155
4105	Chr5	23016911
4106	Chr5	23016818
4107	Chr5	23020814
4108	Chr5	23020810
4109	Chr5	23206743
4110	Chr5	23206716
4111	Chr5	23206661
4112	Chr5	23206475
4113	Chr5	23298691
4114	Chr5	23298699
4115	Chr5	23298932
4116	Chr5	23298942
4117	Chr5	23303651
4118	Chr5	23303650
4119	Chr5	23324868
4120	Chr5	23513365
4121	Chr5	23694020
4122	Chr5	23744086
4123	Chr5	23764798
4124	Chr5	23764807
4125	Chr5	23780608
4126	Chr5	23780898
4127	Chr5	23780911
4128	Chr5	23794296
4129	Chr5	23800057
4130	Chr5	23800083
4131	Chr5	23808249
4132	Chr5	23808580
4133	Chr5	23808599

4134	Chr5	23867654
4135	Chr5	23981314
4136	Chr5	23981334
4137	Chr5	23981356
4138	Chr5	23981381
4139	Chr5	24035629
4140	Chr5	24053345
4141	Chr5	24057793
4142	Chr5	24059174
4143	Chr5	24058803
4144	Chr5	24070013
4145	Chr5	24101402
4146	Chr5	24101413
4147	Chr5	24101420
4148	Chr5	24101435
4149	Chr5	24101471
4150	Chr5	24101746
4151	Chr5	24116224
4152	Chr5	24115866
4153	Chr5	24115837
4154	Chr5	24115825
4155	Chr5	24116962
4156	Chr5	24116967
4157	Chr5	24116968
4158	Chr5	24116903
4159	Chr5	24116467
4160	Chr5	24116463
4161	Chr5	24208603
4162	Chr5	24237393
4163	Chr5	24237466
4164	Chr5	24237306
4165	Chr5	24248414
4166	Chr5	24248342
4167	Chr5	24248150
4168	Chr5	24248105
4169	Chr5	24251391
4170	Chr5	24251390
4171	Chr5	24251358
4172	Chr5	24259490
4173	Chr5	24259700
4174	Chr5	24259729
4175	Chr5	24268836
4176	Chr5	24356549
4177	Chr5	24356293
4178	Chr5	24356243
4179	Chr5	24372113
4180	Chr5	24371394

4181	Chr5	24371340
4182	Chr5	24498080
4183	Chr5	24497796
4184	Chr5	24497714
4185	Chr5	24509149
4186	Chr5	24535025
4187	Chr5	24536620
4188	Chr5	24627056
4189	Chr5	24627037
4190	Chr5	24627027
4191	Chr5	24653041
4192	Chr5	24653047
4193	Chr5	24739149
4194	Chr5	24777075
4195	Chr5	24776802
4196	Chr5	24785762
4197	Chr5	24785472
4198	Chr5	24785430
4199	Chr5	24785402
4200	Chr5	24789874
4201	Chr5	24789889
4202	Chr5	24789932
4203	Chr5	24789939
4204	Chr5	24789967
4205	Chr5	24828054
4206	Chr5	24828088
4207	Chr5	24828136
4208	Chr5	24828398
4209	Chr5	24828417
4210	Chr5	24828434
4211	Chr5	24865307
4212	Chr5	24865332
4213	Chr5	24865346
4214	Chr5	24865617
4215	Chr5	24909409
4216	Chr5	24909410
4217	Chr5	24909449
4218	Chr5	24909693
4219	Chr5	24909703
4220	Chr5	24920119
4221	Chr5	24919952
4222	Chr5	24952837
4223	Chr5	24952850
4224	Chr5	24952852
4225	Chr5	24995157
4226	Chr5	25053701
4227	Chr5	25054097

4228	Chr5	25133956
4229	Chr5	25133571
4230	Chr5	25158033
4231	Chr5	25158464
4232	Chr5	25250446
4233	Chr5	25250502
4234	Chr5	25250802
4235	Chr5	25250824
4236	Chr5	25250825
4237	Chr5	25250844
4238	Chr5	25281076
4239	Chr5	25281125
4240	Chr5	25281421
4241	Chr5	25280774
4242	Chr5	25345824
4243	Chr5	25345829
4244	Chr5	25345986
4245	Chr5	25346018
4246	Chr5	25346069
4247	Chr5	25409529
4248	Chr5	25409585
4249	Chr5	25409605
4250	Chr5	25409627
4251	Chr5	25447656
4252	Chr5	25457879
4253	Chr5	25458295
4254	Chr5	25457770
4255	Chr5	25457514
4256	Chr5	25457445
4257	Chr5	25490194
4258	Chr5	25490168
4259	Chr5	25490115
4260	Chr5	25534528
4261	Chr5	25534973
4262	Chr5	25534985
4263	Chr5	25573375
4264	Chr5	25573310
4265	Chr5	25573291
4266	Chr5	25573092
4267	Chr5	25573086
4268	Chr5	25656351
4269	Chr5	25656711
4270	Chr5	25656770
4271	Chr5	25673838
4272	Chr5	25673795
4273	Chr5	25673761
4274	Chr5	25678858

4275	Chr5	25679115
4276	Chr5	25679130
4277	Chr5	25712248
4278	Chr5	25712484
4279	Chr5	25819986
4280	Chr5	25868930
4281	Chr5	25868147
4282	Chr5	25980421
4283	Chr5	25980343
4284	Chr5	25989416
4285	Chr5	26025925
4286	Chr5	26025915
4287	Chr5	26025914
4288	Chr5	26025872
4289	Chr5	26042422
4290	Chr5	26042430
4291	Chr5	26042473
4292	Chr5	26042695
4293	Chr5	26042758
4294	Chr5	26043416
4295	Chr5	26043438
4296	Chr5	26043732
4297	Chr5	26043793
4298	Chr5	26048338
4299	Chr5	26048355
4300	Chr5	26048356
4301	Chr5	26048357
4302	Chr5	26048382
4303	Chr5	26048609
4304	Chr5	26048669
4305	Chr5	26048679
4306	Chr5	26050396
4307	Chr5	26050707
4308	Chr5	26050710
4309	Chr5	26096877
4310	Chr5	26110836
4311	Chr5	26110863
4312	Chr5	26111090
4313	Chr5	26110763
4314	Chr5	26110748
4315	Chr5	26110728
4316	Chr5	26110714
4317	Chr5	26178676
4318	Chr5	26179060
4319	Chr5	26209702
4320	Chr5	26209373
4321	Chr5	26319424

4322	Chr5	26327139
4323	Chr5	26386214
4324	Chr5	26386227
4325	Chr5	26386464
4326	Chr5	26413817
4327	Chr5	26413563
4328	Chr5	26413558
4329	Chr5	26459265
4330	Chr5	26459276
4331	Chr5	26472321
4332	Chr5	26472311
4333	Chr5	26476448
4334	Chr5	26476722
4335	Chr5	26505046
4336	Chr5	26524862
4337	Chr5	26524884
4338	Chr5	26524893
4339	Chr5	26524909
4340	Chr5	26526235
4341	Chr5	26525937
4342	Chr5	26546862
4343	Chr5	26546854
4344	Chr5	26553032
4345	Chr5	26575123
4346	Chr5	26579632
4347	Chr5	26580531
4348	Chr5	26611704
4349	Chr5	26628381
4350	Chr5	27042693
4351	Chr5	27335809
4352	Chr5	27335814
4353	Chr5	27354315
4354	Chr5	27392566
4355	Chr5	27392527
4356	Chr5	27393532
4357	Chr5	27393517
4358	Chr5	27429995
4359	Chr5	27429700
4360	Chr5	27558735
4361	Chr5	27569652
4362	Chr5	27639595
4363	Chr5	27639641
4364	Chr5	27639937
4365	Chr5	27645660
4366	Chr5	27645719
4367	Chr5	27651459
4368	Chr5	27696927

4369	Chr5	27696924
4370	Chr5	27752057
4371	Chr5	27833944
4372	Chr5	27836668
4373	Chr5	27836401
4374	Chr5	27836381
4375	Chr5	27864893
4376	Chr5	27864515
4377	Chr5	27864472
4378	Chr5	27874765
4379	Chr5	27874725
4380	Chr5	27874698
4381	Chr5	27919246
4382	Chr5	27919279
4383	Chr5	27937208
4384	Chr5	27959261
4385	Chr5	27959576
4386	Chr5	27974586
4387	Chr5	27974563
4388	Chr5	27974520
4389	Chr5	27974159
4390	Chr5	27974145
4391	Chr5	27978089
4392	Chr5	27978028
4393	Chr5	27977820
4394	Chr5	27977773
4395	Chr5	27994787
4396	Chr5	28103329
4397	Chr5	28103735
4398	Chr5	28238889
4399	Chr5	28238458
4400	Chr5	28322562
4401	Chr5	28330602
4402	Chr5	28332771
4403	Chr5	28333193
4404	Chr5	28333195
4405	Chr5	28333208
4406	Chr5	28332682
4407	Chr5	28332404
4408	Chr5	28352499
4409	Chr5	28352498
4410	Chr5	28384682
4411	Chr5	28384707
4412	Chr5	28384717
4413	Chr5	28384736
4414	Chr5	28384765
4415	Chr5	28385047

4416	Chr5	28431473
4417	Chr5	28476870
4418	Chr5	28476882
4419	Chr5	28476887
4420	Chr5	28477110
4421	Chr5	28509099
4422	Chr5	28509108
4423	Chr5	28509111
4424	Chr5	28533564
4425	Chr5	28533262
4426	Chr5	28533231
4427	Chr5	28533182
4428	Chr5	28576773
4429	Chr5	28611662
4430	Chr5	28611652
4431	Chr5	28611416
4432	Chr5	28611348
4433	Chr5	28611347
4434	Chr5	28701584
4435	Chr5	28701167
4436	Chr5	28985402
4437	Chr5	28985777
4438	Chr5	28985845
4439	Chr5	29146526
4440	Chr5	29179525
4441	Chr5	29179231
4442	Chr5	29184456
4443	Chr5	29252915
4444	Chr5	29299695
4445	Chr5	29317898
4446	Chr5	29335532
4447	Chr5	29423222
4448	Chr5	29423191
4449	Chr5	29432219
4450	Chr5	29442857
4451	Chr5	29442947
4452	Chr5	29471787
4453	Chr5	29471781
4454	Chr5	29504081
4455	Chr5	29503796
4456	Chr5	29510895
4457	Chr5	29591085
4458	Chr5	29591366
4459	Chr5	29590808
4460	Chr5	29590708
4461	Chr5	29594641
4462	Chr5	29594603

4463	Chr5	29616750
4464	Chr5	29616824
4465	Chr5	29617148
4466	Chr5	29617202
4467	Chr5	29653362
4468	Chr5	29653375
4469	Chr5	29653442
4470	Chr5	29672442
4471	Chr5	29672519
4472	Chr5	29672525
4473	Chr5	29679576
4474	Chr5	29679350
4475	Chr5	29679347
4476	Chr5	29709283
4477	Chr5	29708968
4478	Chr5	29708894
4479	Chr5	29718810
4480	Chr5	29748595
4481	Chr5	29748620
4482	Chr5	29748671
4483	Chr5	29748559
4484	Chr5	29748535
4485	Chr5	29748530
4486	Chr5	29815362
4487	Chr5	29867299
4488	Chr5	29867266
4489	Chr5	29867051
4490	Chr5	29866972
4491	Chr5	29899662
4492	Chr5	29907506
4493	Chr5	29907108
4494	Chr5	29907078
4495	Chr5	29907038
4496	Chr5	30013504
4497	Chr5	30013090
4498	Chr5	30013016
4499	Chr5	30031406
4500	Chr5	30031748
4501	Chr5	30038099
4502	Chr5	30100187
4503	Chr5	30100475
4504	Chr5	30100517
4505	Chr5	30162665
4506	Chr5	30162679
4507	Chr5	30162701
4508	Chr5	30162915
4509	Chr5	30162976

4510	Chr5	30163001
4511	Chr5	30166690
4512	Chr5	30166773
4513	Chr5	30255591
4514	Chr5	30259856
4515	Chr5	30277660
4516	Chr5	30277661
4517	Chr5	30277894
4518	Chr5	30278374
4519	Chr5	30278659
4520	Chr5	30350869
4521	Chr5	30351233
4522	Chr5	30350735
4523	Chr5	30350693
4524	Chr5	30370325
4525	Chr5	30370266
4526	Chr5	30370230
4527	Chr5	30370084
4528	Chr5	30370016
4529	Chr5	30425459
4530	Chr5	30425190
4531	Chr5	30493011
4532	Chr5	30492749
4533	Chr5	30526998
4534	Chr5	30526853
4535	Chr5	30526502
4536	Chr5	30615710
4537	Chr5	30642557
4538	Chr5	30775935
4539	Chr5	30776304
4540	Chr5	30776318
4541	Chr5	30776331
4542	Chr5	30776336
4543	Chr5	30800493
4544	Chr5	30800212
4545	Chr5	30814802
4546	Chr5	30815065
4547	Chr5	30815070
4548	Chr5	30953638
4549	Chr5	30953674
4550	Chr5	30985916
4551	Chr5	30986094
4552	Chr5	30986111
4553	Chr5	30986190
4554	Chr5	30993670
4555	Chr5	30993738
4556	Chr5	31033041

4557	Chr5	31034608
4558	Chr5	31037839
4559	Chr5	31046343
4560	Chr5	31049361
4561	Chr5	31049360
4562	Chr5	31199000
4563	Chr5	31198830
4564	Chr5	31198825
4565	Chr5	31255957
4566	Chr5	31255647
4567	Chr5	31255607
4568	Chr5	31255606
4569	Chr5	31255604
4570	Chr5	31255596
4571	Chr5	31255593
4572	Chr5	31301085
4573	Chr5	31301119
4574	Chr5	31301286
4575	Chr5	31346204
4576	Chr5	31345884
4577	Chr5	31389414
4578	Chr5	31389454
4579	Chr5	31389821
4580	Chr5	31401680
4581	Chr5	31420891
4582	Chr5	31420827
4583	Chr5	31691800
4584	Chr5	31691558
4585	Chr5	31698892
4586	Chr5	31699034
4587	Chr5	31699213
4588	Chr5	31699270
4589	Chr5	31746329
4590	Chr5	31807694
4591	Chr5	31843580
4592	Chr5	31879261
4593	Chr5	31879269
4594	Chr5	31879273
4595	Chr5	31879337
4596	Chr5	31879338
4597	Chr5	31879567
4598	Chr5	31880311
4599	Chr5	31880366
4600	Chr5	31880381
4601	Chr5	31922919
4602	Chr5	31922614
4603	Chr5	31925808

4604	Chr5	31925784
4605	Chr5	31935764
4606	Chr5	31935727
4607	Chr5	31935675
4608	Chr5	31982273
4609	Chr5	31982002
4610	Chr5	31991129
4611	Chr5	31991149
4612	Chr5	31991159
4613	Chr5	32036514
4614	Chr5	32036177
4615	Chr5	32036173
4616	Chr5	32105402
4617	Chr5	32105467
4618	Chr5	32109409
4619	Chr5	32109445
4620	Chr5	32109446
4621	Chr5	32109737
4622	Chr5	32182700
4623	Chr5	32213189
4624	Chr5	32213230
4625	Chr5	32213232
4626	Chr5	32213251
4627	Chr5	32213262
4628	Chr5	32213534
4629	Chr5	32213562
4630	Chr5	32213563
4631	Chr5	32213571
4632	Chr5	32213585
4633	Chr5	32213626
4634	Chr5	32254434
4635	Chr5	32254317
4636	Chr5	32254293
4637	Chr5	32254277
4638	Chr5	32254251
4639	Chr5	32254030
4640	Chr5	32277595
4641	Chr5	32277568
4642	Chr5	32277206
4643	Chr5	32277172
4644	Chr5	32278536
4645	Chr5	32278527
4646	Chr5	32278497
4647	Chr5	32278477
4648	Chr5	32285860
4649	Chr5	32285837
4650	Chr5	32285474

4651	Chr5	32312195
4652	Chr5	32311850
4653	Chr5	32311809
4654	Chr5	32311805
4655	Chr5	32321568
4656	Chr5	32321979
4657	Chr5	32321521
4658	Chr5	32321515
4659	Chr5	32321504
4660	Chr5	32321234
4661	Chr5	32336602
4662	Chr5	32336247
4663	Chr5	32336230
4664	Chr5	32384413
4665	Chr5	32384016
4666	Chr5	32383963
4667	Chr5	32385282
4668	Chr5	32385276
4669	Chr5	32385264
4670	Chr5	32432783
4671	Chr5	32432786
4672	Chr5	32432791
4673	Chr5	32433073
4674	Chr5	32463976
4675	Chr5	32463957
4676	Chr5	32463670
4677	Chr5	32463656
4678	Chr5	32463639
4679	Chr5	32463634
4680	Chr5	32463614
4681	Chr5	32511370
4682	Chr5	32546421
4683	Chr5	32546483
4684	Chr5	32550817
4685	Chr5	32550838
4686	Chr5	32550674
4687	Chr5	32550634
4688	Chr5	32553255
4689	Chr5	32553293
4690	Chr5	32553296
4691	Chr5	32553306
4692	Chr5	32553483
4693	Chr5	32553557
4694	Chr5	32608836
4695	Chr5	32677352
4696	Chr5	32677365
4697	Chr5	32677646

4698	Chr5	32677655
4699	Chr5	32798989
4700	Chr5	32798981
4701	Chr5	32851102
4702	Chr5	32851094
4703	Chr5	32851093
4704	Chr5	32851092
4705	Chr5	32851028
4706	Chr5	32851027
4707	Chr5	32895110
4708	Chr5	32895378
4709	Chr5	32895411
4710	Chr5	32898094
4711	Chr5	32898450
4712	Chr5	32958237
4713	Chr5	32994629
4714	Chr5	32994656
4715	Chr5	32994842
4716	Chr5	33036122
4717	Chr5	33036119
4718	Chr5	33036097
4719	Chr5	33036072
4720	Chr5	33035686
4721	Chr5	33035677
4722	Chr5	33047647
4723	Chr5	33061233
4724	Chr5	33061591
4725	Chr5	33061116
4726	Chr5	33060854
4727	Chr5	33060778
4728	Chr5	33073348
4729	Chr5	33073154
4730	Chr5	33073112
4731	Chr5	33076792
4732	Chr5	33076791
4733	Chr5	33078125
4734	Chr5	33078149
4735	Chr5	33141105
4736	Chr5	33141109
4737	Chr5	33141117
4738	Chr5	33141118
4739	Chr5	33141180
4740	Chr5	33141429
4741	Chr5	33148935
4742	Chr5	33148978
4743	Chr5	33148979
4744	Chr5	33149185

4745	Chr5	33154161
4746	Chr5	33154134
4747	Chr5	33154127
4748	Chr5	33153892
4749	Chr5	33159752
4750	Chr5	33159425
4751	Chr5	33159415
4752	Chr5	33159411
4753	Chr5	33159146
4754	Chr5	33160167
4755	Chr5	33160151
4756	Chr5	33159899
4757	Chr5	33159869
4758	Chr5	33159862
4759	Chr5	33159859
4760	Chr5	33162574
4761	Chr5	33162588
4762	Chr5	33162629
4763	Chr5	33162631
4764	Chr5	33162868
4765	Chr5	33182447
4766	Chr5	33182645
4767	Chr5	33182648
4768	Chr5	33221647
4769	Chr5	33221722
4770	Chr5	33221959
4771	Chr5	33221978
4772	Chr5	33268382
4773	Chr5	33268366
4774	Chr5	33268343
4775	Chr5	33268341
4776	Chr5	33268156
4777	Chr5	33268132
4778	Chr5	33309961
4779	Chr5	33310016
4780	Chr5	33310277
4781	Chr5	33310335
4782	Chr5	33344909
4783	Chr5	33344946
4784	Chr5	33344972
4785	Chr5	33345206
4786	Chr5	33345231
4787	Chr5	33345235
4788	Chr5	33345244
4789	Chr5	33345263
4790	Chr5	33345297
4791	Chr5	33345306

4792	Chr5	33345310
4793	Chr5	33383262
4794	Chr5	33383208
4795	Chr5	33382946
4796	Chr5	33418721
4797	Chr5	33418665
4798	Chr5	33418478
4799	Chr5	33418452
4800	Chr5	33418443
4801	Chr5	33418410
4802	Chr5	33445670
4803	Chr5	33445400
4804	Chr5	33445368
4805	Chr5	33445366
4806	Chr5	33445352
4807	Chr5	33446309
4808	Chr5	33504200
4809	Chr5	33504450
4810	Chr5	33504458
4811	Chr5	33504508
4812	Chr5	33527452
4813	Chr5	33527488
4814	Chr5	33527497
4815	Chr5	33527513
4816	Chr5	33527424
4817	Chr5	33527406
4818	Chr5	33527386
4819	Chr5	33527329
4820	Chr5	33527150
4821	Chr5	33532015
4822	Chr5	33531990
4823	Chr5	33546753
4824	Chr5	33546748
4825	Chr5	33546407
4826	Chr5	33546350
4827	Chr5	33546349
4828	Chr5	33548087
4829	Chr5	33548033
4830	Chr5	33547760
4831	Chr5	33547736
4832	Chr5	33550444
4833	Chr5	33550470
4834	Chr5	33550479
4835	Chr5	33550480
4836	Chr5	33550491
4837	Chr5	33550764
4838	Chr5	33632620

4839	Chr5	33632533
4840	Chr5	33632277
4841	Chr5	33632276
4842	Chr5	33632259
4843	Chr5	33632251
4844	Chr5	33642350
4845	Chr5	33642306
4846	Chr5	33641960
4847	Chr5	33641941
4848	Chr5	33664115
4849	Chr5	33664080
4850	Chr5	33664071
4851	Chr5	33663884
4852	Chr5	33663817
4853	Chr5	33663805
4854	Chr5	33663786
4855	Chr5	33674368
4856	Chr5	33674345
4857	Chr5	33674090
4858	Chr5	34098588
4859	Chr5	34098556
4860	Chr5	34098348
4861	Chr5	34098318
4862	Chr5	34098302
4863	Chr5	34285700
4864	Chr5	34286059
4865	Chr5	34286074
4866	Chr5	34286142
4867	Chr5	34303722
4868	Chr5	34303723
4869	Chr5	34303815
4870	Chr5	34330482
4871	Chr5	34330464
4872	Chr5	34330454
4873	Chr5	34424957
4874	Chr5	34425385
4875	Chr5	34459820
4876	Chr5	34459536
4877	Chr5	34459530
4878	Chr5	34552913
4879	Chr5	34552962
4880	Chr5	34552980
4881	Chr5	34553129
4882	Chr5	34553234
4883	Chr5	34562062
4884	Chr5	34562063
4885	Chr5	34562152

4886	Chr5	34641967
4887	Chr5	34641991
4888	Chr5	34823420
4889	Chr5	34823275
4890	Chr5	34902758
4891	Chr5	34928025
4892	Chr5	34927949
4893	Chr5	34927948
4894	Chr5	34927800
4895	Chr5	34927755
4896	Chr5	34927754
4897	Chr5	34927753
4898	Chr5	34927704
4899	Chr5	34927699
4900	Chr5	35093368
4901	Chr5	35093367
4902	Chr5	35093353
4903	Chr5	35093335
4904	Chr5	35093108
4905	Chr5	35101075
4906	Chr5	35126423
4907	Chr5	35126431
4908	Chr5	35205080
4909	Chr5	35205051
4910	Chr5	35205034
4911	Chr5	35205022
4912	Chr5	35335704
4913	Chr5	35335634
4914	Chr5	35335627
4915	Chr5	35335326
4916	Chr5	35387314
4917	Chr5	35410144
4918	Chr5	35410151
4919	Chr5	35605326
4920	Chr5	35605693
4921	Chr5	35605699
4922	Chr5	35718488
4923	Chr5	35718513
4924	Chr5	35718895
4925	Chr5	35785103
4926	Chr5	35785139
4927	Chr5	35785144
4928	Chr5	35802766
4929	Chr5	35886901
4930	Chr5	35887174
4931	Chr5	35887199
4932	Chr5	35887246

4933	Chr5	35900869
4934	Chr5	35900793
4935	Chr5	35900503
4936	Chr5	35900442
4937	Chr5	35901678
4938	Chr5	35907345
4939	Chr5	35907536
4940	Chr5	35907538
4941	Chr5	35907561
4942	Chr5	35907597
4943	Chr5	35934378
4944	Chr5	35957101
4945	Chr5	35957119
4946	Chr5	35957424
4947	Chr5	35957467
4948	Chr5	35957736
4949	Chr5	35957750
4950	Chr5	35957759
4951	Chr5	35957816
4952	Chr5	35957975
4953	Chr5	35957993
4954	Chr5	36007700
4955	Chr5	36008108
4956	Chr5	36008137
4957	Chr5	36032103
4958	Chr5	36032109
4959	Chr5	36032378
4960	Chr5	36048787
4961	Chr5	36053752
4962	Chr5	36148264
4963	Chr5	36148463
4964	Chr5	36149697
4965	Chr5	36199425
4966	Chr5	36199396
4967	Chr5	36254046
4968	Chr5	36254290
4969	Chr5	36283468
4970	Chr5	36283444
4971	Chr5	36322936
4972	Chr5	36322966
4973	Chr5	36323036
4974	Chr5	36323292
4975	Chr5	36325174
4976	Chr5	36375719
4977	Chr5	36375794
4978	Chr5	36375983
4979	Chr5	36376049

4980	Chr5	36394657
4981	Chr5	36429462
4982	Chr5	36453102
4983	Chr5	36453137
4984	Chr5	36453303
4985	Chr5	36453339
4986	Chr5	36453344
4987	Chr5	36472428
4988	Chr5	36557043
4989	Chr5	36557208
4990	Chr5	36557227
4991	Chr5	36557262
4992	Chr5	36558314
4993	Chr5	36659147
4994	Chr5	36659873
4995	Chr5	36659851
4996	Chr5	36659841
4997	Chr5	36659519
4998	Chr5	36719626
4999	Chr5	36729373
5000	Chr5	36817173
5001	Chr5	36821198
5002	Chr5	36875788
5003	Chr5	36909154
5004	Chr5	36909303
5005	Chr5	36908640
5006	Chr5	36908622
5007	Chr5	36908588
5008	Chr5	36931112
5009	Chr5	36931121
5010	Chr5	37005403
5011	Chr5	37047476
5012	Chr5	37047444
5013	Chr5	37047443
5014	Chr5	37047432
5015	Chr5	37047184
5016	Chr5	37047181
5017	Chr5	37047174
5018	Chr5	37047128
5019	Chr5	37047115
5020	Chr5	37047109
5021	Chr5	37056388
5022	Chr5	37077798
5023	Chr5	37078056
5024	Chr5	37078085
5025	Chr5	37078088
5026	Chr5	37078115

5027	Chr5	37078127
5028	Chr5	37109247
5029	Chr5	37109170
5030	Chr5	37151021
5031	Chr5	37256410
5032	Chr5	37256191
5033	Chr5	37387774
5034	Chr5	37387487
5035	Chr5	37422766
5036	Chr5	37467593
5037	Chr5	37467915
5038	Chr5	37482286
5039	Chr5	37482292
5040	Chr5	37482501
5041	Chr5	37482509
5042	Chr5	37482576
5043	Chr5	37482212
5044	Chr5	37482179
5045	Chr5	37482118
5046	Chr5	37481800
5047	Chr5	37508412
5048	Chr5	37508449
5049	Chr5	37508703
5050	Chr5	37509188
5051	Chr5	37508763
5052	Chr5	37508762
5053	Chr5	37508735
5054	Chr5	37670984
5055	Chr5	37670953
5056	Chr5	37670916
5057	Chr5	37670914
5058	Chr5	37670892
5059	Chr5	37670634
5060	Chr5	37670633
5061	Chr5	37670619
5062	Chr5	37673346
5063	Chr5	37673420
5064	Chr5	37673810
5065	Chr5	37674330
5066	Chr5	37674301
5067	Chr5	37685478
5068	Chr5	37685479
5069	Chr5	37685480
5070	Chr5	37685707
5071	Chr5	37685721
5072	Chr5	37685731
5073	Chr5	37711725

5074	Chr5	37712120
5075	Chr5	37712126
5076	Chr5	37712169
5077	Chr5	37712208
5078	Chr5	37711696
5079	Chr5	37711646
5080	Chr5	37711629
5081	Chr5	37728815
5082	Chr5	37728582
5083	Chr5	37728557
5084	Chr5	37745372
5085	Chr5	37745369
5086	Chr5	37745350
5087	Chr5	37745296
5088	Chr5	37745104
5089	Chr5	37755186
5090	Chr5	37755181
5091	Chr5	37755163
5092	Chr5	37754841
5093	Chr5	37754818
5094	Chr5	37754765
5095	Chr5	37754763
5096	Chr5	37760866
5097	Chr5	37779195
5098	Chr5	37779190
5099	Chr5	37779166
5100	Chr5	37778930
5101	Chr5	37778915
5102	Chr5	37778872
5103	Chr5	37793430
5104	Chr5	37793364
5105	Chr5	37793350
5106	Chr5	37793089
5107	Chr5	37806481
5108	Chr5	37806488
5109	Chr5	37806665
5110	Chr5	37806724
5111	Chr5	37841822
5112	Chr5	37841883
5113	Chr5	37842065
5114	Chr5	37842081
5115	Chr5	37841499
5116	Chr5	37906581
5117	Chr5	37922698
5118	Chr5	37922467
5119	Chr5	37922446
5120	Chr5	37922852

5121	Chr5	37922866
5122	Chr5	37963865
5123	Chr5	37963866
5124	Chr5	37963880
5125	Chr5	38052028
5126	Chr5	38052066
5127	Chr5	38052106
5128	Chr5	38063274
5129	Chr5	38063227
5130	Chr5	38063032
5131	Chr5	38128054
5132	Chr5	38196397
5133	Chr5	38226437
5134	Chr5	38226174
5135	Chr5	38226137
5136	Chr5	38476989
5137	Chr5	38477348
5138	Chr5	38490251
5139	Chr5	38553196
5140	Chr5	38553586
5141	Chr5	38553645
5142	Chr5	38553655
5143	Chr5	38614944
5144	Chr5	38615214
5145	Chr5	38615261
5146	Chr5	38615286
5147	Chr5	38615289
5148	Chr5	38614812
5149	Chr5	38614788
5150	Chr5	38614578
5151	Chr5	38614548
5152	Chr5	38619771
5153	Chr5	38619765
5154	Chr5	38619740
5155	Chr5	38619723
5156	Chr5	38619722
5157	Chr5	38626127
5158	Chr5	38626148
5159	Chr5	38772140
5160	Chr5	38771828
5161	Chr5	38817571
5162	Chr5	38817329
5163	Chr5	38817251
5164	Chr5	38848907
5165	Chr5	38849213
5166	Chr5	38849235
5167	Chr5	38848756

5168	Chr5	38851020
5169	Chr5	38862486
5170	Chr5	38862460
5171	Chr5	38870917
5172	Chr5	38870857
5173	Chr5	38870495
5174	Chr5	38870488
5175	Chr5	38870473
5176	Chr5	38874701
5177	Chr5	38874700
5178	Chr5	38874353
5179	Chr5	38913839
5180	Chr5	38950303
5181	Chr5	38968585
5182	Chr5	38968639
5183	Chr5	38975846
5184	Chr5	38975920
5185	Chr5	38978627
5186	Chr5	38978417
5187	Chr5	38978406
5188	Chr5	38978388
5189	Chr5	38978383
5190	Chr5	38996587
5191	Chr5	38996630
5192	Chr5	38996641
5193	Chr5	38996665
5194	Chr5	39003763
5195	Chr5	39003822
5196	Chr5	39026422
5197	Chr5	39026477
5198	Chr5	39043181
5199	Chr5	39043355
5200	Chr5	39043396
5201	Chr5	39057407
5202	Chr5	39057368
5203	Chr5	39059312
5204	Chr5	39059324
5205	Chr5	39059371
5206	Chr5	39152018
5207	Chr5	39151752
5208	Chr5	39151738
5209	Chr5	39225644
5210	Chr5	39225714
5211	Chr5	39226880
5212	Chr5	39226867
5213	Chr5	39290652
5214	Chr5	39290585

5215	Chr5	39290568
5216	Chr5	39294692
5217	Chr5	39294725
5218	Chr5	39310757
5219	Chr5	39324854
5220	Chr5	39324864
5221	Chr5	39324868
5222	Chr5	39324885
5223	Chr5	39324946
5224	Chr5	39325115
5225	Chr5	39325187
5226	Chr5	39335797
5227	Chr5	39353400
5228	Chr5	39353398
5229	Chr5	39358029
5230	Chr5	39357883
5231	Chr5	39445288
5232	Chr5	39445257
5233	Chr5	39445228
5234	Chr5	39462747
5235	Chr5	39465197
5236	Chr5	39465226
5237	Chr5	39465278
5238	Chr5	39467530
5239	Chr5	39467300
5240	Chr5	39467250
5241	Chr5	39467245
5242	Chr5	39557147
5243	Chr5	39556737
5244	Chr5	39556733
5245	Chr5	39556717
5246	Chr5	39557792
5247	Chr5	39557426
5248	Chr5	39557406
5249	Chr5	39557390
5250	Chr5	39557389
5251	Chr5	39559954
5252	Chr5	39559949
5253	Chr5	39559942
5254	Chr5	39651682
5255	Chr5	39651695
5256	Chr5	39680581
5257	Chr5	39681828
5258	Chr5	39681894
5259	Chr5	39681897
5260	Chr5	39681905
5261	Chr5	39681792

5262	Chr5	39681712
5263	Chr5	39790841
5264	Chr5	39872330
5265	Chr5	39872386
5266	Chr5	39887301
5267	Chr5	39887225
5268	Chr5	39895884
5269	Chr5	39927060
5270	Chr5	39927230
5271	Chr5	39930112
5272	Chr5	39929834
5273	Chr5	39929805
5274	Chr5	40025071
5275	Chr5	40241410
5276	Chr5	40265587
5277	Chr5	40265442
5278	Chr5	40284079
5279	Chr5	40284137
5280	Chr5	40284145
5281	Chr5	40299800
5282	Chr5	40299777
5283	Chr5	40299773
5284	Chr5	40299765
5285	Chr5	40299722
5286	Chr5	40304638
5287	Chr5	40304925
5288	Chr5	40304969
5289	Chr5	40363902
5290	Chr5	40363594
5291	Chr5	40363586
5292	Chr5	40363565
5293	Chr5	40417915
5294	Chr5	40417543
5295	Chr5	40433705
5296	Chr5	40447218
5297	Chr5	40446952
5298	Chr5	40476731
5299	Chr5	40476395
5300	Chr5	40477926
5301	Chr5	40539935
5302	Chr5	40540054
5303	Chr5	40584438
5304	Chr5	40584425
5305	Chr5	40584422
5306	Chr5	40584401
5307	Chr5	40584359
5308	Chr5	40584045

5309	Chr5	40613317
5310	Chr5	40613372
5311	Chr5	40613736
5312	Chr5	40643280
5313	Chr5	40643261
5314	Chr5	40643250
5315	Chr5	40642969
5316	Chr5	40642947
5317	Chr5	40684759
5318	Chr5	40684736
5319	Chr5	40684439
5320	Chr5	40684383
5321	Chr5	40732664
5322	Chr5	40732656
5323	Chr5	40759743
5324	Chr5	40759772
5325	Chr5	40759821
5326	Chr5	40759826
5327	Chr5	40759988
5328	Chr5	40760690
5329	Chr5	40760718
5330	Chr5	40760721
5331	Chr5	40761007
5332	Chr5	40768857
5333	Chr5	40813830
5334	Chr5	40814131
5335	Chr5	40854214
5336	Chr5	40889213
5337	Chr5	40920987
5338	Chr5	40920772
5339	Chr5	40920720
5340	Chr5	40974336
5341	Chr5	40974345
5342	Chr5	41040012
5343	Chr5	41045392
5344	Chr5	41045437
5345	Chr5	41045287
5346	Chr5	41098779
5347	Chr5	41111041
5348	Chr5	41220532
5349	Chr5	41220281
5350	Chr5	41227636
5351	Chr5	41230551
5352	Chr5	41230811
5353	Chr5	41235202
5354	Chr5	41237884
5355	Chr5	41238146

5356	Chr5	41272045
5357	Chr5	41279732
5358	Chr5	41279671
5359	Chr5	41279417
5360	Chr5	41302298
5361	Chr5	41302265
5362	Chr5	41301855
5363	Chr5	41336358
5364	Chr5	41339741
5365	Chr5	41339699
5366	Chr5	41339519
5367	Chr5	41339517
5368	Chr5	41339443
5369	Chr5	41371678
5370	Chr5	41451388
5371	Chr5	41451424
5372	Chr5	41451793
5373	Chr5	41568387
5374	Chr5	41568721
5375	Chr5	41568752
5376	Chr5	41568761
5377	Chr5	41568299
5378	Chr5	41639846
5379	Chr5	41639979
5380	Chr5	41666607
5381	Chr5	41666659
5382	Chr5	41697095
5383	Chr5	41742824
5384	Chr5	41849542
5385	Chr5	41886753
5386	Chr5	41891482
5387	Chr5	41891712
5388	Chr5	41916407
5389	Chr5	41916376
5390	Chr5	41916340
5391	Chr5	41916135
5392	Chr5	41916123
5393	Chr5	41937184
5394	Chr5	43109748
5395	Chr5	43115358
5396	Chr5	43117735
5397	Chr5	43117706
5398	Chr5	43189954
5399	Chr5	43190955
5400	Chr5	43190970
5401	Chr5	43238623
5402	Chr5	43238631

5403	Chr5	43238203
5404	Chr5	43237922
5405	Chr5	43249353
5406	Chr5	43294040
5407	Chr5	43319532
5408	Chr5	43319529
5409	Chr5	43319489
5410	Chr5	43319482
5411	Chr5	43319477
5412	Chr5	43325491
5413	Chr5	43456445
5414	Chr5	43456485
5415	Chr5	43456793
5416	Chr5	43456835
5417	Chr5	43456851
5418	Chr5	43503766
5419	Chr5	43503723
5420	Chr5	43503452
5421	Chr5	43539483
5422	Chr5	43539870
5423	Chr5	43539440
5424	Chr5	43579042
5425	Chr5	43636538
5426	Chr5	43636522
5427	Chr5	43636514
5428	Chr5	43636509
5429	Chr5	43636476
5430	Chr5	43645440
5431	Chr5	43645067
5432	Chr5	43654177
5433	Chr5	43654572
5434	Chr5	43687869
5435	Chr5	43687622
5436	Chr5	43687578
5437	Chr5	43687567
5438	Chr5	43694607
5439	Chr5	43694620
5440	Chr5	43694833
5441	Chr5	43790065
5442	Chr5	43789711
5443	Chr5	43798867
5444	Chr5	43798924
5445	Chr5	43799147
5446	Chr5	43922424
5447	Chr5	43922432
5448	Chr5	43935148
5449	Chr5	43935219

5450	Chr5	43946165
5451	Chr5	43946180
5452	Chr5	43946217
5453	Chr5	43946233
5454	Chr5	43946833
5455	Chr5	43947016
5456	Chr5	44022088
5457	Chr5	44121983
5458	Chr5	44131079
5459	Chr5	44131093
5460	Chr5	44193861
5461	Chr5	44193817
5462	Chr5	44193507
5463	Chr5	44193474
5464	Chr5	44229770
5465	Chr5	44229767
5466	Chr5	44229766
5467	Chr5	44229736
5468	Chr5	44229708
5469	Chr5	44236381
5470	Chr5	44235997
5471	Chr5	44235983
5472	Chr5	44235979
5473	Chr5	44235917
5474	Chr5	44259074
5475	Chr5	44268784
5476	Chr5	44268797
5477	Chr5	44268842
5478	Chr5	44268843
5479	Chr5	44324706
5480	Chr5	44324779
5481	Chr5	44325018
5482	Chr5	44325038
5483	Chr5	44325056
5484	Chr5	44392979
5485	Chr5	44392992
5486	Chr5	44393010
5487	Chr5	44393032
5488	Chr5	44393283
5489	Chr5	44393305
5490	Chr5	44393354
5491	Chr5	44429488
5492	Chr5	44429191
5493	Chr5	44429126
5494	Chr5	44429121
5495	Chr5	44457382
5496	Chr5	44457361

5497	Chr5	44508619
5498	Chr5	44508371
5499	Chr5	44519741
5500	Chr5	44519774
5501	Chr5	44520164
5502	Chr5	44539437
5503	Chr5	44539460
5504	Chr5	44539509
5505	Chr5	44539690
5506	Chr5	44539704
5507	Chr5	44539707
5508	Chr5	44539717
5509	Chr5	44539770
5510	Chr5	44539372
5511	Chr5	44539349
5512	Chr5	44539314
5513	Chr5	44539136
5514	Chr5	44539111
5515	Chr5	44539084
5516	Chr5	44549049
5517	Chr5	44549052
5518	Chr5	44549354
5519	Chr5	44577979
5520	Chr5	44577956
5521	Chr5	44618130
5522	Chr5	44775381
5523	Chr5	44775365
5524	Chr5	44775042
5525	Chr5	44801564
5526	Chr5	44801795
5527	Chr5	44870774
5528	Chr5	44870769
5529	Chr5	44870761
5530	Chr5	45236797
5531	Chr5	45236895
5532	Chr5	45237131
5533	Chr5	45237160
5534	Chr5	45348721
5535	Chr5	45348438
5536	Chr5	45348419
5537	Chr5	45348392
5538	Chr5	45348372
5539	Chr5	45375976
5540	Chr5	45421189
5541	Chr5	45421214
5542	Chr5	45421254
5543	Chr5	45478309

5544	Chr5	45478300
5545	Chr5	45478285
5546	Chr5	45496341
5547	Chr5	45496388
5548	Chr5	45679375
5549	Chr5	45705977
5550	Chr5	45705979
5551	Chr5	45706011
5552	Chr5	45706173
5553	Chr5	45706202
5554	Chr5	45715594
5555	Chr5	45715804
5556	Chr5	45715806
5557	Chr5	45715818
5558	Chr5	45715845
5559	Chr5	45719816
5560	Chr5	45719829
5561	Chr5	45719843
5562	Chr5	45727484
5563	Chr5	45727438
5564	Chr5	45768821
5565	Chr5	45775955
5566	Chr5	45776382
5567	Chr5	45776416
5568	Chr5	45776440
5569	Chr5	45792986
5570	Chr5	45821718
5571	Chr5	45821778
5572	Chr5	45821796
5573	Chr5	45877841
5574	Chr5	45877782
5575	Chr5	45877779
5576	Chr5	45877540
5577	Chr5	45883163
5578	Chr5	45885593
5579	Chr5	45885779
5580	Chr5	45885793
5581	Chr5	45933725
5582	Chr5	45934106
5583	Chr5	45988387
5584	Chr5	45996362
5585	Chr5	46040658
5586	Chr5	46041014
5587	Chr5	46041053
5588	Chr5	46041066
5589	Chr5	46041092
5590	Chr5	46046394

5591	Chr5	46046405
5592	Chr5	46046831
5593	Chr5	46048867
5594	Chr5	46091427
5595	Chr5	46091398
5596	Chr5	46091047
5597	Chr5	46091035
5598	Chr5	46091900
5599	Chr5	46091906
5600	Chr5	46091950
5601	Chr5	46092129
5602	Chr5	46156937
5603	Chr5	46156936
5604	Chr5	46156905
5605	Chr5	46156588
5606	Chr5	46156586
5607	Chr5	46156573
5608	Chr5	46216767
5609	Chr5	46220489
5610	Chr5	46236408
5611	Chr5	46236663
5612	Chr5	46237384
5613	Chr5	46237244
5614	Chr5	46237195
5615	Chr5	46288546
5616	Chr5	46311528
5617	Chr5	46311549
5618	Chr5	46351467
5619	Chr5	46388870
5620	Chr5	46389269
5621	Chr5	46405136
5622	Chr5	46405154
5623	Chr5	46405511
5624	Chr5	46405055
5625	Chr5	46536916
5626	Chr5	46536513
5627	Chr5	46536508
5628	Chr5	46536480
5629	Chr5	46540429
5630	Chr5	46540329
5631	Chr5	46543187
5632	Chr5	46566882
5633	Chr5	46571401
5634	Chr5	46571731
5635	Chr5	46571801
5636	Chr5	46629933
5637	Chr5	46629897

5638	Chr5	46629647
5639	Chr5	46629633
5640	Chr5	46662500
5641	Chr5	46662461
5642	Chr5	46662452
5643	Chr5	46671545
5644	Chr5	46671530
5645	Chr5	46671510
5646	Chr5	46762338
5647	Chr5	46838366
5648	Chr5	46869657
5649	Chr5	46869284
5650	Chr5	46969366
5651	Chr5	46969011
5652	Chr5	46968958
5653	Chr5	47070675
5654	Chr5	47085059
5655	Chr5	47089299
5656	Chr5	47089034
5657	Chr5	47088952
5658	Chr5	47108139
5659	Chr5	47108102
5660	Chr5	47183669
5661	Chr5	47183673
5662	Chr5	47193219
5663	Chr5	47197749
5664	Chr5	47197774
5665	Chr5	47197796
5666	Chr5	47197938
5667	Chr5	47197949
5668	Chr5	47198038
5669	Chr5	47283280
5670	Chr5	47282915
5671	Chr5	48181824
5672	Chr5	48181797
5673	Chr5	48217316
5674	Chr5	48217312
5675	Chr5	48217294
5676	Chr5	48217090
5677	Chr5	48217057
5678	Chr5	48219000
5679	Chr5	48242362
5680	Chr5	48241968
5681	Chr5	48342970
5682	Chr5	48342955
5683	Chr5	48342930
5684	Chr5	48342911

5685	Chr5	48362309
5686	Chr5	48362325
5687	Chr5	48401525
5688	Chr5	48481680
5689	Chr5	48481678
5690	Chr5	48481673
5691	Chr5	48481660
5692	Chr5	48481263
5693	Chr5	48575112
5694	Chr5	48575353
5695	Chr5	48575381
5696	Chr5	48575382
5697	Chr5	48575018
5698	Chr5	48575007
5699	Chr5	48574645
5700	Chr5	48598343
5701	Chr5	48598338
5702	Chr5	48614317
5703	Chr5	48665131
5704	Chr5	48665158
5705	Chr5	48665182
5706	Chr5	48701242
5707	Chr5	48701289
5708	Chr5	48701627
5709	Chr5	48701655
5710	Chr5	48898425
5711	Chr5	48988722
5712	Chr5	48988725
5713	Chr5	48988633
5714	Chr5	48988546
5715	Chr5	48988413
5716	Chr5	49077088
5717	Chr5	49077093
5718	Chr5	49077095
5719	Chr5	49077114
5720	Chr5	49077416
5721	Chr5	49103619
5722	Chr5	49133033
5723	Chr5	49133218
5724	Chr5	49133235
5725	Chr5	49133244
5726	Chr5	49132723
5727	Chr5	49154851
5728	Chr5	49160893
5729	Chr5	49160841
5730	Chr5	49165475
5731	Chr5	49165130

5732	Chr5	49251963
5733	Chr5	49251965
5734	Chr5	49298833
5735	Chr5	49298834
5736	Chr5	49298905
5737	Chr5	49299066
5738	Chr5	49299090
5739	Chr5	49299130
5740	Chr5	49299138
5741	Chr5	49299143
5742	Chr5	49299145
5743	Chr5	49299149
5744	Chr5	49307174
5745	Chr5	49307220
5746	Chr5	49307621
5747	Chr5	49307634
5748	Chr5	49307053
5749	Chr5	49306678
5750	Chr5	49309164
5751	Chr5	49346680
5752	Chr5	49346696
5753	Chr5	49346741
5754	Chr5	49346767
5755	Chr5	49346769
5756	Chr5	49378107
5757	Chr5	49378154
5758	Chr5	49378325
5759	Chr5	49378038
5760	Chr5	49377995
5761	Chr5	49377691
5762	Chr5	49377683
5763	Chr5	49471443
5764	Chr5	49489847
5765	Chr5	49489857
5766	Chr5	49489858
5767	Chr5	49489873
5768	Chr5	49489904
5769	Chr5	49490041
5770	Chr5	49506269
5771	Chr5	49506334
5772	Chr5	49527425
5773	Chr5	49527263
5774	Chr5	49527253
5775	Chr5	49526976
5776	Chr5	49526953
5777	Chr5	49526948
5778	Chr5	49526946

5779	Chr5	49586056
5780	Chr5	49586007
5781	Chr5	49585809
5782	Chr5	49627122
5783	Chr5	49627365
5784	Chr5	49627449
5785	Chr5	49755250
5786	Chr5	49780507
5787	Chr5	49780700
5788	Chr5	49798319
5789	Chr5	49798519
5790	Chr5	49798544
5791	Chr5	49825828
5792	Chr5	49859619
5793	Chr5	49872413
5794	Chr5	49958068
5795	Chr5	49957990
5796	Chr5	49957626
5797	Chr5	50070108
5798	Chr5	50069898
5799	Chr5	50069879
5800	Chr5	50083585
5801	Chr5	50084033
5802	Chr5	50267753
5803	Chr5	50313636
5804	Chr5	50313658
5805	Chr5	50313905
5806	Chr5	50313927
5807	Chr5	50313491
5808	Chr5	50313288
5809	Chr5	50313270
5810	Chr5	50313269
5811	Chr5	50313254
5812	Chr5	50504142
5813	Chr5	50505194
5814	Chr5	50505003
5815	Chr5	50591720
5816	Chr5	50591711
5817	Chr5	50591630
5818	Chr5	50627037
5819	Chr5	50636978
5820	Chr5	50636795
5821	Chr5	50636749
5822	Chr5	50636746
5823	Chr5	50732292
5824	Chr5	50732678
5825	Chr5	50733931

5826	Chr5	50758177
5827	Chr5	50758149
5828	Chr5	50757900
5829	Chr5	50757891
5830	Chr5	50774781
5831	Chr5	50774782
5832	Chr5	50774795
5833	Chr5	50774796
5834	Chr5	50775018
5835	Chr5	50775044
5836	Chr5	50775051
5837	Chr5	50775072
5838	Chr5	50813789
5839	Chr5	50814028
5840	Chr5	50813342
5841	Chr5	50819192
5842	Chr5	50819215
5843	Chr5	50819260
5844	Chr5	50819583
5845	Chr5	50890770
5846	Chr5	50922273
5847	Chr5	50922265
5848	Chr5	50922251
5849	Chr5	50922012
5850	Chr5	50921988
5851	Chr5	50921986
5852	Chr5	50973153
5853	Chr5	50973210
5854	Chr5	50973488
5855	Chr5	50974073
5856	Chr5	50980870
5857	Chr5	50994666
5858	Chr5	51025660
5859	Chr5	51025679
5860	Chr5	51025680
5861	Chr5	51025969
5862	Chr5	51026023
5863	Chr5	51027041
5864	Chr5	51027046
5865	Chr5	51027317
5866	Chr5	51038096
5867	Chr5	51038162
5868	Chr5	51038405
5869	Chr5	51128983
5870	Chr5	51151102
5871	Chr5	51151082
5872	Chr5	51200069

5873	Chr5	51200087
5874	Chr5	51200145
5875	Chr5	51240327
5876	Chr5	51240305
5877	Chr5	51240271
5878	Chr5	51240033
5879	Chr5	51244169
5880	Chr5	51323876
5881	Chr5	51334861
5882	Chr5	51334724
5883	Chr5	51334473
5884	Chr5	51355958
5885	Chr5	51356171
5886	Chr5	51461910
5887	Chr5	51461869
5888	Chr5	51461565
5889	Chr5	51461528
5890	Chr5	51461517
5891	Chr5	51544612
5892	Chr5	51544352
5893	Chr5	51544268
5894	Chr5	51544265
5895	Chr5	51546071
5896	Chr5	51545764
5897	Chr5	51591317
5898	Chr5	51591351
5899	Chr5	51615872
5900	Chr5	51615897
5901	Chr5	51662443
5902	Chr5	51684546
5903	Chr5	51684736
5904	Chr5	51684789
5905	Chr5	51703635
5906	Chr5	51703609
5907	Chr5	51703585
5908	Chr5	51748283
5909	Chr5	51748270
5910	Chr5	51748225
5911	Chr5	51752298
5912	Chr5	51752295
5913	Chr5	51752015
5914	Chr5	51751990
5915	Chr5	51770752
5916	Chr5	51770773
5917	Chr5	51770443
5918	Chr5	51770411
5919	Chr5	51770233

5920	Chr5	51770200
5921	Chr5	51782127
5922	Chr5	51809509
5923	Chr5	51809512
5924	Chr5	51836708
5925	Chr5	51837103
5926	Chr5	51836806
5927	Chr5	51843114
5928	Chr5	51843091
5929	Chr5	51843068
5930	Chr5	51843029
5931	Chr5	51842700
5932	Chr5	51842663
5933	Chr5	51842661
5934	Chr5	51876037
5935	Chr5	51876121
5936	Chr5	51876329
5937	Chr5	51881790
5938	Chr5	51882012
5939	Chr5	51914504
5940	Chr5	51914451
5941	Chr5	51914266
5942	Chr5	51976307
5943	Chr5	51976369
5944	Chr5	51976560
5945	Chr5	51976597
5946	Chr5	52075168
5947	Chr5	52075469
5948	Chr5	52075478
5949	Chr5	52075493
5950	Chr5	52075069
5951	Chr5	52115512
5952	Chr5	52130953
5953	Chr5	52130917
5954	Chr5	52130903
5955	Chr5	52150874
5956	Chr5	52177904
5957	Chr5	52177877
5958	Chr5	52191225
5959	Chr5	52259437
5960	Chr5	52264533
5961	Chr5	52282280
5962	Chr5	52282277
5963	Chr5	52282212
5964	Chr5	52306299
5965	Chr5	52357573
5966	Chr5	52381298

5967	Chr5	52518020
5968	Chr5	52590169
5969	Chr5	52590204
5970	Chr5	52591766
5971	Chr5	52628034
5972	Chr5	52627620
5973	Chr5	52685526
5974	Chr5	52688678
5975	Chr5	52688330
5976	Chr5	52688283
5977	Chr5	52717088
5978	Chr5	52806902
5979	Chr5	52838460
5980	Chr5	52843057
5981	Chr5	52843021
5982	Chr5	52843006
5983	Chr5	52842803
5984	Chr5	52842719
5985	Chr5	52898953
5986	Chr5	52898930
5987	Chr5	52898888
5988	Chr5	52898637
5989	Chr5	52898625
5990	Chr5	52923380
5991	Chr5	52923451
5992	Chr5	52923690
5993	Chr5	52924090
5994	Chr5	52931442
5995	Chr5	52931472
5996	Chr5	53003636
5997	Chr5	53008908
5998	Chr5	53025965
5999	Chr5	53026007
6000	Chr5	53026017
6001	Chr5	53045386
6002	Chr5	53119049
6003	Chr5	53129990
6004	Chr5	53129946
6005	Chr5	53135121
6006	Chr5	53155150
6007	Chr5	53179593
6008	Chr5	53179642
6009	Chr5	53179151
6010	Chr5	53179145
6011	Chr5	53252829
6012	Chr5	53253036
6013	Chr5	53253082

6014	Chr5	53261757
6015	Chr5	53261749
6016	Chr5	53261545
6017	Chr5	53261534
6018	Chr5	53261522
6019	Chr5	53261494
6020	Chr5	53261453
6021	Chr5	53264294
6022	Chr5	53264180
6023	Chr5	53264128
6024	Chr5	53263805
6025	Chr5	53283231
6026	Chr5	53283261
6027	Chr5	53283298
6028	Chr5	53283550
6029	Chr5	53283609
6030	Chr5	53283096
6031	Chr5	53282860
6032	Chr5	53407594
6033	Chr5	53446038
6034	Chr5	53446385
6035	Chr5	53446399
6036	Chr5	53451592
6037	Chr5	53451611
6038	Chr5	53451662
6039	Chr5	53452016
6040	Chr5	53502483
6041	Chr5	53572329
6042	Chr5	53572301
6043	Chr5	53611229
6044	Chr5	53611598
6045	Chr5	53616702
6046	Chr5	53616704
6047	Chr5	53616712
6048	Chr5	53616972
6049	Chr5	53616995
6050	Chr5	53616622
6051	Chr5	53616619
6052	Chr5	53616284
6053	Chr5	53627279
6054	Chr5	53633065
6055	Chr5	53633073
6056	Chr5	53633076
6057	Chr5	53633450
6058	Chr5	53704065
6059	Chr5	53704449
6060	Chr5	53831119

6061	Chr5	53870700
6062	Chr5	53870706
6063	Chr5	53870718
6064	Chr5	53870982
6065	Chr5	53895814
6066	Chr5	53895866
6067	Chr5	53895573
6068	Chr5	53901342
6069	Chr5	53901362
6070	Chr5	53901672
6071	Chr5	53940152
6072	Chr5	53984112
6073	Chr5	53984191
6074	Chr5	53996662
6075	Chr5	53996648
6076	Chr5	53996259
6077	Chr5	53996249
6078	Chr5	53996237
6079	Chr5	53996214
6080	Chr5	53998598
6081	Chr5	53998551
6082	Chr5	53998511
6083	Chr5	53998505
6084	Chr5	53998265
6085	Chr5	53998172
6086	Chr5	54003389
6087	Chr5	54003357
6088	Chr5	54003082
6089	Chr5	54003012
6090	Chr5	54003003
6091	Chr5	54050975
6092	Chr5	54050764
6093	Chr5	54063245
6094	Chr5	54063459
6095	Chr5	54090041
6096	Chr5	54152078
6097	Chr5	54152246
6098	Chr5	54152331
6099	Chr5	54152340
6100	Chr5	54217541
6101	Chr5	54217540
6102	Chr5	54217134
6103	Chr5	54250718
6104	Chr5	54250703
6105	Chr5	54320887
6106	Chr5	54326208
6107	Chr5	54326601

6108	Chr5	54326603
6109	Chr5	54326104
6110	Chr5	54325860
6111	Chr5	54385229
6112	Chr5	54398854
6113	Chr5	54399064
6114	Chr5	54455889
6115	Chr5	54455874
6116	Chr5	54455633
6117	Chr5	54455567
6118	Chr5	54541470
6119	Chr5	54541526
6120	Chr5	54541560
6121	Chr5	54584366
6122	Chr5	54584351
6123	Chr5	54584338
6124	Chr5	54584290
6125	Chr5	54585554
6126	Chr5	54585582
6127	Chr5	54684375
6128	Chr5	54684383
6129	Chr5	54684674
6130	Chr5	54755898
6131	Chr5	54755914
6132	Chr5	54756157
6133	Chr5	54759727
6134	Chr5	54760006
6135	Chr5	54760045
6136	Chr5	54811184
6137	Chr5	54810900
6138	Chr5	54849103
6139	Chr5	54849452
6140	Chr5	54849479
6141	Chr5	54849520
6142	Chr5	54862378
6143	Chr5	54862343
6144	Chr5	54861975
6145	Chr5	54976133
6146	Chr5	54976106
6147	Chr5	54977275
6148	Chr5	54977230
6149	Chr5	54991148
6150	Chr5	54991103
6151	Chr5	54990895
6152	Chr5	55113141
6153	Chr5	55112922
6154	Chr5	55112880

6155	Chr5	55133015
6156	Chr5	55184211
6157	Chr5	55191118
6158	Chr5	55236661
6159	Chr5	55241717
6160	Chr5	55253465
6161	Chr5	55253764
6162	Chr5	55361355
6163	Chr5	55401233
6164	Chr5	55432386
6165	Chr5	55549342
6166	Chr5	55559568
6167	Chr5	55559415
6168	Chr5	55559327
6169	Chr5	55589541
6170	Chr5	55589291
6171	Chr5	55589262
6172	Chr5	55610183
6173	Chr5	55610005
6174	Chr5	55624274
6175	Chr5	55670737
6176	Chr5	55675897
6177	Chr5	55780234
6178	Chr5	55798488
6179	Chr5	55798531
6180	Chr5	55800770
6181	Chr5	55852357
6182	Chr5	55875352
6183	Chr5	55886601
6184	Chr5	55886653
6185	Chr5	55903629
6186	Chr5	55931475
6187	Chr5	55931490
6188	Chr5	55958772
6189	Chr5	55958987
6190	Chr5	55959040
6191	Chr5	56099808
6192	Chr5	56099804
6193	Chr5	56099755
6194	Chr5	56099720
6195	Chr5	56099521
6196	Chr5	56099452
6197	Chr5	56103787
6198	Chr5	56104793
6199	Chr5	56106544
6200	Chr5	56106512
6201	Chr5	56106242

6202	Chr5	56180580
6203	Chr5	56216108
6204	Chr5	56358167
6205	Chr5	56358119
6206	Chr5	56390144
6207	Chr5	56390458
6208	Chr5	56390477
6209	Chr5	56397620
6210	Chr5	56400762
6211	Chr5	56455217
6212	Chr5	56455225
6213	Chr5	56455259
6214	Chr5	56455527
6215	Chr5	56470468
6216	Chr5	56470781
6217	Chr5	56472636
6218	Chr5	56472660
6219	Chr5	56472668
6220	Chr5	56472691
6221	Chr5	56472862
6222	Chr5	56472867
6223	Chr5	56472537
6224	Chr5	56472507
6225	Chr5	56576783
6226	Chr5	56576802
6227	Chr5	56577028
6228	Chr5	56577095
6229	Chr5	56577096
6230	Chr5	56576290
6231	Chr5	56634796
6232	Chr5	56634819
6233	Chr5	56635217
6234	Chr5	56635229
6235	Chr5	56635235
6236	Chr5	56649015
6237	Chr5	56649269
6238	Chr5	56648951
6239	Chr5	56648638
6240	Chr5	56932702
6241	Chr5	56932367
6242	Chr5	56936563
6243	Chr5	56964263
6244	Chr5	56963828
6245	Chr5	56970651
6246	Chr5	56970674
6247	Chr5	56970686
6248	Chr5	56970852

6249	Chr5	56970883
6250	Chr5	57014828
6251	Chr5	57063919
6252	Chr5	57064095
6253	Chr5	57063510
6254	Chr5	57069824
6255	Chr5	57069801
6256	Chr5	57069612
6257	Chr5	57069592
6258	Chr5	57105814
6259	Chr5	57105810
6260	Chr5	57127377
6261	Chr5	57127434
6262	Chr5	57247458
6263	Chr5	57250120
6264	Chr5	57261126
6265	Chr5	57260835
6266	Chr5	57263830
6267	Chr5	57278560
6268	Chr5	57278486
6269	Chr5	57280467
6270	Chr5	57280766
6271	Chr5	57280784
6272	Chr5	57327198
6273	Chr5	57326934
6274	Chr5	57326879
6275	Chr5	57380229
6276	Chr5	57379870
6277	Chr5	57444074
6278	Chr5	57577369
6279	Chr5	57587042
6280	Chr5	57586698
6281	Chr5	57586681
6282	Chr5	57617809
6283	Chr5	57644120
6284	Chr5	57724338
6285	Chr5	57727444
6286	Chr5	57727136
6287	Chr5	57727134
6288	Chr5	57729886
6289	Chr5	57729942
6290	Chr5	57748297
6291	Chr5	57748254
6292	Chr5	57747994
6293	Chr5	57749112
6294	Chr5	57770818
6295	Chr5	57770465

6296	Chr5	57770421
6297	Chr5	57770420
6298	Chr5	57770217
6299	Chr5	57770182
6300	Chr5	57855974
6301	Chr5	57855771
6302	Chr5	57860318
6303	Chr5	57860353
6304	Chr5	57871206
6305	Chr5	57871201
6306	Chr5	57904261
6307	Chr5	57953101
6308	Chr5	57976964
6309	Chr5	57976990
6310	Chr5	57977005
6311	Chr5	57990032
6312	Chr5	58010528
6313	Chr5	58097876
6314	Chr5	58097854
6315	Chr5	58149447
6316	Chr5	58334822
6317	Chr5	58334824
6318	Chr5	58401868
6319	Chr5	58428801
6320	Chr5	58428542
6321	Chr5	58428505
6322	Chr5	58458854
6323	Chr5	58471693
6324	Chr5	58512142
6325	Chr5	58511826
6326	Chr5	58511822
6327	Chr5	58534308
6328	Chr5	58737113
6329	Chr5	58960969
6330	Chr5	58960914
6331	Chr5	59082473
6332	Chr5	59094851
6333	Chr5	59096388
6334	Chr5	59096043
6335	Chr5	59183811
6336	Chr5	59186660
6337	Chr5	59186404
6338	Chr5	59186368
6339	Chr5	59195163
6340	Chr5	59206476
6341	Chr5	59206394
6342	Chr5	59206087

6343	Chr5	59258257
6344	Chr5	59261759
6345	Chr5	59280789
6346	Chr5	59314547
6347	Chr5	59314538
6348	Chr5	59314502
6349	Chr5	59946431
6350	Chr5	60176963
6351	Chr5	60315908
6352	Chr5	62254936
6353	Chr5	63908820
6354	Chr5	63915978
6355	Chr6	13916
6356	Chr6	13971
6357	Chr6	47838
6358	Chr6	47869
6359	Chr6	47877
6360	Chr6	47899
6361	Chr6	47911
6362	Chr6	47916
6363	Chr6	48190
6364	Chr6	47785
6365	Chr6	47755
6366	Chr6	47577
6367	Chr6	47574
6368	Chr6	47571
6369	Chr6	47560
6370	Chr6	47539
6371	Chr6	47535
6372	Chr6	302634
6373	Chr6	302622
6374	Chr6	302307
6375	Chr6	302232
6376	Chr6	333101
6377	Chr6	333133
6378	Chr6	333185
6379	Chr6	333392
6380	Chr6	333393
6381	Chr6	333396
6382	Chr6	333397
6383	Chr6	353898
6384	Chr6	353893
6385	Chr6	353877
6386	Chr6	353852
6387	Chr6	353809
6388	Chr6	355596
6389	Chr6	506993

6390	Chr6	506960
6391	Chr6	615383
6392	Chr6	636644
6393	Chr6	656706
6394	Chr6	656709
6395	Chr6	656755
6396	Chr6	657045
6397	Chr6	657075
6398	Chr6	657100
6399	Chr6	657122
6400	Chr6	657131
6401	Chr6	657138
6402	Chr6	752185
6403	Chr6	752164
6404	Chr6	752118
6405	Chr6	752092
6406	Chr6	751723
6407	Chr6	751703
6408	Chr6	780499
6409	Chr6	780752
6410	Chr6	807875
6411	Chr6	807779
6412	Chr6	820564
6413	Chr6	820359
6414	Chr6	820291
6415	Chr6	889894
6416	Chr6	889990
6417	Chr6	889837
6418	Chr6	889808
6419	Chr6	889788
6420	Chr6	889544
6421	Chr6	889537
6422	Chr6	889535
6423	Chr6	889509
6424	Chr6	889503
6425	Chr6	889458
6426	Chr6	893316
6427	Chr6	893016
6428	Chr6	1075353
6429	Chr6	1075359
6430	Chr6	1075413
6431	Chr6	1075544
6432	Chr6	1075577
6433	Chr6	1204531
6434	Chr6	1204542
6435	Chr6	1204585
6436	Chr6	1204588

6437	Chr6	1204800
6438	Chr6	1204801
6439	Chr6	1204826
6440	Chr6	1204847
6441	Chr6	1204854
6442	Chr6	1204857
6443	Chr6	1204858
6444	Chr6	1204878
6445	Chr6	1204885
6446	Chr6	1204889
6447	Chr6	1221410
6448	Chr6	1221385
6449	Chr6	1221369
6450	Chr6	1221364
6451	Chr6	1221346
6452	Chr6	1221344
6453	Chr6	1221339
6454	Chr6	1273568
6455	Chr6	1273564
6456	Chr6	1273276
6457	Chr6	1331528
6458	Chr6	1331527
6459	Chr6	1331494
6460	Chr6	1331493
6461	Chr6	1331471
6462	Chr6	1331158
6463	Chr6	1391907
6464	Chr6	1391950
6465	Chr6	1391986
6466	Chr6	1392310
6467	Chr6	1510940
6468	Chr6	1510970
6469	Chr6	1510972
6470	Chr6	1510976
6471	Chr6	1510988
6472	Chr6	1510994
6473	Chr6	1511007
6474	Chr6	1511159
6475	Chr6	1511164
6476	Chr6	1511197
6477	Chr6	1516542
6478	Chr6	1519784
6479	Chr6	1519528
6480	Chr6	1519484
6481	Chr6	1524106
6482	Chr6	1524067
6483	Chr6	1524036

6484	Chr6	1524011
6485	Chr6	1523713
6486	Chr6	1664338
6487	Chr6	1664254
6488	Chr6	1664167
6489	Chr6	1664038
6490	Chr6	1664009
6491	Chr6	1678800
6492	Chr6	1897681
6493	Chr6	1897673
6494	Chr6	1897655
6495	Chr6	1897633
6496	Chr6	1953194
6497	Chr6	1953167
6498	Chr6	1953166
6499	Chr6	1952906
6500	Chr6	1968830
6501	Chr6	1968821
6502	Chr6	1968782
6503	Chr6	1968748
6504	Chr6	1968605
6505	Chr6	1993505
6506	Chr6	1993487
6507	Chr6	1993481
6508	Chr6	1993457
6509	Chr6	1994210
6510	Chr6	1994212
6511	Chr6	2098329
6512	Chr6	2098312
6513	Chr6	2098128
6514	Chr6	2098115
6515	Chr6	2158584
6516	Chr6	2184491
6517	Chr6	2184454
6518	Chr6	2184450
6519	Chr6	2184448
6520	Chr6	2184436
6521	Chr6	2184096
6522	Chr6	2336858
6523	Chr6	2336876
6524	Chr6	2336879
6525	Chr6	2336883
6526	Chr6	2336913
6527	Chr6	2336922
6528	Chr6	2337217
6529	Chr6	2337223
6530	Chr6	2337230

6531	Chr6	2337260
6532	Chr6	2394390
6533	Chr6	2443966
6534	Chr6	2444003
6535	Chr6	2444033
6536	Chr6	2444205
6537	Chr6	2444219
6538	Chr6	2444221
6539	Chr6	2444244
6540	Chr6	2444257
6541	Chr6	2444260
6542	Chr6	2592266
6543	Chr6	2592318
6544	Chr6	2592331
6545	Chr6	2592347
6546	Chr6	2634063
6547	Chr6	2634082
6548	Chr6	2634109
6549	Chr6	2634141
6550	Chr6	2633965
6551	Chr6	2664427
6552	Chr6	2664474
6553	Chr6	2664485
6554	Chr6	2664496
6555	Chr6	2812373
6556	Chr6	2812355
6557	Chr6	2812324
6558	Chr6	3096166
6559	Chr6	3096210
6560	Chr6	3162620
6561	Chr6	3162269
6562	Chr6	3162258
6563	Chr6	3162218
6564	Chr6	3269595
6565	Chr6	3377247
6566	Chr6	3377606
6567	Chr6	3377653
6568	Chr6	3425599
6569	Chr6	3425677
6570	Chr6	3425824
6571	Chr6	3425827
6572	Chr6	3425853
6573	Chr6	3425855
6574	Chr6	3425913
6575	Chr6	3425566
6576	Chr6	3425515
6577	Chr6	3425136

6578	Chr6	3427577
6579	Chr6	3427553
6580	Chr6	3504596
6581	Chr6	3504526
6582	Chr6	3504494
6583	Chr6	3504310
6584	Chr6	3504301
6585	Chr6	3504239
6586	Chr6	3504225
6587	Chr6	3572317
6588	Chr6	3594709
6589	Chr6	3594684
6590	Chr6	3594432
6591	Chr6	3594393
6592	Chr6	3708789
6593	Chr6	3708807
6594	Chr6	3708814
6595	Chr6	3880010
6596	Chr6	3880073
6597	Chr6	3880074
6598	Chr6	3880274
6599	Chr6	3880282
6600	Chr6	3880307
6601	Chr6	4078157
6602	Chr6	4078169
6603	Chr6	4078172
6604	Chr6	4109754
6605	Chr6	4109790
6606	Chr6	4340489
6607	Chr6	4340805
6608	Chr6	4340812
6609	Chr6	4340826
6610	Chr6	4340850
6611	Chr6	4340409
6612	Chr6	4340196
6613	Chr6	4340181
6614	Chr6	4374423
6615	Chr6	4374449
6616	Chr6	4374468
6617	Chr6	4374477
6618	Chr6	4374731
6619	Chr6	4566234
6620	Chr6	4566233
6621	Chr6	4566176
6622	Chr6	4566174
6623	Chr6	4566161
6624	Chr6	4565976

6625	Chr6	4565949
6626	Chr6	4565944
6627	Chr6	4565937
6628	Chr6	4565931
6629	Chr6	4565930
6630	Chr6	4565918
6631	Chr6	4565902
6632	Chr6	4565895
6633	Chr6	4565890
6634	Chr6	4565881
6635	Chr6	4591695
6636	Chr6	4591656
6637	Chr6	4591647
6638	Chr6	4591637
6639	Chr6	4591393
6640	Chr6	4591338
6641	Chr6	4591313
6642	Chr6	4612173
6643	Chr6	4612170
6644	Chr6	4611776
6645	Chr6	4616770
6646	Chr6	4616795
6647	Chr6	4616808
6648	Chr6	4616987
6649	Chr6	4617006
6650	Chr6	4617072
6651	Chr6	4625217
6652	Chr6	4625189
6653	Chr6	4625172
6654	Chr6	4625147
6655	Chr6	4624802
6656	Chr6	4690947
6657	Chr6	4690623
6658	Chr6	4690610
6659	Chr6	4691312
6660	Chr6	4770071
6661	Chr6	4770059
6662	Chr6	4770042
6663	Chr6	4770040
6664	Chr6	4769715
6665	Chr6	4769708
6666	Chr6	4796759
6667	Chr6	4796717
6668	Chr6	4796706
6669	Chr6	4796441
6670	Chr6	4796426
6671	Chr6	4796388

6672	Chr6	4796371
6673	Chr6	4865172
6674	Chr6	4865219
6675	Chr6	4865228
6676	Chr6	4865249
6677	Chr6	4865250
6678	Chr6	4876267
6679	Chr6	4876316
6680	Chr6	4876560
6681	Chr6	4876569
6682	Chr6	4876623
6683	Chr6	4876631
6684	Chr6	4876205
6685	Chr6	4876155
6686	Chr6	4958962
6687	Chr6	4958964
6688	Chr6	4959164
6689	Chr6	5001044
6690	Chr6	5001049
6691	Chr6	5001055
6692	Chr6	5001089
6693	Chr6	5001097
6694	Chr6	5001100
6695	Chr6	5001125
6696	Chr6	5001132
6697	Chr6	5218519
6698	Chr6	5218562
6699	Chr6	5218840
6700	Chr6	5218850
6701	Chr6	5218866
6702	Chr6	5228668
6703	Chr6	5228669
6704	Chr6	5228678
6705	Chr6	5228680
6706	Chr6	5228722
6707	Chr6	5228920
6708	Chr6	5228929
6709	Chr6	5228956
6710	Chr6	5228959
6711	Chr6	5228967
6712	Chr6	5228971
6713	Chr6	5228983
6714	Chr6	5228999
6715	Chr6	5229012
6716	Chr6	5286593
6717	Chr6	5286564
6718	Chr6	5286557

6719	Chr6	5286284
6720	Chr6	5286243
6721	Chr6	5286234
6722	Chr6	5286212
6723	Chr6	5340528
6724	Chr6	5340527
6725	Chr6	5340479
6726	Chr6	5340293
6727	Chr6	5340285
6728	Chr6	5340283
6729	Chr6	5340247
6730	Chr6	5340243
6731	Chr6	5340204
6732	Chr6	5378051
6733	Chr6	5377985
6734	Chr6	5377679
6735	Chr6	5377614
6736	Chr6	5420826
6737	Chr6	5421186
6738	Chr6	5420695
6739	Chr6	5420451
6740	Chr6	5461014
6741	Chr6	5461006
6742	Chr6	5460719
6743	Chr6	5460693
6744	Chr6	5461753
6745	Chr6	5461738
6746	Chr6	5461436
6747	Chr6	5493741
6748	Chr6	5493774
6749	Chr6	5493818
6750	Chr6	5493950
6751	Chr6	5493980
6752	Chr6	5493981
6753	Chr6	5494001
6754	Chr6	5494014
6755	Chr6	5584233
6756	Chr6	5584259
6757	Chr6	5584291
6758	Chr6	5584334
6759	Chr6	5584550
6760	Chr6	5595924
6761	Chr6	5857392
6762	Chr6	5857421
6763	Chr6	5909418
6764	Chr6	5909361
6765	Chr6	5909198

6766	Chr6	5909168
6767	Chr6	6007158
6768	Chr6	6007177
6769	Chr6	6007191
6770	Chr6	6007368
6771	Chr6	6007405
6772	Chr6	6007428
6773	Chr6	6007460
6774	Chr6	6007467
6775	Chr6	6050745
6776	Chr6	6050742
6777	Chr6	6050736
6778	Chr6	6050732
6779	Chr6	6050719
6780	Chr6	6050718
6781	Chr6	6050714
6782	Chr6	6050699
6783	Chr6	6050505
6784	Chr6	6050462
6785	Chr6	6050458
6786	Chr6	6050448
6787	Chr6	6050444
6788	Chr6	6050434
6789	Chr6	6050412
6790	Chr6	6050405
6791	Chr6	6113203
6792	Chr6	6113174
6793	Chr6	6113145
6794	Chr6	6113131
6795	Chr6	6158757
6796	Chr6	6158718
6797	Chr6	6158708
6798	Chr6	6158574
6799	Chr6	6158563
6800	Chr6	6158542
6801	Chr6	6158529
6802	Chr6	6158519
6803	Chr6	6158481
6804	Chr6	6251848
6805	Chr6	6251866
6806	Chr6	6252171
6807	Chr6	6352239
6808	Chr6	6352252
6809	Chr6	6352279
6810	Chr6	6352290
6811	Chr6	6352298
6812	Chr6	6352319

6813	Chr6	6352497
6814	Chr6	6352508
6815	Chr6	6352520
6816	Chr6	6352525
6817	Chr6	6352526
6818	Chr6	6352532
6819	Chr6	6352541
6820	Chr6	6352552
6821	Chr6	6352554
6822	Chr6	6391153
6823	Chr6	6391216
6824	Chr6	6451499
6825	Chr6	6451497
6826	Chr6	6451491
6827	Chr6	6451433
6828	Chr6	6527807
6829	Chr6	6575551
6830	Chr6	6575556
6831	Chr6	6575558
6832	Chr6	6575589
6833	Chr6	6575606
6834	Chr6	6575615
6835	Chr6	6575642
6836	Chr6	6575882
6837	Chr6	6575894
6838	Chr6	6575901
6839	Chr6	6604019
6840	Chr6	6603943
6841	Chr6	6603658
6842	Chr6	6603610
6843	Chr6	6603597
6844	Chr6	6603592
6845	Chr6	6603585
6846	Chr6	6603579
6847	Chr6	6603576
6848	Chr6	6603560
6849	Chr6	6720403
6850	Chr6	6720500
6851	Chr6	6720621
6852	Chr6	6720703
6853	Chr6	6720705
6854	Chr6	6720724
6855	Chr6	6720337
6856	Chr6	6720295
6857	Chr6	6720056
6858	Chr6	6720040
6859	Chr6	6720029

6860	Chr6	6719996
6861	Chr6	6729685
6862	Chr6	6729648
6863	Chr6	6729640
6864	Chr6	6729388
6865	Chr6	6729380
6866	Chr6	6729373
6867	Chr6	6782732
6868	Chr6	6782733
6869	Chr6	6782740
6870	Chr6	6782754
6871	Chr6	6790671
6872	Chr6	6790667
6873	Chr6	6790632
6874	Chr6	6790590
6875	Chr6	6790582
6876	Chr6	6790415
6877	Chr6	6790348
6878	Chr6	6790331
6879	Chr6	6869263
6880	Chr6	6869274
6881	Chr6	6869301
6882	Chr6	6869327
6883	Chr6	6869675
6884	Chr6	6869683
6885	Chr6	6905741
6886	Chr6	6906084
6887	Chr6	6906087
6888	Chr6	6906103
6889	Chr6	6906136
6890	Chr6	6906138
6891	Chr6	7025011
6892	Chr6	7025033
6893	Chr6	7025037
6894	Chr6	7025038
6895	Chr6	7025080
6896	Chr6	7025244
6897	Chr6	7025312
6898	Chr6	7025321
6899	Chr6	7140176
6900	Chr6	7140184
6901	Chr6	7140187
6902	Chr6	7140210
6903	Chr6	7140236
6904	Chr6	7140239
6905	Chr6	7140255
6906	Chr6	7140256

6907	Chr6	7140268
6908	Chr6	7228497
6909	Chr6	7228470
6910	Chr6	7228442
6911	Chr6	7228118
6912	Chr6	7228080
6913	Chr6	7373964
6914	Chr6	7373969
6915	Chr6	7373979
6916	Chr6	7374397
6917	Chr6	7374407
6918	Chr6	7374413
6919	Chr6	7374424
6920	Chr6	7389832
6921	Chr6	7395153
6922	Chr6	7395141
6923	Chr6	7395093
6924	Chr6	7395092
6925	Chr6	7395090
6926	Chr6	7395073
6927	Chr6	7395070
6928	Chr6	7394911
6929	Chr6	7394904
6930	Chr6	7394892
6931	Chr6	7394867
6932	Chr6	7394854
6933	Chr6	7394835
6934	Chr6	7394823
6935	Chr6	7394820
6936	Chr6	7394818
6937	Chr6	7433823
6938	Chr6	7434058
6939	Chr6	7434065
6940	Chr6	7434080
6941	Chr6	7434087
6942	Chr6	7434101
6943	Chr6	7434107
6944	Chr6	7434122
6945	Chr6	7434129
6946	Chr6	7441542
6947	Chr6	7441518
6948	Chr6	7441474
6949	Chr6	7441472
6950	Chr6	7441461
6951	Chr6	7441459
6952	Chr6	7441143
6953	Chr6	7487607

6954	Chr6	7754527
6955	Chr6	7754538
6956	Chr6	7754548
6957	Chr6	7754551
6958	Chr6	7754571
6959	Chr6	7754578
6960	Chr6	7754585
6961	Chr6	7754605
6962	Chr6	7754608
6963	Chr6	7754612
6964	Chr6	7754939
6965	Chr6	7754972
6966	Chr6	7754980
6967	Chr6	7755012
6968	Chr6	7814592
6969	Chr6	7814593
6970	Chr6	7814598
6971	Chr6	7814636
6972	Chr6	7814648
6973	Chr6	7814851
6974	Chr6	7814853
6975	Chr6	7814857
6976	Chr6	7814859
6977	Chr6	7814907
6978	Chr6	7815907
6979	Chr6	7815908
6980	Chr6	7815915
6981	Chr6	7815922
6982	Chr6	7815983
6983	Chr6	7816256
6984	Chr6	7816267
6985	Chr6	7816287
6986	Chr6	7816296
6987	Chr6	7816311
6988	Chr6	7816324
6989	Chr6	7825922
6990	Chr6	7825926
6991	Chr6	7825927
6992	Chr6	7825937
6993	Chr6	7825947
6994	Chr6	7825951
6995	Chr6	7825967
6996	Chr6	7825973
6997	Chr6	7825977
6998	Chr6	7825981
6999	Chr6	7825985
7000	Chr6	7825988

7001	Chr6	7825989
7002	Chr6	7826005
7003	Chr6	7829571
7004	Chr6	7829598
7005	Chr6	7829609
7006	Chr6	7829808
7007	Chr6	7829809
7008	Chr6	7829866
7009	Chr6	7833695
7010	Chr6	7833693
7011	Chr6	7833658
7012	Chr6	7833627
7013	Chr6	7833385
7014	Chr6	7833361
7015	Chr6	7833323
7016	Chr6	7833321
7017	Chr6	7836185
7018	Chr6	7836174
7019	Chr6	7836153
7020	Chr6	7836140
7021	Chr6	7836131
7022	Chr6	7836128
7023	Chr6	7836111
7024	Chr6	7835932
7025	Chr6	7835930
7026	Chr6	7835925
7027	Chr6	7835896
7028	Chr6	7835864
7029	Chr6	7840266
7030	Chr6	7840321
7031	Chr6	7840325
7032	Chr6	7840331
7033	Chr6	7840333
7034	Chr6	7840559
7035	Chr6	7840565
7036	Chr6	7840567
7037	Chr6	7840573
7038	Chr6	7840606
7039	Chr6	7840631
7040	Chr6	7840633
7041	Chr6	7840641
7042	Chr6	7840233
7043	Chr6	7840231
7044	Chr6	7840222
7045	Chr6	7840206
7046	Chr6	7840197
7047	Chr6	7840161

7048	Chr6	7839971
7049	Chr6	7839969
7050	Chr6	7839939
7051	Chr6	7839922
7052	Chr6	7861667
7053	Chr6	7861640
7054	Chr6	7861639
7055	Chr6	7861630
7056	Chr6	7861629
7057	Chr6	7861621
7058	Chr6	7861612
7059	Chr6	7861601
7060	Chr6	7861453
7061	Chr6	7861447
7062	Chr6	7861433
7063	Chr6	7861425
7064	Chr6	7861412
7065	Chr6	7861405
7066	Chr6	7861398
7067	Chr6	7863099
7068	Chr6	7863104
7069	Chr6	7863139
7070	Chr6	7863157
7071	Chr6	7863393
7072	Chr6	7863399
7073	Chr6	7863400
7074	Chr6	7863428
7075	Chr6	7863463
7076	Chr6	7863467
7077	Chr6	7863496
7078	Chr6	7871415
7079	Chr6	7871412
7080	Chr6	7871410
7081	Chr6	7871408
7082	Chr6	7871404
7083	Chr6	7871396
7084	Chr6	7871393
7085	Chr6	7871364
7086	Chr6	7871362
7087	Chr6	7871341
7088	Chr6	7871326
7089	Chr6	7871132
7090	Chr6	7871121
7091	Chr6	7871115
7092	Chr6	7871070
7093	Chr6	7871068
7094	Chr6	7871056

7095	Chr6	7871053
7096	Chr6	7871052
7097	Chr6	7873086
7098	Chr6	7873071
7099	Chr6	7873066
7100	Chr6	7872984
7101	Chr6	7872802
7102	Chr6	7872788
7103	Chr6	7872768
7104	Chr6	7872740
7105	Chr6	7872726
7106	Chr6	7872706
7107	Chr6	7885199
7108	Chr6	7885214
7109	Chr6	7885217
7110	Chr6	7885242
7111	Chr6	7885247
7112	Chr6	7885360
7113	Chr6	7885377
7114	Chr6	7885396
7115	Chr6	7885401
7116	Chr6	7885403
7117	Chr6	7885413
7118	Chr6	7929858
7119	Chr6	7929867
7120	Chr6	7929870
7121	Chr6	7929886
7122	Chr6	7929897
7123	Chr6	7930212
7124	Chr6	7930311
7125	Chr6	7932745
7126	Chr6	7932950
7127	Chr6	7932975
7128	Chr6	7933000
7129	Chr6	7959975
7130	Chr6	7959992
7131	Chr6	7960018
7132	Chr6	7960043
7133	Chr6	7960045
7134	Chr6	7960367
7135	Chr6	7960371
7136	Chr6	7960415
7137	Chr6	7960432
7138	Chr6	7965637
7139	Chr6	7965656
7140	Chr6	7965661
7141	Chr6	7965669

7142	Chr6	7965695
7143	Chr6	7965981
7144	Chr6	7965982
7145	Chr6	7966015
7146	Chr6	7966020
7147	Chr6	7966071
7148	Chr6	7986452
7149	Chr6	7986757
7150	Chr6	7986761
7151	Chr6	7986817
7152	Chr6	8058505
7153	Chr6	8058536
7154	Chr6	8058574
7155	Chr6	8058589
7156	Chr6	8058817
7157	Chr6	8058827
7158	Chr6	8058830
7159	Chr6	8058844
7160	Chr6	8058893
7161	Chr6	8179139
7162	Chr6	8179156
7163	Chr6	8179431
7164	Chr6	8179433
7165	Chr6	8179464
7166	Chr6	8179474
7167	Chr6	8179476
7168	Chr6	8179485
7169	Chr6	8318950
7170	Chr6	8318898
7171	Chr6	8318875
7172	Chr6	8318571
7173	Chr6	8402818
7174	Chr6	8537389
7175	Chr6	8563609
7176	Chr6	12408224
7177	Chr6	12427565
7178	Chr6	12427564
7179	Chr6	12427558
7180	Chr6	12427557
7181	Chr6	12427544
7182	Chr6	12427525
7183	Chr6	12427521
7184	Chr6	12427517
7185	Chr6	12427505
7186	Chr6	12427496
7187	Chr6	12427483
7188	Chr6	12440410

7189	Chr6	12440426
7190	Chr6	12440432
7191	Chr6	12440437
7192	Chr6	12440444
7193	Chr6	12440464
7194	Chr6	12440478
7195	Chr6	12440487
7196	Chr6	12440499
7197	Chr6	12440508
7198	Chr6	12440510
7199	Chr6	12440733
7200	Chr6	12440737
7201	Chr6	12440743
7202	Chr6	12440747
7203	Chr6	12440750
7204	Chr6	12461927
7205	Chr6	12461940
7206	Chr6	12483838
7207	Chr6	12521068
7208	Chr6	12521075
7209	Chr6	12521084
7210	Chr6	12534547
7211	Chr6	13036617
7212	Chr6	13041974
7213	Chr6	13042007
7214	Chr6	13042260
7215	Chr6	13047087
7216	Chr6	13047059
7217	Chr6	13047056
7218	Chr6	13047033
7219	Chr6	13047026
7220	Chr6	13047023
7221	Chr6	13079855
7222	Chr6	13080168
7223	Chr6	13080136
7224	Chr6	13080135
7225	Chr6	13080134
7226	Chr6	13080117
7227	Chr6	13080112
7228	Chr6	13080111
7229	Chr6	13080103
7230	Chr6	13080094
7231	Chr6	13080091
7232	Chr6	13080081
7233	Chr6	13080072
7234	Chr6	13079853
7235	Chr6	13079845

7236	Chr6	13079833
7237	Chr6	13079819
7238	Chr6	13079803
7239	Chr6	13137372
7240	Chr6	13190837
7241	Chr6	13190787
7242	Chr6	13190785
7243	Chr6	13190771
7244	Chr6	13190770
7245	Chr6	13190553
7246	Chr6	13249216
7247	Chr6	13249287
7248	Chr6	13266364
7249	Chr6	13266339
7250	Chr6	13266310
7251	Chr6	13266089
7252	Chr6	13266081
7253	Chr6	13266064
7254	Chr6	13266060
7255	Chr6	13266005
7256	Chr6	13265989
7257	Chr6	13294805
7258	Chr6	13294952
7259	Chr6	13294973
7260	Chr6	13294990
7261	Chr6	13295050
7262	Chr6	13357603
7263	Chr6	13399525
7264	Chr6	13399592
7265	Chr6	13399614
7266	Chr6	13407524
7267	Chr6	13407521
7268	Chr6	13407514
7269	Chr6	13407502
7270	Chr6	13407462
7271	Chr6	13407162
7272	Chr6	13407151
7273	Chr6	13407149
7274	Chr6	13407130
7275	Chr6	13407103
7276	Chr6	13477518
7277	Chr6	13477494
7278	Chr6	13477312
7279	Chr6	13477290
7280	Chr6	13477260
7281	Chr6	13477243
7282	Chr6	13477223

7283	Chr6	13585958
7284	Chr6	13585967
7285	Chr6	13585968
7286	Chr6	13585974
7287	Chr6	13585979
7288	Chr6	13585988
7289	Chr6	13586005
7290	Chr6	13586029
7291	Chr6	13586035
7292	Chr6	13586037
7293	Chr6	13586039
7294	Chr6	13586255
7295	Chr6	13586279
7296	Chr6	13586314
7297	Chr6	13586330
7298	Chr6	13586342
7299	Chr6	13586355
7300	Chr6	13586357
7301	Chr6	13607637
7302	Chr6	13607649
7303	Chr6	13607695
7304	Chr6	13607845
7305	Chr6	13607865
7306	Chr6	13607882
7307	Chr6	13607887
7308	Chr6	13607900
7309	Chr6	13607919
7310	Chr6	13618680
7311	Chr6	13618632
7312	Chr6	13618626
7313	Chr6	13618376
7314	Chr6	13618369
7315	Chr6	13618367
7316	Chr6	13618354
7317	Chr6	13618347
7318	Chr6	13618342
7319	Chr6	13618324
7320	Chr6	13618322
7321	Chr6	13618318
7322	Chr6	13618315
7323	Chr6	13625154
7324	Chr6	13625156
7325	Chr6	13865766
7326	Chr6	13865722
7327	Chr6	13865691
7328	Chr6	13865562
7329	Chr6	13865542

7330	Chr6	13865540
7331	Chr6	13865538
7332	Chr6	13865531
7333	Chr6	13865519
7334	Chr6	13865516
7335	Chr6	13865498
7336	Chr6	13865484
7337	Chr6	13865461
7338	Chr6	13888847
7339	Chr6	13888863
7340	Chr6	13888870
7341	Chr6	13888871
7342	Chr6	13888872
7343	Chr6	13888882
7344	Chr6	13888884
7345	Chr6	13888901
7346	Chr6	13888902
7347	Chr6	13888912
7348	Chr6	13888919
7349	Chr6	13888922
7350	Chr6	13888927
7351	Chr6	13888930
7352	Chr6	13889149
7353	Chr6	13889162
7354	Chr6	13889166
7355	Chr6	13889171
7356	Chr6	13889198
7357	Chr6	13889216
7358	Chr6	13891547
7359	Chr6	13891514
7360	Chr6	13891225
7361	Chr6	13891216
7362	Chr6	13891214
7363	Chr6	13891182
7364	Chr6	13891179
7365	Chr6	13891170
7366	Chr6	13891157
7367	Chr6	13891150
7368	Chr6	14211040
7369	Chr6	14211050
7370	Chr6	14211064
7371	Chr6	14211114
7372	Chr6	14211140
7373	Chr6	14211370
7374	Chr6	14211376
7375	Chr6	14211382
7376	Chr6	14211408

7377	Chr6	14211412
7378	Chr6	14211420
7379	Chr6	14211433
7380	Chr6	14237671
7381	Chr6	14237678
7382	Chr6	14237682
7383	Chr6	14237692
7384	Chr6	14237711
7385	Chr6	14238006
7386	Chr6	14336055
7387	Chr6	14336058
7388	Chr6	14336081
7389	Chr6	14336082
7390	Chr6	14336102
7391	Chr6	14336126
7392	Chr6	14336456
7393	Chr6	14336476
7394	Chr6	14336522
7395	Chr6	14341288
7396	Chr6	14341320
7397	Chr6	14341347
7398	Chr6	14341359
7399	Chr6	14341637
7400	Chr6	14341654
7401	Chr6	14396513
7402	Chr6	14396528
7403	Chr6	14396553
7404	Chr6	14396565
7405	Chr6	14396834
7406	Chr6	14396859
7407	Chr6	14396874
7408	Chr6	14396877
7409	Chr6	14396902
7410	Chr6	14396922
7411	Chr6	14477230
7412	Chr6	14477223
7413	Chr6	14477013
7414	Chr6	14477009
7415	Chr6	14476938
7416	Chr6	14476930
7417	Chr6	14527427
7418	Chr6	14527429
7419	Chr6	14527443
7420	Chr6	14527445
7421	Chr6	14527464
7422	Chr6	14527483
7423	Chr6	14527485

7424	Chr6	14527496
7425	Chr6	14527506
7426	Chr6	14527508
7427	Chr6	14527731
7428	Chr6	14527734
7429	Chr6	14527749
7430	Chr6	14527756
7431	Chr6	14527773
7432	Chr6	14527778
7433	Chr6	14617725
7434	Chr6	14617929
7435	Chr6	14617949
7436	Chr6	14617964
7437	Chr6	14617979
7438	Chr6	14617982
7439	Chr6	14625922
7440	Chr6	14625917
7441	Chr6	14625881
7442	Chr6	14625877
7443	Chr6	14625717
7444	Chr6	14625693
7445	Chr6	14625686
7446	Chr6	14625678
7447	Chr6	14625649
7448	Chr6	14625642
7449	Chr6	14625639
7450	Chr6	15352955
7451	Chr6	15352966
7452	Chr6	15352967
7453	Chr6	15352969
7454	Chr6	15352973
7455	Chr6	15352974
7456	Chr6	15352976
7457	Chr6	15352982
7458	Chr6	15353001
7459	Chr6	15353218
7460	Chr6	15353222
7461	Chr6	15353275
7462	Chr6	15353278
7463	Chr6	15353279
7464	Chr6	15387873
7465	Chr6	15387876
7466	Chr6	15387889
7467	Chr6	15387901
7468	Chr6	15387932
7469	Chr6	15480352
7470	Chr6	15480325

7471	Chr6	15480314
7472	Chr6	15480309
7473	Chr6	15480296
7474	Chr6	15480283
7475	Chr6	15480275
7476	Chr6	15479971
7477	Chr6	15479958
7478	Chr6	15479951
7479	Chr6	15479932
7480	Chr6	15479919
7481	Chr6	15479904
7482	Chr6	15479901
7483	Chr6	15479897
7484	Chr6	15479886
7485	Chr6	15479881
7486	Chr6	15481183
7487	Chr6	15481216
7488	Chr6	15714142
7489	Chr6	15714113
7490	Chr6	15714103
7491	Chr6	15714101
7492	Chr6	15722878
7493	Chr6	15722911
7494	Chr6	15722913
7495	Chr6	15722940
7496	Chr6	15732072
7497	Chr6	15754988
7498	Chr6	15754702
7499	Chr6	15754670
7500	Chr6	15814433
7501	Chr6	15814377
7502	Chr6	15814362
7503	Chr6	15814115
7504	Chr6	15814103
7505	Chr6	15814102
7506	Chr6	15814101
7507	Chr6	15814088
7508	Chr6	16375464
7509	Chr6	16375481
7510	Chr6	16375490
7511	Chr6	16375518
7512	Chr6	16375556
7513	Chr6	16375712
7514	Chr6	16375731
7515	Chr6	16375736
7516	Chr6	16375747
7517	Chr6	16375774

7518	Chr6	16375781
7519	Chr6	16375787
7520	Chr6	16375788
7521	Chr6	16436006
7522	Chr6	16436093
7523	Chr6	16436840
7524	Chr6	16436859
7525	Chr6	16437243
7526	Chr6	16436495
7527	Chr6	16436426
7528	Chr6	16471443
7529	Chr6	16471479
7530	Chr6	16471512
7531	Chr6	16471817
7532	Chr6	16471824
7533	Chr6	16537356
7534	Chr6	16537359
7535	Chr6	16549363
7536	Chr6	16605731
7537	Chr6	16605759
7538	Chr6	16605962
7539	Chr6	16605999
7540	Chr6	16606029
7541	Chr6	16606030
7542	Chr6	17577756
7543	Chr6	17577716
7544	Chr6	17577699
7545	Chr6	17577684
7546	Chr6	17577682
7547	Chr6	17577501
7548	Chr6	17577500
7549	Chr6	17577494
7550	Chr6	17577470
7551	Chr6	17577467
7552	Chr6	17577465
7553	Chr6	17577439
7554	Chr6	17577430
7555	Chr6	17577426
7556	Chr6	17577424
7557	Chr6	17592658
7558	Chr6	17592673
7559	Chr6	17592681
7560	Chr6	17592707
7561	Chr6	17592708
7562	Chr6	17592711
7563	Chr6	17592712
7564	Chr6	17592722

7565	Chr6	17592725
7566	Chr6	17592760
7567	Chr6	17592910
7568	Chr6	17592932
7569	Chr6	17592942
7570	Chr6	17592943
7571	Chr6	17592996
7572	Chr6	17592998
7573	Chr6	17740256
7574	Chr6	17740254
7575	Chr6	17740251
7576	Chr6	17740241
7577	Chr6	17739924
7578	Chr6	17739919
7579	Chr6	17739889
7580	Chr6	17739887
7581	Chr6	17739835
7582	Chr6	17790070
7583	Chr6	17790095
7584	Chr6	17790104
7585	Chr6	17790118
7586	Chr6	17790119
7587	Chr6	17790120
7588	Chr6	17790127
7589	Chr6	17790130
7590	Chr6	17790149
7591	Chr6	17790155
7592	Chr6	17790169
7593	Chr6	17790437
7594	Chr6	17790442
7595	Chr6	17790443
7596	Chr6	17790502
7597	Chr6	17790510
7598	Chr6	17864758
7599	Chr6	17864753
7600	Chr6	17864752
7601	Chr6	17864571
7602	Chr6	17864557
7603	Chr6	17864553
7604	Chr6	17864529
7605	Chr6	17872203
7606	Chr6	17872195
7607	Chr6	17872159
7608	Chr6	18006070
7609	Chr6	18006087
7610	Chr6	18006121
7611	Chr6	18006299

7612	Chr6	18006350
7613	Chr6	18006360
7614	Chr6	18006361
7615	Chr6	18006370
7616	Chr6	18138281
7617	Chr6	18138286
7618	Chr6	18138299
7619	Chr6	18138340
7620	Chr6	18138531
7621	Chr6	18138553
7622	Chr6	18138025
7623	Chr6	18137962
7624	Chr6	18137955
7625	Chr6	18228520
7626	Chr6	18228529
7627	Chr6	18228541
7628	Chr6	18228592
7629	Chr6	18228608
7630	Chr6	18228622
7631	Chr6	18233721
7632	Chr6	18233689
7633	Chr6	18233370
7634	Chr6	18254422
7635	Chr6	18254412
7636	Chr6	18254375
7637	Chr6	18254372
7638	Chr6	18254371
7639	Chr6	18254362
7640	Chr6	18254341
7641	Chr6	18254318
7642	Chr6	18254178
7643	Chr6	18288611
7644	Chr6	18288921
7645	Chr6	18288936
7646	Chr6	18288947
7647	Chr6	18288952
7648	Chr6	18288976
7649	Chr6	18288981
7650	Chr6	18805218
7651	Chr6	18805221
7652	Chr6	18805237
7653	Chr6	18810840
7654	Chr6	18810657
7655	Chr6	18810620
7656	Chr6	18810584
7657	Chr6	19190299
7658	Chr6	19190227

7659	Chr6	19219748
7660	Chr6	19219731
7661	Chr6	19219728
7662	Chr6	19219681
7663	Chr6	19219661
7664	Chr6	19219481
7665	Chr6	19219455
7666	Chr6	19219405
7667	Chr6	19219404
7668	Chr6	19219390
7669	Chr6	19392823
7670	Chr6	19392824
7671	Chr6	19392835
7672	Chr6	19392864
7673	Chr6	19392896
7674	Chr6	19448190
7675	Chr6	19448184
7676	Chr6	19448117
7677	Chr6	19464760
7678	Chr6	19464769
7679	Chr6	19464771
7680	Chr6	19464779
7681	Chr6	19464782
7682	Chr6	19492567
7683	Chr6	19492557
7684	Chr6	19492534
7685	Chr6	19492527
7686	Chr6	19492509
7687	Chr6	19492265
7688	Chr6	19492213
7689	Chr6	19492211
7690	Chr6	19614200
7691	Chr6	19614222
7692	Chr6	19614291
7693	Chr6	19614472
7694	Chr6	19614487
7695	Chr6	19614493
7696	Chr6	19614497
7697	Chr6	19614509
7698	Chr6	19614513
7699	Chr6	19614171
7700	Chr6	19614157
7701	Chr6	19614152
7702	Chr6	19613884
7703	Chr6	19613868
7704	Chr6	19613857
7705	Chr6	19613831

7706	Chr6	19617528
7707	Chr6	19617560
7708	Chr6	19617567
7709	Chr6	19617569
7710	Chr6	19617580
7711	Chr6	19617599
7712	Chr6	19617905
7713	Chr6	19617915
7714	Chr6	19617938
7715	Chr6	19645699
7716	Chr6	19645364
7717	Chr6	19645358
7718	Chr6	19645318
7719	Chr6	19645314
7720	Chr6	19645305
7721	Chr6	19663727
7722	Chr6	19663754
7723	Chr6	19663821
7724	Chr6	19664059
7725	Chr6	19664075
7726	Chr6	19664136
7727	Chr6	19664147
7728	Chr6	19664148
7729	Chr6	19664186
7730	Chr6	19664189
7731	Chr6	19664405
7732	Chr6	19664419
7733	Chr6	19664428
7734	Chr6	19664429
7735	Chr6	19664463
7736	Chr6	19664488
7737	Chr6	19693973
7738	Chr6	19693967
7739	Chr6	19693956
7740	Chr6	19693955
7741	Chr6	19693912
7742	Chr6	19695927
7743	Chr6	19695953
7744	Chr6	19695966
7745	Chr6	19695976
7746	Chr6	19695985
7747	Chr6	19695987
7748	Chr6	19695994
7749	Chr6	19696313
7750	Chr6	19696380
7751	Chr6	19755954
7752	Chr6	19755955

7753	Chr6	19756012
7754	Chr6	19756020
7755	Chr6	19756031
7756	Chr6	19756134
7757	Chr6	19756170
7758	Chr6	19756176
7759	Chr6	19756187
7760	Chr6	19756210
7761	Chr6	19756237
7762	Chr6	19762358
7763	Chr6	19762363
7764	Chr6	19762365
7765	Chr6	19762392
7766	Chr6	19762395
7767	Chr6	19762397
7768	Chr6	19762404
7769	Chr6	19762411
7770	Chr6	19762415
7771	Chr6	19762425
7772	Chr6	19762432
7773	Chr6	19762460
7774	Chr6	19762576
7775	Chr6	19762594
7776	Chr6	19762603
7777	Chr6	19762635
7778	Chr6	19762650
7779	Chr6	19772857
7780	Chr6	19772864
7781	Chr6	19772865
7782	Chr6	19772871
7783	Chr6	19772896
7784	Chr6	19812461
7785	Chr6	19812454
7786	Chr6	19812424
7787	Chr6	19812387
7788	Chr6	19812382
7789	Chr6	19812112
7790	Chr6	19812106
7791	Chr6	19812103
7792	Chr6	19812092
7793	Chr6	19812055
7794	Chr6	19812038
7795	Chr6	19812028
7796	Chr6	19896940
7797	Chr6	19896994
7798	Chr6	19920868
7799	Chr6	19920505

7800	Chr6	19920453
7801	Chr6	19920424
7802	Chr6	19920419
7803	Chr6	19932052
7804	Chr6	19932053
7805	Chr6	19932118
7806	Chr6	19932149
7807	Chr6	19932272
7808	Chr6	19932284
7809	Chr6	19932323
7810	Chr6	19932040
7811	Chr6	19931752
7812	Chr6	19931653
7813	Chr6	19965946
7814	Chr6	19965952
7815	Chr6	19965968
7816	Chr6	19965970
7817	Chr6	19965971
7818	Chr6	19965984
7819	Chr6	19965992
7820	Chr6	19966301
7821	Chr6	19966305
7822	Chr6	19966351
7823	Chr6	19966381
7824	Chr6	19966391
7825	Chr6	20017399
7826	Chr6	20017425
7827	Chr6	20017434
7828	Chr6	20017716
7829	Chr6	20017732
7830	Chr6	20017777
7831	Chr6	20017809
7832	Chr6	20041173
7833	Chr6	20041174
7834	Chr6	20041189
7835	Chr6	20041213
7836	Chr6	20041251
7837	Chr6	20041265
7838	Chr6	20041270
7839	Chr6	20041449
7840	Chr6	20041475
7841	Chr6	20118197
7842	Chr6	20118167
7843	Chr6	20573742
7844	Chr6	20573734
7845	Chr6	20573711
7846	Chr6	20573568

7847	Chr6	20573555
7848	Chr6	20573491
7849	Chr6	20573469
7850	Chr6	20613973
7851	Chr6	20613980
7852	Chr6	20613997
7853	Chr6	20614050
7854	Chr6	20614291
7855	Chr6	20614319
7856	Chr6	20614350
7857	Chr6	20614351
7858	Chr6	20614359
7859	Chr6	20697535
7860	Chr6	20697520
7861	Chr6	20697508
7862	Chr6	20697343
7863	Chr6	20697335
7864	Chr6	20697293
7865	Chr6	20697290
7866	Chr6	20697252
7867	Chr6	20702684
7868	Chr6	20702751
7869	Chr6	20702948
7870	Chr6	20702954
7871	Chr6	20702990
7872	Chr6	20702992
7873	Chr6	20725670
7874	Chr6	20725626
7875	Chr6	20738910
7876	Chr6	20738913
7877	Chr6	20815540
7878	Chr6	20815507
7879	Chr6	20815480
7880	Chr6	20815477
7881	Chr6	20815323
7882	Chr6	20815322
7883	Chr6	20815275
7884	Chr6	20815256
7885	Chr6	20815250
7886	Chr6	20815246
7887	Chr6	20894634
7888	Chr6	21011842
7889	Chr6	21011820
7890	Chr6	21011801
7891	Chr6	21011790
7892	Chr6	21011787
7893	Chr6	21011770

7894	Chr6	21011542
7895	Chr6	21011538
7896	Chr6	21011535
7897	Chr6	21011513
7898	Chr6	21011506
7899	Chr6	21011499
7900	Chr6	21011492
7901	Chr6	21011488
7902	Chr6	21011481
7903	Chr6	21219933
7904	Chr6	21219929
7905	Chr6	21219921
7906	Chr6	21219890
7907	Chr6	21219882
7908	Chr6	21229363
7909	Chr6	21229361
7910	Chr6	21229354
7911	Chr6	21229341
7912	Chr6	21229340
7913	Chr6	21229333
7914	Chr6	21229332
7915	Chr6	21229301
7916	Chr6	21229062
7917	Chr6	21229055
7918	Chr6	21229023
7919	Chr6	21229018
7920	Chr6	21229016
7921	Chr6	21228989
7922	Chr6	21228972
7923	Chr6	21258662
7924	Chr6	21258619
7925	Chr6	21258600
7926	Chr6	21258581
7927	Chr6	21265647
7928	Chr6	21265613
7929	Chr6	21265607
7930	Chr6	21265606
7931	Chr6	21265592
7932	Chr6	21265546
7933	Chr6	21265376
7934	Chr6	21265372
7935	Chr6	21265326
7936	Chr6	21265324
7937	Chr6	21265323
7938	Chr6	21265310
7939	Chr6	21265302
7940	Chr6	21265300

7941	Chr6	21319949
7942	Chr6	21319947
7943	Chr6	21319918
7944	Chr6	21319901
7945	Chr6	21319882
7946	Chr6	21319870
7947	Chr6	21319671
7948	Chr6	21319642
7949	Chr6	21328996
7950	Chr6	21328972
7951	Chr6	21328697
7952	Chr6	21328670
7953	Chr6	21328667
7954	Chr6	21328652
7955	Chr6	21328651
7956	Chr6	21328644
7957	Chr6	21328636
7958	Chr6	21334416
7959	Chr6	21334361
7960	Chr6	21334180
7961	Chr6	21334174
7962	Chr6	21334171
7963	Chr6	21334168
7964	Chr6	21334129
7965	Chr6	21334127
7966	Chr6	21334126
7967	Chr6	21334113
7968	Chr6	21334105
7969	Chr6	21627132
7970	Chr6	21627112
7971	Chr6	22020838
7972	Chr6	22021270
7973	Chr6	22092953
7974	Chr6	22092905
7975	Chr6	22092899
7976	Chr6	22092894
7977	Chr6	22092590
7978	Chr6	22092585
7979	Chr6	22092563
7980	Chr6	22092562
7981	Chr6	22092561
7982	Chr6	22092558
7983	Chr6	22092527
7984	Chr6	22092515
7985	Chr6	22092510
7986	Chr6	22313068
7987	Chr6	22313060

7988	Chr6	22313058
7989	Chr6	22313050
7990	Chr6	22313028
7991	Chr6	22313021
7992	Chr6	22313003
7993	Chr6	22312991
7994	Chr6	22312968
7995	Chr6	22312821
7996	Chr6	22312818
7997	Chr6	22312816
7998	Chr6	22551980
7999	Chr6	22551978
8000	Chr6	22551972
8001	Chr6	22551932
8002	Chr6	22551880
8003	Chr6	22622596
8004	Chr6	22622643
8005	Chr6	22622653
8006	Chr6	22622888
8007	Chr6	22622925
8008	Chr6	22622959
8009	Chr6	22640486
8010	Chr6	22640447
8011	Chr6	22640444
8012	Chr6	22640430
8013	Chr6	22640429
8014	Chr6	22640413
8015	Chr6	22640410
8016	Chr6	22640243
8017	Chr6	22640200
8018	Chr6	22640180
8019	Chr6	22695196
8020	Chr6	22695190
8021	Chr6	22695185
8022	Chr6	22695184
8023	Chr6	22695168
8024	Chr6	22695158
8025	Chr6	22695157
8026	Chr6	22694883
8027	Chr6	22694873
8028	Chr6	22694859
8029	Chr6	22694801
8030	Chr6	22694800
8031	Chr6	22852643
8032	Chr6	22852626
8033	Chr6	22852624
8034	Chr6	22852619

8035	Chr6	22852612
8036	Chr6	22852608
8037	Chr6	22852604
8038	Chr6	22852591
8039	Chr6	22852579
8040	Chr6	22852570
8041	Chr6	22852244
8042	Chr6	22852239
8043	Chr6	22852223
8044	Chr6	22852222
8045	Chr6	22852201
8046	Chr6	22852182
8047	Chr6	22856521
8048	Chr6	22856523
8049	Chr6	22856524
8050	Chr6	22856527
8051	Chr6	22856543
8052	Chr6	22856555
8053	Chr6	22856568
8054	Chr6	22856580
8055	Chr6	22856595
8056	Chr6	22856598
8057	Chr6	22856611
8058	Chr6	22856813
8059	Chr6	22856817
8060	Chr6	22856822
8061	Chr6	22856835
8062	Chr6	22856839
8063	Chr6	22856856
8064	Chr6	22856877
8065	Chr6	22856879
8066	Chr6	22856887
8067	Chr6	22872325
8068	Chr6	22872312
8069	Chr6	22872308
8070	Chr6	22872277
8071	Chr6	22872267
8072	Chr6	22872257
8073	Chr6	22872086
8074	Chr6	22872072
8075	Chr6	22872070
8076	Chr6	22872068
8077	Chr6	22872065
8078	Chr6	22872064
8079	Chr6	22872047
8080	Chr6	22872034
8081	Chr6	22872017

8082	Chr6	22872015
8083	Chr6	22871993
8084	Chr6	22871991
8085	Chr6	23049143
8086	Chr6	23375554
8087	Chr6	23375493
8088	Chr6	23375473
8089	Chr6	23375471
8090	Chr6	23375462
8091	Chr6	23375294
8092	Chr6	23375269
8093	Chr6	23375258
8094	Chr6	23375247
8095	Chr6	23375228
8096	Chr6	23376196
8097	Chr6	23761383
8098	Chr6	23761667
8099	Chr6	23821213
8100	Chr6	23821243
8101	Chr6	23821265
8102	Chr6	23832067
8103	Chr6	23832069
8104	Chr6	23832071
8105	Chr6	23832076
8106	Chr6	23832100
8107	Chr6	23832101
8108	Chr6	23832117
8109	Chr6	23832122
8110	Chr6	23832140
8111	Chr6	23832376
8112	Chr6	24114739
8113	Chr6	24114726
8114	Chr6	24114488
8115	Chr6	24114429
8116	Chr6	24161967
8117	Chr6	24162002
8118	Chr6	24162048
8119	Chr6	24162059
8120	Chr6	24162312
8121	Chr6	24162357
8122	Chr6	24161893
8123	Chr6	24166136
8124	Chr6	24166169
8125	Chr6	24166229
8126	Chr6	24166422
8127	Chr6	24166430
8128	Chr6	24166455

8129	Chr6	24166460
8130	Chr6	24175472
8131	Chr6	24175479
8132	Chr6	24175785
8133	Chr6	24175791
8134	Chr6	24175878
8135	Chr6	24200808
8136	Chr6	24200926
8137	Chr6	24200980
8138	Chr6	24221934
8139	Chr6	24221956
8140	Chr6	24221990
8141	Chr6	24222223
8142	Chr6	24222288
8143	Chr6	24225306
8144	Chr6	24225299
8145	Chr6	24225279
8146	Chr6	24225264
8147	Chr6	24849333
8148	Chr6	24849339
8149	Chr6	24849345
8150	Chr6	24849360
8151	Chr6	25181815
8152	Chr6	25181830
8153	Chr6	25181840
8154	Chr6	25181851
8155	Chr6	25182036
8156	Chr6	25182083
8157	Chr6	25182093
8158	Chr6	25182107
8159	Chr6	25319543
8160	Chr6	25319577
8161	Chr6	25463160
8162	Chr6	25463350
8163	Chr6	25463436
8164	Chr6	25462815
8165	Chr6	25462789
8166	Chr6	25562275
8167	Chr6	25562329
8168	Chr6	25708946
8169	Chr6	25708930
8170	Chr6	25708914
8171	Chr6	25708900
8172	Chr6	25708851
8173	Chr6	25708579
8174	Chr6	25708572
8175	Chr6	25708546

8176	Chr6	25709881
8177	Chr6	25710178
8178	Chr6	25710182
8179	Chr6	25710193
8180	Chr6	25710206
8181	Chr6	25710238
8182	Chr6	25709766
8183	Chr6	25709736
8184	Chr6	25709711
8185	Chr6	25709707
8186	Chr6	25709700
8187	Chr6	25709529
8188	Chr6	25709520
8189	Chr6	25709487
8190	Chr6	25796346
8191	Chr6	25796334
8192	Chr6	25796330
8193	Chr6	25796294
8194	Chr6	25796262
8195	Chr6	25796105
8196	Chr6	25796085
8197	Chr6	25796013
8198	Chr6	25835847
8199	Chr6	25835861
8200	Chr6	25836062
8201	Chr6	25835724
8202	Chr6	25835722
8203	Chr6	25835719
8204	Chr6	25835693
8205	Chr6	25835453
8206	Chr6	25835452
8207	Chr6	25835448
8208	Chr6	25835440
8209	Chr6	25835438
8210	Chr6	25888356
8211	Chr6	25888339
8212	Chr6	25993819
8213	Chr6	25993887
8214	Chr6	25993776
8215	Chr6	25993732
8216	Chr6	25993680
8217	Chr6	26071388
8218	Chr6	26132018
8219	Chr6	26132004
8220	Chr6	26132002
8221	Chr6	26131734
8222	Chr6	26131717

8223	Chr6	26143517
8224	Chr6	26143536
8225	Chr6	26143596
8226	Chr6	26143598
8227	Chr6	26143930
8228	Chr6	26143964
8229	Chr6	26191839
8230	Chr6	26192046
8231	Chr6	26192058
8232	Chr6	26192109
8233	Chr6	26193017
8234	Chr6	26193012
8235	Chr6	26192950
8236	Chr6	26192688
8237	Chr6	26192672
8238	Chr6	26194381
8239	Chr6	26194424
8240	Chr6	26502937
8241	Chr6	26502939
8242	Chr6	26502965
8243	Chr6	26502971
8244	Chr6	26502975
8245	Chr6	26502979
8246	Chr6	26502986
8247	Chr6	26502987
8248	Chr6	26503241
8249	Chr6	26503260
8250	Chr6	26503266
8251	Chr6	26503296
8252	Chr6	26503304
8253	Chr6	26502861
8254	Chr6	26502843
8255	Chr6	26502829
8256	Chr6	26502816
8257	Chr6	26502591
8258	Chr6	26502573
8259	Chr6	26502568
8260	Chr6	26503702
8261	Chr6	26503677
8262	Chr6	26503648
8263	Chr6	26503639
8264	Chr6	26503634
8265	Chr6	26503626
8266	Chr6	26503402
8267	Chr6	26668843
8268	Chr6	26668854
8269	Chr6	26668878

8270	Chr6	26668927
8271	Chr6	26669075
8272	Chr6	26669077
8273	Chr6	26669085
8274	Chr6	26669104
8275	Chr6	26669136
8276	Chr6	26669141
8277	Chr6	26672052
8278	Chr6	26672069
8279	Chr6	26672083
8280	Chr6	26672094
8281	Chr6	26672105
8282	Chr6	26672114
8283	Chr6	26672119
8284	Chr6	26672133
8285	Chr6	26918370
8286	Chr6	26918314
8287	Chr6	26918170
8288	Chr6	26918151
8289	Chr6	26918149
8290	Chr6	26918139
8291	Chr6	26918125
8292	Chr6	26918120
8293	Chr6	26918117
8294	Chr6	26918100
8295	Chr6	26918099
8296	Chr6	27138061
8297	Chr6	27138065
8298	Chr6	27138091
8299	Chr6	27138099
8300	Chr6	27330080
8301	Chr6	27329887
8302	Chr6	27329873
8303	Chr6	27329871
8304	Chr6	27329852
8305	Chr6	27329799
8306	Chr6	27365952
8307	Chr6	27365964
8308	Chr6	27452104
8309	Chr6	27452113
8310	Chr6	27452115
8311	Chr6	27452124
8312	Chr6	27452132
8313	Chr6	27452135
8314	Chr6	27452136
8315	Chr6	27452141
8316	Chr6	27452143

8317	Chr6	27452164
8318	Chr6	27452172
8319	Chr6	27452206
8320	Chr6	27452382
8321	Chr6	27452401
8322	Chr6	27452404
8323	Chr6	27509890
8324	Chr6	27509875
8325	Chr6	27509874
8326	Chr6	27509854
8327	Chr6	27509828
8328	Chr6	27509823
8329	Chr6	27509821
8330	Chr6	27509792
8331	Chr6	27509544
8332	Chr6	27509521
8333	Chr6	27509520
8334	Chr6	27509513
8335	Chr6	27509497
8336	Chr6	27509468
8337	Chr6	27514623
8338	Chr6	27514627
8339	Chr6	27514629
8340	Chr6	27514632
8341	Chr6	27514654
8342	Chr6	27514681
8343	Chr6	27514682
8344	Chr6	27514706
8345	Chr6	27514869
8346	Chr6	27514895
8347	Chr6	27514901
8348	Chr6	27514911
8349	Chr6	27514935
8350	Chr6	27514599
8351	Chr6	27514591
8352	Chr6	27514554
8353	Chr6	27514548
8354	Chr6	27514507
8355	Chr6	27514386
8356	Chr6	27514355
8357	Chr6	27514351
8358	Chr6	27514323
8359	Chr6	27514316
8360	Chr6	27514296
8361	Chr6	27693386
8362	Chr6	27693393
8363	Chr6	27693455

8364	Chr6	27693613
8365	Chr6	27693615
8366	Chr6	27693635
8367	Chr6	27693641
8368	Chr6	27734631
8369	Chr6	27734636
8370	Chr6	27734657
8371	Chr6	27734658
8372	Chr6	27734667
8373	Chr6	27734670
8374	Chr6	27734677
8375	Chr6	27734684
8376	Chr6	27734687
8377	Chr6	27734690
8378	Chr6	27734720
8379	Chr6	27734871
8380	Chr6	27734885
8381	Chr6	27734894
8382	Chr6	27734905
8383	Chr6	27734906
8384	Chr6	27734909
8385	Chr6	27734917
8386	Chr6	27734926
8387	Chr6	27734951
8388	Chr6	27739790
8389	Chr6	27740078
8390	Chr6	27740102
8391	Chr6	27740104
8392	Chr6	27740125
8393	Chr6	27740162
8394	Chr6	27740165
8395	Chr6	27821233
8396	Chr6	27821260
8397	Chr6	27821266
8398	Chr6	27821309
8399	Chr6	27821310
8400	Chr6	27821315
8401	Chr6	27821539
8402	Chr6	27821570
8403	Chr6	27821579
8404	Chr6	27821217
8405	Chr6	27821188
8406	Chr6	27821145
8407	Chr6	27821141
8408	Chr6	27821123
8409	Chr6	27820845
8410	Chr6	27820844

8411	Chr6	27820822
8412	Chr6	27820820
8413	Chr6	27820782
8414	Chr6	27820770
8415	Chr6	27869894
8416	Chr6	27869913
8417	Chr6	27870185
8418	Chr6	27870191
8419	Chr6	27870203
8420	Chr6	27917283
8421	Chr6	27973214
8422	Chr6	27973243
8423	Chr6	27973251
8424	Chr6	27973280
8425	Chr6	27973287
8426	Chr6	27973298
8427	Chr6	28004836
8428	Chr6	28004863
8429	Chr6	28004892
8430	Chr6	28005079
8431	Chr6	28004767
8432	Chr6	28004752
8433	Chr6	28004727
8434	Chr6	28004721
8435	Chr6	28004717
8436	Chr6	28004436
8437	Chr6	28004405
8438	Chr6	28004403
8439	Chr6	28724578
8440	Chr6	28724580
8441	Chr6	28724590
8442	Chr6	28724604
8443	Chr6	28724617
8444	Chr6	28724628
8445	Chr6	28724662
8446	Chr6	28724664
8447	Chr6	28724905
8448	Chr6	28724918
8449	Chr6	28724924
8450	Chr6	28724926
8451	Chr6	28724932
8452	Chr6	28724934
8453	Chr6	28724946
8454	Chr6	28724949
8455	Chr6	28724953
8456	Chr6	28724986
8457	Chr6	28724999

8458	Chr6	28725003
8459	Chr6	28821291
8460	Chr6	28821293
8461	Chr6	28821302
8462	Chr6	28821315
8463	Chr6	28821322
8464	Chr6	28821329
8465	Chr6	28821342
8466	Chr6	28821344
8467	Chr6	28821355
8468	Chr6	28915391
8469	Chr6	28961464
8470	Chr6	28961466
8471	Chr6	28961472
8472	Chr6	28961474
8473	Chr6	28961483
8474	Chr6	28961492
8475	Chr6	28961505
8476	Chr6	28961521
8477	Chr6	28961525
8478	Chr6	28961536
8479	Chr6	28961744
8480	Chr6	28961753
8481	Chr6	28961790
8482	Chr6	28961805
8483	Chr6	28992407
8484	Chr6	28992435
8485	Chr6	28992444
8486	Chr6	28992457
8487	Chr6	28992581
8488	Chr6	28992592
8489	Chr6	28992593
8490	Chr6	28992652
8491	Chr6	29041153
8492	Chr6	29041126
8493	Chr6	29041125
8494	Chr6	29041109
8495	Chr6	29041108
8496	Chr6	29041095
8497	Chr6	29041088
8498	Chr6	29041058
8499	Chr6	29040856
8500	Chr6	29040828
8501	Chr6	29040799
8502	Chr6	29040790
8503	Chr6	29040784
8504	Chr6	29090180

8505	Chr6	29090215
8506	Chr6	29125893
8507	Chr6	29126135
8508	Chr6	29201891
8509	Chr6	29201898
8510	Chr6	29201915
8511	Chr6	29201920
8512	Chr6	29201947
8513	Chr6	29201955
8514	Chr6	29201963
8515	Chr6	29201965
8516	Chr6	29202095
8517	Chr6	29202097
8518	Chr6	29202099
8519	Chr6	29202106
8520	Chr6	29202116
8521	Chr6	29202128
8522	Chr6	29202147
8523	Chr6	29202149
8524	Chr6	29202171
8525	Chr6	29202175
8526	Chr6	29438733
8527	Chr6	29438713
8528	Chr6	29438701
8529	Chr6	29438667
8530	Chr6	29438399
8531	Chr6	29509469
8532	Chr6	29509475
8533	Chr6	29509493
8534	Chr6	29509518
8535	Chr6	29509522
8536	Chr6	29509531
8537	Chr6	29524938
8538	Chr6	29524936
8539	Chr6	29524932
8540	Chr6	29524908
8541	Chr6	29524899
8542	Chr6	29524877
8543	Chr6	29524872
8544	Chr6	29545080
8545	Chr6	29545070
8546	Chr6	29545035
8547	Chr6	29544865
8548	Chr6	29606740
8549	Chr6	29606758
8550	Chr6	29606762
8551	Chr6	29606764

8552	Chr6	29606780
8553	Chr6	29606785
8554	Chr6	29606786
8555	Chr6	29606793
8556	Chr6	29607013
8557	Chr6	29607032
8558	Chr6	29607034
8559	Chr6	29607036
8560	Chr6	29607053
8561	Chr6	29607054
8562	Chr6	29607062
8563	Chr6	29641411
8564	Chr6	29641421
8565	Chr6	29641448
8566	Chr6	29641683
8567	Chr6	29641694
8568	Chr6	29641721
8569	Chr6	29641741
8570	Chr6	29641743
8571	Chr6	29641752
8572	Chr6	29643939
8573	Chr6	29670662
8574	Chr6	29670681
8575	Chr6	29670686
8576	Chr6	29670725
8577	Chr6	29707466
8578	Chr6	29716430
8579	Chr6	29716449
8580	Chr6	29716655
8581	Chr6	29716656
8582	Chr6	29716708
8583	Chr6	29716718
8584	Chr6	29716748
8585	Chr6	29716346
8586	Chr6	29716315
8587	Chr6	29716287
8588	Chr6	29715995
8589	Chr6	29790209
8590	Chr6	29790229
8591	Chr6	29790298
8592	Chr6	29790538
8593	Chr6	29790542
8594	Chr6	29790588
8595	Chr6	29925160
8596	Chr6	29925175
8597	Chr6	29925177
8598	Chr6	29925181

8599	Chr6	29925223
8600	Chr6	29925413
8601	Chr6	29925434
8602	Chr6	29925455
8603	Chr6	29925461
8604	Chr6	30044549
8605	Chr6	30044627
8606	Chr6	30044636
8607	Chr6	30044638
8608	Chr6	30044647
8609	Chr6	30044854
8610	Chr6	30045802
8611	Chr6	30045807
8612	Chr6	30045815
8613	Chr6	30045816
8614	Chr6	30045841
8615	Chr6	30045857
8616	Chr6	30046012
8617	Chr6	30046030
8618	Chr6	30046035
8619	Chr6	30046042
8620	Chr6	30046049
8621	Chr6	30046098
8622	Chr6	30046101
8623	Chr6	30054449
8624	Chr6	30054440
8625	Chr6	30054426
8626	Chr6	30054424
8627	Chr6	30054406
8628	Chr6	30054399
8629	Chr6	30054394
8630	Chr6	30054388
8631	Chr6	30054378
8632	Chr6	30054368
8633	Chr6	30054098
8634	Chr6	30054065
8635	Chr6	30054059
8636	Chr6	30071433
8637	Chr6	30071389
8638	Chr6	30508318
8639	Chr6	30508350
8640	Chr6	30508697
8641	Chr6	30508733
8642	Chr6	30508757
8643	Chr6	30528843
8644	Chr6	30528645
8645	Chr6	30551401

8646	Chr6	30551390
8647	Chr6	30551373
8648	Chr6	30566175
8649	Chr6	30566186
8650	Chr6	30566189
8651	Chr6	30566193
8652	Chr6	30566203
8653	Chr6	30566209
8654	Chr6	30566213
8655	Chr6	30566428
8656	Chr6	30566468
8657	Chr6	30566473
8658	Chr6	30566486
8659	Chr6	30566500
8660	Chr6	30566511
8661	Chr6	30566513
8662	Chr6	30593653
8663	Chr6	30593681
8664	Chr6	30593690
8665	Chr6	30594082
8666	Chr6	30594127
8667	Chr6	31168153
8668	Chr6	31209502
8669	Chr6	31209488
8670	Chr6	31958603
8671	Chr6	32630237
8672	Chr6	32629824
8673	Chr6	32629776
8674	Chr6	32629771
8675	Chr6	32797873
8676	Chr6	32797635
8677	Chr6	33936915
8678	Chr6	33937243
8679	Chr6	33937250
8680	Chr6	33937280
8681	Chr6	33937312
8682	Chr6	34426161
8683	Chr6	34446123
8684	Chr6	41891043
8685	Chr7	2784345
8686	Chr7	2813822
8687	Chr7	4499889
8688	Chr7	4499888
8689	Chr7	4527523
8690	Chr7	4527522
8691	Chr7	4527502
8692	Chr7	4527491

8693	Chr7	4527486
8694	Chr7	4527477
8695	Chr7	5340889
8696	Chr7	5384467
8697	Chr7	5861437
8698	Chr7	5911665
8699	Chr7	5911644
8700	Chr7	5911634
8701	Chr7	5911615
8702	Chr7	5911613
8703	Chr7	5911601
8704	Chr7	5911597
8705	Chr7	8672090
8706	Chr7	9612314
8707	Chr7	10136119
8708	Chr7	10136093
8709	Chr7	10136044
8710	Chr7	10136029
8711	Chr7	10592481
8712	Chr7	12246308
8713	Chr7	12246300
8714	Chr7	12246299
8715	Chr7	12246255
8716	Chr7	12246235
8717	Chr7	12245969
8718	Chr7	12245906
8719	Chr7	12245902
8720	Chr7	12245873
8721	Chr7	12245872
8722	Chr7	12567102
8723	Chr7	14403063
8724	Chr7	14998381
8725	Chr7	14998399
8726	Chr7	15299634
8727	Chr7	15644848
8728	Chr7	16135798
8729	Chr7	19497410
8730	Chr7	19497406
8731	Chr7	19497404
8732	Chr7	19497402
8733	Chr7	19497394
8734	Chr7	19497379
8735	Chr7	19497376
8736	Chr7	19497371
8737	Chr7	19497365
8738	Chr7	19497362
8739	Chr7	19497361

8740	Chr7	19497353
8741	Chr7	19565740
8742	Chr7	19565741
8743	Chr7	19845178
8744	Chr7	19882982
8745	Chr7	20458375
8746	Chr7	20467739
8747	Chr7	20471213
8748	Chr7	22266929
8749	Chr7	22665035
8750	Chr7	23204543
8751	Chr7	23937898
8752	Chr7	27476065
8753	Chr7	29149761
8754	Chr7	29149737
8755	Chr7	29341736
8756	Chr7	29341695
8757	Chr7	29341446
8758	Chr7	29966923
8759	Chr7	31451589
8760	Chr7	31900316
8761	Chr7	32497453
8762	Chr7	32725499
8763	Chr7	32725173
8764	Chr7	32725130
8765	Chr7	32737596
8766	Chr7	33787171
8767	Chr7	34786741
8768	Chr7	34786737
8769	Chr7	34786690
8770	Chr7	34786688
8771	Chr7	34786682
8772	Chr7	34886310
8773	Chr7	34886635
8774	Chr7	35421354
8775	Chr7	36430708
8776	Chr7	36430706
8777	Chr7	36430697
8778	Chr7	36430674
8779	Chr7	36430673
8780	Chr7	36430666
8781	Chr7	36430652
8782	Chr7	36430648
8783	Chr7	36430646
8784	Chr7	36430643
8785	Chr7	36430458
8786	Chr7	36430453

8787	Chr7	36430435
8788	Chr7	36430426
8789	Chr7	36430422
8790	Chr7	36430420
8791	Chr7	36430417
8792	Chr7	36430395
8793	Chr7	36430394
8794	Chr7	36430392
8795	Chr7	36430387
8796	Chr7	36430385
8797	Chr7	36430374
8798	Chr7	36430360
8799	Chr7	36925042
8800	Chr7	38541737
8801	Chr7	38994564
8802	Chr7	40459954
8803	Chr7	40459985
8804	Chr7	40834517
8805	Chr7	40834508
8806	Chr7	40834141
8807	Chr7	44265967
8808	Chr7	44265966
8809	Chr7	44265944
8810	Chr7	44265907
8811	Chr7	44265898
8812	Chr7	44265889
8813	Chr7	44265876
8814	Chr7	44265603
8815	Chr7	44265573
8816	Chr7	44265572
8817	Chr7	44265559
8818	Chr7	44265535
8819	Chr7	44419229
8820	Chr7	44720547
8821	Chr7	45074310
8822	Chr7	45098266
8823	Chr7	46214700
8824	Chr7	47490089
8825	Chr7	47490466
8826	Chr7	47490480
8827	Chr7	47491439
8828	Chr7	47491450
8829	Chr7	49657935
8830	Chr7	50630590
8831	Chr7	50630434
8832	Chr7	50630362
8833	Chr7	50630348

8834	Chr7	50630330
8835	Chr7	51805550
8836	Chr7	54173367
8837	Chr7	54173310
8838	Chr7	55625893
8839	Chr7	57571972
8840	Chr7	63631233
8841	Chr7	63631593
8842	Chr7	67188688
8843	Chr8	580550
8844	Chr8	621144
8845	Chr8	2881937
8846	Chr8	3213932
8847	Chr8	9359591
8848	Chr8	9359603
8849	Chr8	9359629
8850	Chr8	9359655
8851	Chr8	9419371
8852	Chr8	9419361
8853	Chr8	9419346
8854	Chr8	9419326
8855	Chr8	9419313
8856	Chr8	9431959
8857	Chr8	9966622
8858	Chr8	9970530
8859	Chr8	9970248
8860	Chr8	9983174
8861	Chr8	9983170
8862	Chr8	10010400
8863	Chr8	10010378
8864	Chr8	10010370
8865	Chr8	10010366
8866	Chr8	10013082
8867	Chr8	10894444
8868	Chr8	10894449
8869	Chr8	10894452
8870	Chr8	10894458
8871	Chr8	10894463
8872	Chr8	10894470
8873	Chr8	10894497
8874	Chr8	10894502
8875	Chr8	10894508
8876	Chr8	10894527
8877	Chr8	10894718
8878	Chr8	10894724
8879	Chr8	10894730
8880	Chr8	10894740

8881	Chr8	10894743
8882	Chr8	10894759
8883	Chr8	10894762
8884	Chr8	10894801
8885	Chr8	10894814
8886	Chr8	13867355
8887	Chr8	13867338
8888	Chr8	13867333
8889	Chr8	13867315
8890	Chr8	13867300
8891	Chr8	13867284
8892	Chr8	17709418
8893	Chr8	19046849
8894	Chr8	20722701
8895	Chr8	20722402
8896	Chr8	20722394
8897	Chr8	20722361
8898	Chr8	21424484
8899	Chr8	21424467
8900	Chr8	21424418
8901	Chr8	25429207
8902	Chr8	26317806
8903	Chr8	26317441
8904	Chr8	26317398
8905	Chr8	29300218
8906	Chr8	29300221
8907	Chr8	29300222
8908	Chr8	29300230
8909	Chr8	29300236
8910	Chr8	29300251
8911	Chr8	29300442
8912	Chr8	29300461
8913	Chr8	29300473
8914	Chr8	29300479
8915	Chr8	29300488
8916	Chr8	29300490
8917	Chr8	29300491
8918	Chr8	29300499
8919	Chr8	29300515
8920	Chr8	33893676
8921	Chr8	34707230
8922	Chr8	34707232
8923	Chr8	34707238
8924	Chr8	34707240
8925	Chr8	34707242
8926	Chr8	34707270
8927	Chr8	34707277

8928	Chr8	37295201
8929	Chr8	37295198
8930	Chr8	37295197
8931	Chr8	37295154
8932	Chr8	37295144
8933	Chr8	44750165
8934	Chr8	44750174
8935	Chr8	44750195
8936	Chr8	45933106
8937	Chr8	45933088
8938	Chr8	45933061
8939	Chr8	45933039
8940	Chr8	45932756
8941	Chr8	45932726
8942	Chr8	45932687
8943	Chr8	45932686
8944	Chr8	46460969
8945	Chr8	46460570
8946	Chr8	46460552
8947	Chr8	46678045
8948	Chr8	46678111
8949	Chr8	48365353
8950	Chr8	48365293
8951	Chr8	48365270
8952	Chr8	48365038
8953	Chr8	48364996
8954	Chr8	48364980
8955	Chr8	49212515
8956	Chr8	49212527
8957	Chr8	49212817
8958	Chr8	49212856
8959	Chr8	49861716
8960	Chr8	49861962
8961	Chr8	49862039
8962	Chr8	52555924
8963	Chr8	52555589
8964	Chr8	52555582
8965	Chr8	53593393
8966	Chr8	53593379
8967	Chr8	53839800
8968	Chr8	53839788
8969	Chr8	53839741
8970	Chr8	54966891
8971	Chr8	55097876
8972	Chr8	55097866
8973	Chr8	55097850
8974	Chr8	55097822

8975	Chr8	55097592
8976	Chr8	55097570
8977	Chr8	55312179
8978	Chr8	55312499
8979	Chr8	55343900
8980	Chr8	56756076
8981	Chr8	56756093
8982	Chr8	57609735
8983	Chr8	57638280
8984	Chr8	57661906
8985	Chr8	57662207
8986	Chr8	57665127
8987	Chr8	57665476
8988	Chr8	57665489
8989	Chr8	57971625
8990	Chr8	57971561
8991	Chr8	57971539
8992	Chr8	59891404
8993	Chr8	59891483
8994	Chr8	59891496
8995	Chr8	59891742
8996	Chr8	59891760
8997	Chr8	59891772
8998	Chr8	62923582
8999	Chr9	66961
9000	Chr9	544074
9001	Chr9	620312
9002	Chr9	973960
9003	Chr9	974010
9004	Chr9	975210
9005	Chr9	975396
9006	Chr9	1016794
9007	Chr9	1152462
9008	Chr9	1230690
9009	Chr9	1233996
9010	Chr9	1233806
9011	Chr9	1308886
9012	Chr9	1327807
9013	Chr9	1407040
9014	Chr9	1480289
9015	Chr9	1642770
9016	Chr9	6437707
9017	Chr9	6967001
9018	Chr9	6966964
9019	Chr9	6966931
9020	Chr9	6966598
9021	Chr9	6966585

9022	Chr9	6966580
9023	Chr9	6966555
9024	Chr9	8264865
9025	Chr9	9139441
9026	Chr9	13757064
9027	Chr9	13756977
9028	Chr9	13756698
9029	Chr9	13756654
9030	Chr9	13756648
9031	Chr9	13767381
9032	Chr9	13767737
9033	Chr9	13767771
9034	Chr9	13767785
9035	Chr9	14382215
9036	Chr9	14382216
9037	Chr9	14382231
9038	Chr9	14382242
9039	Chr9	14382289
9040	Chr9	14382539
9041	Chr9	14382548
9042	Chr9	14382549
9043	Chr9	14382556
9044	Chr9	14382563
9045	Chr9	14382579
9046	Chr9	14382590
9047	Chr9	14382595
9048	Chr9	14382619
9049	Chr9	14407366
9050	Chr9	14771775
9051	Chr9	15101457
9052	Chr9	22522850
9053	Chr9	22535826
9054	Chr9	23992099
9055	Chr9	23992078
9056	Chr9	24049919
9057	Chr9	24049912
9058	Chr9	24049888
9059	Chr9	24049587
9060	Chr9	24049581
9061	Chr9	24049554
9062	Chr9	24049523
9063	Chr9	24188521
9064	Chr9	24188520
9065	Chr9	26834556
9066	Chr9	26834577
9067	Chr9	26834591
9068	Chr9	26834595

9069	Chr9	26834612
9070	Chr9	26834639
9071	Chr9	27216368
9072	Chr9	28145263
9073	Chr9	28145272
9074	Chr9	28145281
9075	Chr9	28145289
9076	Chr9	28145294
9077	Chr9	28145323
9078	Chr9	29000205
9079	Chr9	31373691
9080	Chr9	33836381
9081	Chr9	33836348
9082	Chr9	34809715
9083	Chr9	34810097
9084	Chr9	37535565
9085	Chr9	38247907
9086	Chr9	40111316
9087	Chr9	40687817
9088	Chr9	40816189
9089	Chr9	42402064
9090	Chr9	42402011
9091	Chr9	44377638
9092	Chr9	44484737
9093	Chr9	47921307
9094	Chr9	47921299
9095	Chr9	47921255
9096	Chr9	47920895
9097	Chr9	47995008
9098	Chr9	48058079
9099	Chr9	48725325
9100	Chr9	52752968
9101	Chr9	52752965
9102	Chr9	52752964
9103	Chr9	52752954
9104	Chr9	52752949
9105	Chr9	52752948
9106	Chr9	52752936
9107	Chr9	52752919
9108	Chr9	52752916
9109	Chr9	52752897
9110	Chr9	52752714
9111	Chr9	52752713
9112	Chr9	52752705
9113	Chr9	52752702
9114	Chr9	52752694
9115	Chr9	52752692

9116	Chr9	52752669
9117	Chr9	52752660
9118	Chr9	52752655
9119	Chr9	52752647
9120	Chr9	52752637
9121	Chr9	55797696
9122	Chr9	55797700
9123	Chr9	55797707
9124	Chr9	55797715
9125	Chr9	55798057
9126	Chr9	55798114
9127	Chr9	56955071
9128	Chr9	58497563
9129	Chr9	61183466
9130	Chr9	65816097
9131	Chr9	65830795
9132	Chr9	71531690
9133	Chr9	71532263
9134	Chr10	564350
9135	Chr10	9334541
9136	Chr10	13991793
9137	Chr10	13991828
9138	Chr10	13992106
9139	Chr10	13992181
9140	Chr10	15556357
9141	Chr10	15831199
9142	Chr10	18463779
9143	Chr10	20991390
9144	Chr10	20991663
9145	Chr10	20991680
9146	Chr10	20991737
9147	Chr10	22491976
9148	Chr10	22491950
9149	Chr10	22491935
9150	Chr10	22491934
9151	Chr10	22491923
9152	Chr10	22491909
9153	Chr10	22491887
9154	Chr10	22862212
9155	Chr10	24887029
9156	Chr10	25895039
9157	Chr10	26313664
9158	Chr10	26313948
9159	Chr10	26314003
9160	Chr10	26341882
9161	Chr10	26438972
9162	Chr10	27531087

9163	Chr10	27642751
9164	Chr10	27642742
9165	Chr10	27642721
9166	Chr10	27642709
9167	Chr10	27642697
9168	Chr10	27642677
9169	Chr10	27642665
9170	Chr10	27642415
9171	Chr10	27642401
9172	Chr10	27642386
9173	Chr10	27642378
9174	Chr10	27642377
9175	Chr10	27642329
9176	Chr10	27645746
9177	Chr10	27645757
9178	Chr10	27645759
9179	Chr10	27645780
9180	Chr10	27645809
9181	Chr10	27645833
9182	Chr10	27645835
9183	Chr10	27646095
9184	Chr10	27646101
9185	Chr10	27646116
9186	Chr10	27646144
9187	Chr10	27646145
9188	Chr10	27646152
9189	Chr10	27646156
9190	Chr10	28199448
9191	Chr10	28199440
9192	Chr10	28199418
9193	Chr10	28199406
9194	Chr10	28199181
9195	Chr10	28199167
9196	Chr10	28199127
9197	Chr10	28582506
9198	Chr10	28582517
9199	Chr10	28582547
9200	Chr10	29386987
9201	Chr10	29387017
9202	Chr10	29387045
9203	Chr10	29387233
9204	Chr10	29387261
9205	Chr10	29387291
9206	Chr10	29386910
9207	Chr10	29386621
9208	Chr10	29386527
9209	Chr10	32828773

9210	Chr10	32828753
9211	Chr10	32828752
9212	Chr10	33818933
9213	Chr10	33818971
9214	Chr10	33819011
9215	Chr10	36652326
9216	Chr10	37319467
9217	Chr10	37319197
9218	Chr10	37319183
9219	Chr10	37319163
9220	Chr10	38632700
9221	Chr10	39005854
9222	Chr10	39005857
9223	Chr10	39383533
9224	Chr10	39383562
9225	Chr10	39383968
9226	Chr10	44189512
9227	Chr10	44189515
9228	Chr10	44189868
9229	Chr10	44189905
9230	Chr10	44434676
9231	Chr10	44708652
9232	Chr10	44709012
9233	Chr10	44709042
9234	Chr10	44709050
9235	Chr10	44709065
9236	Chr10	44709071
9237	Chr10	44709102
9238	Chr10	45031498
9239	Chr10	45031560
9240	Chr10	45031563
9241	Chr10	45031892
9242	Chr10	48881160
9243	Chr10	51063010
9244	Chr10	51062786
9245	Chr10	52183780
9246	Chr10	52183809
9247	Chr10	52183811
9248	Chr10	53982643
9249	Chr10	55319617
9250	Chr10	55319606
9251	Chr10	55319579
9252	Chr10	56631254
9253	Chr10	56631247
9254	Chr10	57045527
9255	Chr10	57045538
9256	Chr10	57659049

9257	Chr10	58431685
9258	Chr10	59231846
9259	Chr10	59909297
9260	Chr10	59909543
9261	Chr10	62460592
9262	Chr10	62494092
9263	Chr10	64208175
9264	Chr10	65515859
9265	Chr10	65515863
9266	Chr11	6412839
9267	Chr11	6746051
9268	Chr11	7576234
9269	Chr11	7744923
9270	Chr11	7744595
9271	Chr11	7857329
9272	Chr11	7927682
9273	Chr11	7927945
9274	Chr11	7927618
9275	Chr11	7948781
9276	Chr11	7987104
9277	Chr11	8017648
9278	Chr11	8017904
9279	Chr11	8017985
9280	Chr11	9487194
9281	Chr11	9487106
9282	Chr11	11008688
9283	Chr11	11108692
9284	Chr11	11108426
9285	Chr11	11108399
9286	Chr11	11108358
9287	Chr11	11432564
9288	Chr11	11588329
9289	Chr11	11588416
9290	Chr11	11588713
9291	Chr11	11588716
9292	Chr11	11588735
9293	Chr11	11588754
9294	Chr11	11588770
9295	Chr11	13177641
9296	Chr11	13177715
9297	Chr11	13178047
9298	Chr11	13177156
9299	Chr11	13225190
9300	Chr11	13225204
9301	Chr11	13249749
9302	Chr11	13249751
9303	Chr11	13267842

9304	Chr11	13465735
9305	Chr11	13476437
9306	Chr11	13512609
9307	Chr11	13544700
9308	Chr11	13544598
9309	Chr11	13544314
9310	Chr11	13618982
9311	Chr11	13709593
9312	Chr11	13980310
9313	Chr11	14031777
9314	Chr11	14213370
9315	Chr11	14249909
9316	Chr11	14249828
9317	Chr11	14290259
9318	Chr11	14290296
9319	Chr11	14291542
9320	Chr11	14341924
9321	Chr11	14444434
9322	Chr11	14483282
9323	Chr11	14513766
9324	Chr11	14560060
9325	Chr11	14609259
9326	Chr11	14633941
9327	Chr11	14661030
9328	Chr11	14685934
9329	Chr11	14729294
9330	Chr11	14758924
9331	Chr11	14837594
9332	Chr11	14837565
9333	Chr11	15070057
9334	Chr11	15095363
9335	Chr11	15095360
9336	Chr11	15100477
9337	Chr11	15100522
9338	Chr11	15765762
9339	Chr11	15794545
9340	Chr11	15845003
9341	Chr11	15845343
9342	Chr11	15896195
9343	Chr11	15896187
9344	Chr11	16013241
9345	Chr11	16021339
9346	Chr11	16021310
9347	Chr11	16021276
9348	Chr11	16050064
9349	Chr11	16054284
9350	Chr11	16176710

9351	Chr11	16177086
9352	Chr11	16255358
9353	Chr11	16294851
9354	Chr11	16300468
9355	Chr11	16391823
9356	Chr11	16391777
9357	Chr11	16467378
9358	Chr11	16469401
9359	Chr11	16469436
9360	Chr11	16468925
9361	Chr11	16470629
9362	Chr11	16480411
9363	Chr11	16503191
9364	Chr11	16502912
9365	Chr11	16742650
9366	Chr11	16777473
9367	Chr11	16875786
9368	Chr11	16876122
9369	Chr11	16892829
9370	Chr11	17006444
9371	Chr11	17132296
9372	Chr11	17194763
9373	Chr11	17432993
9374	Chr11	17449569
9375	Chr11	17449898
9376	Chr11	17473409
9377	Chr11	17482648
9378	Chr11	17482596
9379	Chr11	17482535
9380	Chr11	17492811
9381	Chr11	17535829
9382	Chr11	17608928
9383	Chr11	17608625
9384	Chr11	17612185
9385	Chr11	17667698
9386	Chr11	17704929
9387	Chr11	17742013
9388	Chr11	17769022
9389	Chr11	17795547
9390	Chr11	17806583
9391	Chr11	17813100
9392	Chr11	17846078
9393	Chr11	17865000
9394	Chr11	17912747
9395	Chr11	17921962
9396	Chr11	17921953
9397	Chr11	17921952

9398	Chr11	17921944
9399	Chr11	17952155
9400	Chr11	17957282
9401	Chr11	17957091
9402	Chr11	17962552
9403	Chr11	17962944
9404	Chr11	17962963
9405	Chr11	17963041
9406	Chr11	17962235
9407	Chr11	17964252
9408	Chr11	17989414
9409	Chr11	18011029
9410	Chr11	18011043
9411	Chr11	18018751
9412	Chr11	18033039
9413	Chr11	18032983
9414	Chr11	18049148
9415	Chr11	18049135
9416	Chr11	18055480
9417	Chr11	18705640
9418	Chr11	18705694
9419	Chr11	18715896
9420	Chr11	18716211
9421	Chr11	18723585
9422	Chr11	18723649
9423	Chr11	18723669
9424	Chr11	18723679
9425	Chr11	18724859
9426	Chr11	18724891
9427	Chr11	18724906
9428	Chr11	18727363
9429	Chr11	18727362
9430	Chr11	18770275
9431	Chr11	18854097
9432	Chr11	18853657
9433	Chr11	18853656
9434	Chr11	18864127
9435	Chr11	18864147
9436	Chr11	18866563
9437	Chr11	18866373
9438	Chr11	18866364
9439	Chr11	18866348
9440	Chr11	18866345
9441	Chr11	18965980
9442	Chr11	18965998
9443	Chr11	18977423
9444	Chr11	18977285

9445	Chr11	18977052
9446	Chr11	19053430
9447	Chr11	19086380
9448	Chr11	19178404
9449	Chr11	19243593
9450	Chr11	19243652
9451	Chr11	19243662
9452	Chr11	19244025
9453	Chr11	19278998
9454	Chr11	19306424
9455	Chr11	19327260
9456	Chr11	19326843
9457	Chr11	19380830
9458	Chr11	19647191
9459	Chr11	19660514
9460	Chr11	19660584
9461	Chr11	19703249
9462	Chr11	19710979
9463	Chr11	19710634
9464	Chr11	19735593
9465	Chr11	19735347
9466	Chr11	19735338
9467	Chr11	19735334
9468	Chr11	19762509
9469	Chr11	19777700
9470	Chr11	19919484
9471	Chr11	19919457
9472	Chr11	20000675
9473	Chr11	20025643
9474	Chr11	20040823
9475	Chr11	20065745
9476	Chr11	20065446
9477	Chr11	20065395
9478	Chr11	20117854
9479	Chr11	20117131
9480	Chr11	20219286
9481	Chr11	20398730
9482	Chr11	20446907
9483	Chr11	20469951
9484	Chr11	20469871
9485	Chr11	20469728
9486	Chr11	20469684
9487	Chr11	20469659
9488	Chr11	20500406
9489	Chr11	20500675
9490	Chr11	20523689
9491	Chr11	20601410

9492	Chr11	20611762
9493	Chr11	20612153
9494	Chr11	20630441
9495	Chr11	20715865
9496	Chr11	20763824
9497	Chr11	20773547
9498	Chr11	20819915
9499	Chr11	20873739
9500	Chr11	20873726
9501	Chr11	20873687
9502	Chr11	20873384
9503	Chr11	20924477
9504	Chr11	20924530
9505	Chr11	20924818
9506	Chr11	20924836
9507	Chr11	20938248
9508	Chr11	20938250
9509	Chr11	20970986
9510	Chr11	20971314
9511	Chr11	20970975
9512	Chr11	20970959
9513	Chr11	20970660
9514	Chr11	21072079
9515	Chr11	21115981
9516	Chr11	21116354
9517	Chr11	21122560
9518	Chr11	21153653
9519	Chr11	21175755
9520	Chr11	21248583
9521	Chr11	21268585
9522	Chr11	21268911
9523	Chr11	21311311
9524	Chr11	21414901
9525	Chr11	21460992
9526	Chr11	21460922
9527	Chr11	21513537
9528	Chr11	21521882
9529	Chr11	21581303
9530	Chr11	21687208
9531	Chr11	21774334
9532	Chr11	21803898
9533	Chr11	21910818
9534	Chr11	21987794
9535	Chr11	22086065
9536	Chr11	22086030
9537	Chr11	22089463
9538	Chr11	22092556

9539	Chr11	22235384
9540	Chr11	22249039
9541	Chr11	22249269
9542	Chr11	22308411
9543	Chr11	22310676
9544	Chr11	22310675
9545	Chr11	22516067
9546	Chr11	22543627
9547	Chr11	22608469
9548	Chr11	22608504
9549	Chr11	22624774
9550	Chr11	22644359
9551	Chr11	22670799
9552	Chr11	22670472
9553	Chr11	22675039
9554	Chr11	22674662
9555	Chr11	22697352
9556	Chr11	22697353
9557	Chr11	22698234
9558	Chr11	22697864
9559	Chr11	22823085
9560	Chr11	22823113
9561	Chr11	22823121
9562	Chr11	22823144
9563	Chr11	22823453
9564	Chr11	22832300
9565	Chr11	22832364
9566	Chr11	22889160
9567	Chr11	22889066
9568	Chr11	22889058
9569	Chr11	22890039
9570	Chr11	22890446
9571	Chr11	22904855
9572	Chr11	22905093
9573	Chr11	22905110
9574	Chr11	22904499
9575	Chr11	22904443
9576	Chr11	22940512
9577	Chr11	22980889
9578	Chr11	22981157
9579	Chr11	22989851
9580	Chr11	22996986
9581	Chr11	22997070
9582	Chr11	23013699
9583	Chr11	23013723
9584	Chr11	23014110
9585	Chr11	23032541

9586	Chr11	23104595
9587	Chr11	23104647
9588	Chr11	23136054
9589	Chr11	23146947
9590	Chr11	23146854
9591	Chr11	23149881
9592	Chr11	23371733
9593	Chr11	23371715
9594	Chr11	23381679
9595	Chr11	23593665
9596	Chr11	23593709
9597	Chr11	23603602
9598	Chr11	23603493
9599	Chr11	23603406
9600	Chr11	23608739
9601	Chr11	23608405
9602	Chr11	23644728
9603	Chr11	23644762
9604	Chr11	23649160
9605	Chr11	23649168
9606	Chr11	23649202
9607	Chr11	23649787
9608	Chr11	23649539
9609	Chr11	23649538
9610	Chr11	23653311
9611	Chr11	23730855
9612	Chr11	23737937
9613	Chr11	23737518
9614	Chr11	23737515
9615	Chr11	23773424
9616	Chr11	23773146
9617	Chr11	23773119
9618	Chr11	23789302
9619	Chr11	23789071
9620	Chr11	23801758
9621	Chr11	23801733
9622	Chr11	23859337
9623	Chr11	23862606
9624	Chr11	23942589
9625	Chr11	23943001
9626	Chr11	23944270
9627	Chr11	23944263
9628	Chr11	23944240
9629	Chr11	23976484
9630	Chr11	23976154
9631	Chr11	23976152
9632	Chr11	24032436

9633	Chr11	24054895
9634	Chr11	24144381
9635	Chr11	24156689
9636	Chr11	24156679
9637	Chr11	24167109
9638	Chr11	24166816
9639	Chr11	24237748
9640	Chr11	24280449
9641	Chr11	24280802
9642	Chr11	24295306
9643	Chr11	24295060
9644	Chr11	24304217
9645	Chr11	24321561
9646	Chr11	24349532
9647	Chr11	24513078
9648	Chr11	24673683
9649	Chr11	24675858
9650	Chr11	24676205
9651	Chr11	24744201
9652	Chr11	24748923
9653	Chr11	24749189
9654	Chr11	24749190
9655	Chr11	24807996
9656	Chr11	24874990
9657	Chr11	24879167
9658	Chr11	24900458
9659	Chr11	24907401
9660	Chr11	24911826
9661	Chr11	24932338
9662	Chr11	24932370
9663	Chr11	25222387
9664	Chr11	25222567
9665	Chr11	25222633
9666	Chr11	25222634
9667	Chr11	25222647
9668	Chr11	25228062
9669	Chr11	25267628
9670	Chr11	25293294
9671	Chr11	25293296
9672	Chr11	25293322
9673	Chr11	25299706
9674	Chr11	25299743
9675	Chr11	25428521
9676	Chr11	25438703
9677	Chr11	25438577
9678	Chr11	25452396
9679	Chr11	25452690

9680	Chr11	25470882
9681	Chr11	25470586
9682	Chr11	25503772
9683	Chr11	25522974
9684	Chr11	25522953
9685	Chr11	25633613
9686	Chr11	25654997
9687	Chr11	25659197
9688	Chr11	25659235
9689	Chr11	25658381
9690	Chr11	25699099
9691	Chr11	25699109
9692	Chr11	25698947
9693	Chr11	25736511
9694	Chr11	25736745
9695	Chr11	25737172
9696	Chr11	25754581
9697	Chr11	25771390
9698	Chr11	25885444
9699	Chr11	25884952
9700	Chr11	25924106
9701	Chr11	25963907
9702	Chr11	26106536
9703	Chr11	26122587
9704	Chr11	26122586
9705	Chr11	26269097
9706	Chr11	28157028
9707	Chr11	28156957
9708	Chr11	28177311
9709	Chr11	28178534
9710	Chr11	28281970
9711	Chr11	28281948
9712	Chr11	28294983
9713	Chr11	28302758
9714	Chr11	28371095
9715	Chr11	28387855
9716	Chr11	28387407
9717	Chr11	28393077
9718	Chr11	28434784
9719	Chr11	28436562
9720	Chr11	28436188
9721	Chr11	28436163
9722	Chr11	28452967
9723	Chr11	28452980
9724	Chr11	28466202
9725	Chr11	28466488
9726	Chr11	28466552

9727	Chr11	28466559
9728	Chr11	28536812
9729	Chr11	28565097
9730	Chr11	28565112
9731	Chr11	28565355
9732	Chr11	28701936
9733	Chr11	28701905
9734	Chr11	28701707
9735	Chr11	28746741
9736	Chr11	28777277
9737	Chr11	28777290
9738	Chr11	28777155
9739	Chr11	28799665
9740	Chr11	28892306
9741	Chr11	28911707
9742	Chr11	28911418
9743	Chr11	28937484
9744	Chr11	28957290
9745	Chr11	28964220
9746	Chr11	28998015
9747	Chr11	29016959
9748	Chr11	29017045
9749	Chr11	29076334
9750	Chr11	29128900
9751	Chr11	29128633
9752	Chr11	29128599
9753	Chr11	29172919
9754	Chr11	29172747
9755	Chr11	29172679
9756	Chr11	29187386
9757	Chr11	29193720
9758	Chr11	29194022
9759	Chr11	29223936
9760	Chr11	29224232
9761	Chr11	29224285
9762	Chr11	29245169
9763	Chr11	29245214
9764	Chr11	29245390
9765	Chr11	29245402
9766	Chr11	29252409
9767	Chr11	29252762
9768	Chr11	29273740
9769	Chr11	29273386
9770	Chr11	29273341
9771	Chr11	29273335
9772	Chr11	29277844
9773	Chr11	29277928

9774	Chr11	29278227
9775	Chr11	29278242
9776	Chr11	29278278
9777	Chr11	29308716
9778	Chr11	29308665
9779	Chr11	29363607
9780	Chr11	29363688
9781	Chr11	29364639
9782	Chr11	29381741
9783	Chr11	29387983
9784	Chr11	29413426
9785	Chr11	29446375
9786	Chr11	29470848
9787	Chr11	29470520
9788	Chr11	29510492
9789	Chr11	29510546
9790	Chr11	29510854
9791	Chr11	29523169
9792	Chr11	29624252
9793	Chr11	29624872
9794	Chr11	29633203
9795	Chr11	29633199
9796	Chr11	29657566
9797	Chr11	29657888
9798	Chr11	29670828
9799	Chr11	29670819
9800	Chr11	29687281
9801	Chr11	29699646
9802	Chr11	29795478
9803	Chr11	29798415
9804	Chr11	29798677
9805	Chr11	29798289
9806	Chr11	29956675
9807	Chr11	29956601
9808	Chr11	29957731
9809	Chr11	29957748
9810	Chr11	29957801
9811	Chr11	29965475
9812	Chr11	29965462
9813	Chr11	29965236
9814	Chr11	29965206
9815	Chr11	30005699
9816	Chr11	30005356
9817	Chr11	30005307
9818	Chr11	30141837
9819	Chr11	30141772
9820	Chr11	30141416

9821	Chr11	30152312
9822	Chr11	30156659
9823	Chr11	30184935
9824	Chr11	30184545
9825	Chr11	30198261
9826	Chr11	30211962
9827	Chr11	30221129
9828	Chr11	30221439
9829	Chr11	30233858
9830	Chr11	30233833
9831	Chr11	30233797
9832	Chr11	30250331
9833	Chr11	30250076
9834	Chr11	30280940
9835	Chr11	30281196
9836	Chr11	30288236
9837	Chr11	30288302
9838	Chr11	30288443
9839	Chr11	30292721
9840	Chr11	30303257
9841	Chr11	30303253
9842	Chr11	30303217
9843	Chr11	30303215
9844	Chr11	30327542
9845	Chr11	30340894
9846	Chr11	30363563
9847	Chr11	30363835
9848	Chr11	30363900
9849	Chr11	30387551
9850	Chr11	30418516
9851	Chr11	30424580
9852	Chr11	30424441
9853	Chr11	30452941
9854	Chr11	30491606
9855	Chr11	30491362
9856	Chr11	30723585
9857	Chr11	30755373
9858	Chr11	30766172
9859	Chr11	30766539
9860	Chr11	30792063
9861	Chr11	30791830
9862	Chr11	30806690
9863	Chr11	30806692
9864	Chr11	30806712
9865	Chr11	30806944
9866	Chr11	30806968
9867	Chr11	30820187

9868	Chr11	30820556
9869	Chr11	30847060
9870	Chr11	30847119
9871	Chr11	30846933
9872	Chr11	30958174
9873	Chr11	31164778
9874	Chr11	31190119
9875	Chr11	31190585
9876	Chr11	31282745
9877	Chr11	31282342
9878	Chr11	31343067
9879	Chr11	31342792
9880	Chr11	31342783
9881	Chr11	31342768
9882	Chr11	31368275
9883	Chr11	31402633
9884	Chr11	31404664
9885	Chr11	31479789
9886	Chr11	31528406
9887	Chr11	31770914
9888	Chr11	31942116
9889	Chr11	32015254
9890	Chr11	32016547
9891	Chr11	32016848
9892	Chr11	32147273
9893	Chr11	32208371
9894	Chr11	32355345
9895	Chr11	32355353
9896	Chr11	32489072
9897	Chr11	32500578
9898	Chr11	32615940
9899	Chr11	32615914
9900	Chr11	32681868
9901	Chr11	32718265
9902	Chr11	32742278
9903	Chr11	32810885
9904	Chr11	32826636
9905	Chr11	32826929
9906	Chr11	32892917
9907	Chr11	32892930
9908	Chr11	32892934
9909	Chr11	32892948
9910	Chr11	32893309
9911	Chr11	32918664
9912	Chr11	32922941
9913	Chr11	33007951
9914	Chr11	33104402

9915	Chr11	33267812
9916	Chr11	33278595
9917	Chr11	33357164
9918	Chr11	33401234
9919	Chr11	33426399
9920	Chr11	33425924
9921	Chr11	33597375
9922	Chr11	33597353
9923	Chr11	33598591
9924	Chr11	33674698
9925	Chr11	33783417
9926	Chr11	33845044
9927	Chr11	33846841
9928	Chr11	33849579
9929	Chr11	33849624
9930	Chr11	33849922
9931	Chr11	33849932
9932	Chr11	33849434
9933	Chr11	33849183
9934	Chr11	33875450
9935	Chr11	33880472
9936	Chr11	33880523
9937	Chr11	33885411
9938	Chr11	33903818
9939	Chr11	33903786
9940	Chr11	33911063
9941	Chr11	33911232
9942	Chr11	33970346
9943	Chr11	33970068
9944	Chr11	33981862
9945	Chr11	34096405
9946	Chr11	34217678
9947	Chr11	34217443
9948	Chr11	34218253
9949	Chr11	34218257
9950	Chr11	34219561
9951	Chr11	34231018
9952	Chr11	34325366
9953	Chr11	34325156
9954	Chr11	34325883
9955	Chr11	34331819
9956	Chr11	34349227
9957	Chr11	34460800
9958	Chr11	34461099
9959	Chr11	34480968
9960	Chr11	34591213
9961	Chr11	34603197

9962	Chr11	34605505
9963	Chr11	34684413
9964	Chr11	34701532
9965	Chr11	34701229
9966	Chr11	34712146
9967	Chr11	34748495
9968	Chr11	34754028
9969	Chr11	34754057
9970	Chr11	34754229
9971	Chr11	34961081
9972	Chr11	34984547
9973	Chr11	34984996
9974	Chr11	35004903
9975	Chr11	35004904
9976	Chr11	35014773
9977	Chr11	35028305
9978	Chr11	35030392
9979	Chr11	35029902
9980	Chr11	35074779
9981	Chr11	35090366
9982	Chr11	35146885
9983	Chr11	35191457
9984	Chr11	35191455
9985	Chr11	35191418
9986	Chr11	35220166
9987	Chr11	35238163
9988	Chr11	35242067
9989	Chr11	35241876
9990	Chr11	35241560
9991	Chr11	35247247
9992	Chr11	35298947
9993	Chr11	35299000
9994	Chr11	35452996
9995	Chr11	35494305
9996	Chr11	35494116
9997	Chr11	35527355
9998	Chr11	35535267
9999	Chr11	35535033
10000	Chr11	35535026
10001	Chr11	35547745
10002	Chr11	35547768
10003	Chr11	35548095
10004	Chr11	35587566
10005	Chr11	35613368
10006	Chr11	35613342
10007	Chr11	35613007
10008	Chr11	35668298

10009	Chr11	35693512
10010	Chr11	35693245
10011	Chr11	35701240
10012	Chr11	35709356
10013	Chr11	35772540
10014	Chr11	35772756
10015	Chr11	35793537
10016	Chr11	35902759
10017	Chr11	35956706
10018	Chr11	36041428
10019	Chr11	36093126
10020	Chr11	36097699
10021	Chr11	36114011
10022	Chr11	36303058
10023	Chr11	36307425
10024	Chr11	36307148
10025	Chr11	36558247
10026	Chr11	36560031
10027	Chr11	36578694
10028	Chr11	36683609
10029	Chr11	36705518
10030	Chr11	36714281
10031	Chr11	36753869
10032	Chr11	36753447
10033	Chr11	36793372
10034	Chr11	36793387
10035	Chr11	36793393
10036	Chr11	36793407
10037	Chr11	36793408
10038	Chr11	36793437
10039	Chr11	36793438
10040	Chr11	36793455
10041	Chr11	36793466
10042	Chr11	36851319
10043	Chr11	36925855
10044	Chr11	36959226
10045	Chr11	36967351
10046	Chr11	36967408
10047	Chr11	37105514
10048	Chr11	37105418
10049	Chr11	37105116
10050	Chr11	37287243
10051	Chr11	37287013
10052	Chr11	37355472
10053	Chr11	37355515
10054	Chr11	37355819
10055	Chr11	37365066

10056	Chr11	37370842
10057	Chr11	37370861
10058	Chr11	37371133
10059	Chr11	37371174
10060	Chr11	37446988
10061	Chr11	37446806
10062	Chr11	37467242
10063	Chr11	37485797
10064	Chr11	37522513
10065	Chr11	37551499
10066	Chr11	37568481
10067	Chr11	37604671
10068	Chr11	37607269
10069	Chr11	37607266
10070	Chr11	37617528
10071	Chr11	37635199
10072	Chr11	37642036
10073	Chr11	37677797
10074	Chr11	37684567
10075	Chr11	37684475
10076	Chr11	37837640
10077	Chr11	37844632
10078	Chr11	37844398
10079	Chr11	37933908
10080	Chr11	38101184
10081	Chr11	38101183
10082	Chr11	38101117
10083	Chr11	38208291
10084	Chr11	38240601
10085	Chr11	38452482
10086	Chr11	38655202
10087	Chr11	38655192
10088	Chr11	38689665
10089	Chr11	38689972
10090	Chr11	38711208
10091	Chr11	38711207
10092	Chr11	38789751
10093	Chr11	38789785
10094	Chr11	38789941
10095	Chr11	38825298
10096	Chr11	38852147
10097	Chr11	38862935
10098	Chr11	38862921
10099	Chr11	38862917
10100	Chr11	38962218
10101	Chr11	38982476
10102	Chr11	38982484

10103	Chr11	39008742
10104	Chr11	39008724
10105	Chr11	39008517
10106	Chr11	39053998
10107	Chr11	39065383
10108	Chr11	39065723
10109	Chr11	39065742
10110	Chr11	39165791
10111	Chr11	39165761
10112	Chr11	39165726
10113	Chr11	39165462
10114	Chr11	39165456
10115	Chr11	39165453
10116	Chr11	39165411
10117	Chr11	39287947
10118	Chr11	39287923
10119	Chr11	39287911
10120	Chr11	39380543
10121	Chr11	39387612
10122	Chr11	39429167
10123	Chr11	39488287
10124	Chr11	39562267
10125	Chr11	39615437
10126	Chr11	39626381
10127	Chr11	39677587
10128	Chr11	39678701
10129	Chr11	39692941
10130	Chr11	39769155
10131	Chr11	39768840
10132	Chr11	39777153
10133	Chr11	39776754
10134	Chr11	39811384
10135	Chr11	39814353
10136	Chr11	39922460
10137	Chr11	39922466
10138	Chr11	39932725
10139	Chr11	39932794
10140	Chr11	39977638
10141	Chr11	39977974
10142	Chr11	40138356
10143	Chr11	40171860
10144	Chr11	40219348
10145	Chr11	40302298
10146	Chr11	40302251
10147	Chr11	40302250
10148	Chr11	40312792
10149	Chr11	40409596

10150	Chr11	40409602
10151	Chr11	40527280
10152	Chr11	40527634
10153	Chr11	40607905
10154	Chr11	40607891
10155	Chr11	40709369
10156	Chr11	40709370
10157	Chr11	40735134
10158	Chr11	40748069
10159	Chr11	40748424
10160	Chr11	40748443
10161	Chr11	40749087
10162	Chr11	40835429
10163	Chr11	40850057
10164	Chr11	40887507
10165	Chr11	40927479
10166	Chr11	40927480
10167	Chr11	40936659
10168	Chr11	40937044
10169	Chr11	40937097
10170	Chr11	40957730
10171	Chr11	40969623
10172	Chr11	41084308
10173	Chr11	41097077
10174	Chr11	41105337
10175	Chr11	41105306
10176	Chr11	41116947
10177	Chr11	41135075
10178	Chr11	41143650
10179	Chr11	41311023
10180	Chr11	41315036
10181	Chr11	41315019
10182	Chr11	41319831
10183	Chr11	41347838
10184	Chr11	41347854
10185	Chr11	41347889
10186	Chr11	41348073
10187	Chr11	41360296
10188	Chr11	41375881
10189	Chr11	41407788
10190	Chr11	41450959
10191	Chr11	41482209
10192	Chr11	41497814
10193	Chr11	41500853
10194	Chr11	41500803
10195	Chr11	41535262
10196	Chr11	41535279

10197	Chr11	41552670
10198	Chr11	41636646
10199	Chr11	41636647
10200	Chr11	41636940
10201	Chr11	41698627
10202	Chr11	41815088
10203	Chr11	41846536
10204	Chr11	41863007
10205	Chr11	41863008
10206	Chr11	41867915
10207	Chr11	41869306
10208	Chr11	41905911
10209	Chr11	41915785
10210	Chr11	41929806
10211	Chr11	41934307
10212	Chr11	41970753
10213	Chr11	41971104
10214	Chr11	41988780
10215	Chr11	41988779
10216	Chr11	42211077
10217	Chr11	42217740
10218	Chr11	42224476
10219	Chr11	42224477
10220	Chr11	42224485
10221	Chr11	42247939
10222	Chr11	42267435
10223	Chr11	42267667
10224	Chr11	42266876
10225	Chr11	42413123
10226	Chr11	42440638
10227	Chr11	42440397
10228	Chr11	42498631
10229	Chr11	42498630
10230	Chr11	42498607
10231	Chr11	42500513
10232	Chr11	42500476
10233	Chr11	42601502
10234	Chr11	42636891
10235	Chr11	42646532
10236	Chr11	42751843
10237	Chr11	42751864
10238	Chr11	42752009
10239	Chr11	42763617
10240	Chr11	42768911
10241	Chr11	42772490
10242	Chr11	42789181
10243	Chr11	42789198

10244	Chr11	42789535
10245	Chr11	42885801
10246	Chr11	42885645
10247	Chr11	42888046
10248	Chr11	42888095
10249	Chr11	42888135
10250	Chr11	42888385
10251	Chr11	42887636
10252	Chr11	42954543
10253	Chr11	42989199
10254	Chr11	43005304
10255	Chr11	43038274
10256	Chr11	43111308
10257	Chr11	43112746
10258	Chr11	43247425
10259	Chr11	43310069
10260	Chr11	43309995
10261	Chr11	43336550
10262	Chr11	43336581
10263	Chr11	43336736
10264	Chr11	43342935
10265	Chr11	43352970
10266	Chr11	43352925
10267	Chr11	43372474
10268	Chr11	43372129
10269	Chr11	43372113
10270	Chr11	43404625
10271	Chr11	43404603
10272	Chr11	43404588
10273	Chr11	43423906
10274	Chr11	43451258
10275	Chr11	43452483
10276	Chr11	43478481
10277	Chr11	43513687
10278	Chr11	43513412
10279	Chr11	43639826
10280	Chr11	43639823
10281	Chr11	43639810
10282	Chr11	43854694
10283	Chr11	43904951
10284	Chr11	43959496
10285	Chr11	43999241
10286	Chr11	44050683
10287	Chr11	44061261
10288	Chr11	44106317
10289	Chr11	44110633
10290	Chr11	44110391

10291	Chr11	44127921
10292	Chr11	44127530
10293	Chr11	44137112
10294	Chr11	44144865
10295	Chr11	44177897
10296	Chr11	44306538
10297	Chr11	44342397
10298	Chr11	44371220
10299	Chr11	44383064
10300	Chr11	44382665
10301	Chr11	44382338
10302	Chr11	44391635
10303	Chr11	44407799
10304	Chr11	44444934
10305	Chr11	44455334
10306	Chr11	44455655
10307	Chr11	44455676
10308	Chr11	44484505
10309	Chr11	44552314
10310	Chr11	44552356
10311	Chr11	44559077
10312	Chr11	44559413
10313	Chr11	44611068
10314	Chr11	44712376
10315	Chr11	44753317
10316	Chr11	44792473
10317	Chr11	44792228
10318	Chr11	44894031
10319	Chr11	44905408
10320	Chr11	44929003
10321	Chr11	44952834
10322	Chr11	44983866
10323	Chr11	45006600
10324	Chr11	45103712
10325	Chr11	45104010
10326	Chr11	45103644
10327	Chr11	45103622
10328	Chr11	45116861
10329	Chr11	45116907
10330	Chr11	45144327
10331	Chr11	45160065
10332	Chr11	45160265
10333	Chr11	45160295
10334	Chr11	45184796
10335	Chr11	45234467
10336	Chr11	45242350
10337	Chr11	45242344

10338	Chr11	45264135
10339	Chr11	45263687
10340	Chr11	45263681
10341	Chr11	45278194
10342	Chr11	45279857
10343	Chr11	45279858
10344	Chr11	45279901
10345	Chr11	45282138
10346	Chr11	45282125
10347	Chr11	45282108
10348	Chr11	45292456
10349	Chr11	45293862
10350	Chr11	45293431
10351	Chr11	45294095
10352	Chr11	45294348
10353	Chr11	45294625
10354	Chr11	45307035
10355	Chr11	45318546
10356	Chr11	45318161
10357	Chr11	45327993
10358	Chr11	45327945
10359	Chr11	45328735
10360	Chr11	45328695
10361	Chr11	45328678
10362	Chr11	45333718
10363	Chr11	45334987
10364	Chr11	45334990
10365	Chr11	45335384
10366	Chr11	45335408
10367	Chr11	45335428
10368	Chr11	45337266
10369	Chr11	45337579
10370	Chr11	45337598
10371	Chr11	45343765
10372	Chr11	45343811
10373	Chr11	45344072
10374	Chr11	45345611
10375	Chr11	45375762
10376	Chr11	45375954
10377	Chr11	45398518
10378	Chr11	45399484
10379	Chr11	45404673
10380	Chr11	45409989
10381	Chr11	45409931
10382	Chr11	45414193
10383	Chr11	45419851
10384	Chr11	45419893

10385	Chr11	45423446
10386	Chr11	45423107
10387	Chr11	45423055
10388	Chr11	45425599
10389	Chr11	45425169
10390	Chr11	45424929
10391	Chr11	45439072
10392	Chr11	45439293
10393	Chr11	45439716
10394	Chr11	45439752
10395	Chr11	45440025
10396	Chr11	45443644
10397	Chr11	45443474
10398	Chr11	45444645
10399	Chr11	45444324
10400	Chr11	45745289
10401	Chr11	45746303
10402	Chr11	45746430
10403	Chr11	45805294
10404	Chr11	45806114
10405	Chr11	45806092
10406	Chr11	45805781
10407	Chr11	45808269
10408	Chr11	45821981
10409	Chr11	45846806
10410	Chr11	45847137
10411	Chr11	45847156
10412	Chr11	45849117
10413	Chr11	45849085
10414	Chr11	45849051
10415	Chr11	45848751
10416	Chr11	45848737
10417	Chr11	45848728
10418	Chr11	45848700
10419	Chr11	45848688
10420	Chr11	45855275
10421	Chr11	45865339
10422	Chr11	45865325
10423	Chr11	45865323
10424	Chr11	45865310
10425	Chr11	45865299
10426	Chr11	45865294
10427	Chr11	45865059
10428	Chr11	45865056
10429	Chr11	45865047
10430	Chr11	45872337
10431	Chr11	45872221

10432	Chr11	45877230
10433	Chr11	45877231
10434	Chr11	45877236
10435	Chr11	45877914
10436	Chr11	45877643
10437	Chr11	45877581
10438	Chr11	45877561
10439	Chr11	45887845
10440	Chr11	45887848
10441	Chr11	45895334
10442	Chr11	45895059
10443	Chr11	45896085
10444	Chr11	45896993
10445	Chr11	45896986
10446	Chr11	45896977
10447	Chr11	45900113
10448	Chr11	45908764
10449	Chr11	45908443
10450	Chr11	45910146
10451	Chr11	45909621
10452	Chr11	45911270
10453	Chr11	45922616
10454	Chr11	45922578
10455	Chr11	45945287
10456	Chr11	45945302
10457	Chr11	46036833
10458	Chr11	46246884
10459	Chr11	46246635
10460	Chr11	46246571
10461	Chr11	46257002
10462	Chr11	46257042
10463	Chr11	46257052
10464	Chr11	46257057
10465	Chr11	46258269
10466	Chr11	46258537
10467	Chr11	46258591
10468	Chr11	46258626
10469	Chr11	46287710
10470	Chr11	46303926
10471	Chr11	46482674
10472	Chr11	46609256
10473	Chr11	46654541
10474	Chr11	46654782
10475	Chr11	46689593
10476	Chr11	46878967
10477	Chr11	46928633
10478	Chr11	46928600

10479	Chr11	46928588
10480	Chr11	46928259
10481	Chr11	46928255
10482	Chr11	46929908
10483	Chr11	46929898
10484	Chr11	46929573
10485	Chr11	46929549
10486	Chr11	46978245
10487	Chr11	46978279
10488	Chr11	47128623
10489	Chr11	47128953
10490	Chr11	47129003
10491	Chr11	47188657
10492	Chr11	47193715
10493	Chr11	47193714
10494	Chr11	47193306
10495	Chr11	47312392
10496	Chr11	47312270
10497	Chr11	47445959
10498	Chr11	47445945
10499	Chr11	48377932
10500	Chr11	48388916
10501	Chr11	48462994
10502	Chr11	48549318
10503	Chr11	48766298
10504	Chr11	48810933
10505	Chr11	48824093
10506	Chr11	48833785
10507	Chr11	48833820
10508	Chr11	48834106
10509	Chr11	48865214
10510	Chr11	48865218
10511	Chr11	48865523
10512	Chr11	48880660
10513	Chr11	48880725
10514	Chr11	48888101
10515	Chr11	48887956
10516	Chr11	48980707
10517	Chr11	48980372
10518	Chr11	49028776
10519	Chr11	49054776
10520	Chr11	49055103
10521	Chr11	49115801
10522	Chr11	49166653
10523	Chr11	49282842
10524	Chr11	49348926
10525	Chr11	49478597

10526	Chr11	52109579
10527	Chr11	52220295
10528	Chr11	52409971
10529	Chr11	52425226
10530	Chr11	52477095
10531	Chr11	53601623
10532	Chr11	54813111
10533	Chr11	55299874
10534	Chr11	55363448
10535	Chr11	55365140
10536	Chr12	1195454
10537	Chr12	1195469
10538	Chr12	1195492
10539	Chr12	1195493
10540	Chr12	1195499
10541	Chr12	1195501
10542	Chr12	1195528
10543	Chr12	1195530
10544	Chr12	1195535
10545	Chr12	1195543
10546	Chr12	2951589
10547	Chr12	2951593
10548	Chr12	2951615
10549	Chr12	10025653
10550	Chr12	10025432
10551	Chr12	10219322
10552	Chr12	10219286
10553	Chr12	10219239
10554	Chr12	10219230
10555	Chr12	10218939
10556	Chr12	10218875
10557	Chr12	10218871
10558	Chr12	15483189
10559	Chr12	15483372
10560	Chr12	15483378
10561	Chr12	15483415
10562	Chr12	15483453
10563	Chr12	17485996
10564	Chr12	17604517
10565	Chr12	17608817
10566	Chr12	18964706
10567	Chr12	18964738
10568	Chr12	22496031
10569	Chr12	22522707
10570	Chr12	22525204
10571	Chr12	22639762
10572	Chr12	22639681

10573	Chr12	22639658
10574	Chr12	22661805
10575	Chr12	22661837
10576	Chr12	22687208
10577	Chr12	22687163
10578	Chr12	22687125
10579	Chr12	22748106
10580	Chr12	22748019
10581	Chr12	22747700
10582	Chr12	22748801
10583	Chr12	22748904
10584	Chr12	22749111
10585	Chr12	22749131
10586	Chr12	22828163
10587	Chr12	22828162
10588	Chr12	22972268
10589	Chr12	25841422
10590	Chr12	26961964
10591	Chr12	32811728
10592	Chr12	32811707
10593	Chr12	34411394
10594	Chr12	34411696
10595	Chr12	34411697
10596	Chr12	34411724
10597	Chr12	36259666
10598	Chr12	36329248
10599	Chr12	36346178
10600	Chr12	37302330
10601	Chr12	37426737
10602	Chr12	38064316
10603	Chr12	38064308
10604	Chr12	38095571
10605	Chr12	38098657
10606	Chr12	38274165
10607	Chr12	38273875
10608	Chr12	38279431
10609	Chr12	38287172
10610	Chr12	38286728
10611	Chr12	38286413
10612	Chr12	42709592
10613	Chr12	42709619
10614	Chr12	42709876
10615	Chr12	42709929
10616	Chr12	42709543
10617	Chr12	42709542
10618	Chr12	42709530
10619	Chr12	42709521

10620	Chr12	42709500
10621	Chr12	42709499
10622	Chr12	42709474
10623	Chr12	42709464
10624	Chr12	42709304
10625	Chr12	42709275
10626	Chr12	50487425
10627	Chr12	50487782
10628	Chr12	50487807
10629	Chr12	51047261
10630	Chr12	54119248
10631	Chr12	54119218
10632	Chr12	54119216
10633	Chr12	54119167
10634	Chr12	54118879
10635	Chr12	54118850
10636	Chr12	59496900
10637	Chr12	59496543
10638	Chr12	59496314
10639	Chr12	59496229
10640	Chr12	59662784
10641	Chr12	60229583
10642	Chr12	60292256
10643	Chr12	60914184
10644	Chr12	60914185
10645	Chr12	62248231
10646	Chr12	62369435
10647	Chr12	63178933
10648	Chr12	65339391
