# Supplementary material for: Tomato genomic prediction for good performance under high-temperature and identification of loci involved in thermotolerance response
Source: Hortic Res. 2021 Oct 1;8:212. doi: 10.1038/s41438-021-00647-3 (PMC8484564; doi:10.1038/s41438-021-00647-3)
Supplement: Supplementary file 2 — Table S1 [file 41438_2021_647_MOESM2_ESM.pdf]

Table S1. Mean values of phenotypic traits analysed under heat stress conditions during the

Gen.	YP	TFN	SSC	CR	FS	IN
F4S208	2	50	4,2	5	41	3
F4S98	10	215	4,3	1	54,25	3
F4S68	9,8	152	4,3	1	35	1
F4S204	4,5	90	4,6	3	16	1
F4S76	13,7	333	5	1	53	1
F4S28	10,5	230	4	1	78	4
F4S121	7,3	180	4,6	1	35	2
F4S187	4	125	4,6	1	19,25	4
F4S218	6,6	110	4,8	1	24	3
F4S61	5	91	4	1	27	1
F4S60	13	335	4,2	1	40	1
F4S183	5	60	5,8	5	20	1
F4S224	8	145	4,4	1	50	4
F4S235	4,5	50	4,2	3	20	1
F4S156	3,4	110	4,4	3	20	5
F4S185	6,5	77	4	1	24	2
F4S244	10	205	3,5	1	29	3
F4S106	9,6	167	4	3	31	1
F4S39	11,7	211	4,5	1	53,7	2
F4S237	5	55	3,2	3	22	2
F4S249	12,5	130	3,7	1	65	4
F4S220	4	58	4,7	3	15	2
F4S180	6,7	115	5,4	5	27	5
F4S127	9,3	173	5,5	1	40	2
F4S115	4,8	119	4,5	5	60	1
F4S205	7,2	80	4	5	44	1
F4S153	2,3	45	4	3	27	4
F4S55	10,1	199	3	1	32,5	4
F4S67	8,2	145	3,9	3	30	2
F4S36	9,7	176	4,2	1	47	1
F4S201	4	105	4,6	4	24	2
F4S231	4,5	60	4	5	42	1
F4S150	4,8	80	4,9	4	8	2
F4S31	10,8	218	4,4	1	47	1
F4S62	8	139	4,7	1	59	2
F4S33	10,8	224	3.3	3	46,6	4
F4S30	11,6	306	3,7	1	43,5	4
F4S53	16	350	3,3	1	53,7	3
F4S196	12,5	249	4	2	50	5
F4S128	6,1	169	4	3	33	2
F4S4	6,3	139	4,3	1	30	1
F4S112	8,5	145	4,8	3	40	3
F4S122	4	82	4,6	4	28	1
F4S194	9,8	177	4	1	57	5

F4S34	12	310	4,7	5	68	3
F4S97	6,5	127	5	3	29	1
F4S91	11,5	270	3,8	1	63	3
F4S1	7,5	129	5	5	21,75	1
F4S82	10	147	4,8	3	42	1
F4S160	5	80	5	3	10	4
F4S22	9	115	4,3	5	40	1
F4S225	3,5	70	4.1	5	34	2
F4S75	13,5	330	3,6	1	73	4
F4S100	5,6	80	6,8	1	43	1
F4S99	12	208	4,1	1	65	3
S133	3	125	5,5	5	29	1
F4S13	16	295	4,2	1	45	5
F4S20	7,2	150	4	5	45,25	3
F4S113	6	100	5,5	3	48	1
F4S118	9,8	191	4,1	5	44	2
F4S8	13,7	248	4,1	1	47	5
F4S117	6,5	229	5	5	66	1
F4S2	9,7	201	4,5	5	38,25	3
F4S41	16	354	4,5	1	74,26	2
F4S90	6	132	4,7	5	35,5	1
F4S69	10	209	4,5	1	38	1
F4S221	10,3	195	5,4	5	35	2
F4S92	15	239	4,2	1	41	3
F4S254	6	100	3,8	1	22	1
F4S47	1,65	62	4,5	5	44	1
F4S147	7	106	4,5	3	33	1
F4S84	9.5	178	4,8	2	47	3
F4S10	8,9	213	5,2	1	51,25	1
F4S179	7	100	3,8	1	25	1
F4S5	10	221	3,7	1	43,5	3
F4S63	7,7	160	3,6	1	43	2
F4S64	8,7	200	4,7	1	20	2
F4S159	10	200	3,9	3	9	2
F4S66	5,5	70	5,2	5	35	2
F4S126	0,8	32	5,4	3	27	2
F4S188	6,2	105	4,1	1	19	2
F4S48	13	242	4,5	2	54	3
F4S114	6	115	5	3	28	1
F4S77	11,8	222	4	2	47	3
F4S51	8,7	173	3,6	5	23	2
F4S174	4,3	92	4,5	5	50	3
F4S21	8	140	4,5	5	57,2	3
F4S139	2	50	4,6	5	31	1
F4S239	4	80	5	5	20	3
F4S9	4,5	184	4,5	5	41,25	3
F4S105	11,5	246	4,7	1	34	3
F4S104	5,8	88	4,3	3	8	1

F4S27	13,8	238	4	1	61,5	5
F4S130	7,5	90	4.1	1	51	3
F4S7	4	78	4,4	4	20	2
F4S83	5,2	148	4,5	3	47	1
F4S252	3,5	70	5,1	3	22	1
F4S255	13	235	4,7	2	45	4
F4S205_2	7,3	80	4	3	44	1
F4S75_2	13,8	297	3,6	1	72	4

s during the summer 2017.

LC	FRL
3	1
4	2
2	3
4	1
2	1
4	1
4	1
3	1
2	1
4	1
5	2
2	1
4	1
2	1
5	1
5	1
2	2
4	1
3	1
3	3
2	4
5	2
2	1
5	1
2	1
2	2
2	4
5	2
3	1
4	1
4	1
3	1
3	2
4	1
4	1
3	1
5	1
4	4
3	3
2	1
4	1
3	3
1	1
2	2

2	2
3	1
4	4
2	3
4	1
3	1
2	1
4	1
3	3
4	1
4	5
2	1
4	5
4	4
4	1
1	2
4	3
2	2
1	4
5	1
2	1
5	1
5	2
5	5
3	3
4	1
2	3
3	3
4	1
3	3
4	1
4	2
5	1
2	1
1	1
5	1
4	1
2	1
4	1
5	1
3	5
1	1
1	2
4	1
1	1
1	3
5	1
5	1

3	1
3	1
5	4
3	1
4	1
2	1
2	3
3	3
