# Supplementary material for: Tomato genomic prediction for good performance under high-temperature and identification of loci involved in thermotolerance response
Source: Hortic Res. 2021 Oct 1;8:212. doi: 10.1038/s41438-021-00647-3 (PMC8484564; doi:10.1038/s41438-021-00647-3)
Supplement: Supplementary file 3 — Table S2 [file 41438_2021_647_MOESM3_ESM.pdf]

Table S2. Correlation coefficient (r) among variables. Asterisks show the significance level of the correlation at P<0.05. YP: yield production per plant; TFN: total fruit number per plant; SSC: Soluble solid content; CR: contemporaneous ripening; FS: percentage of fruit set; IN: inflorescence number; LC: leaf coverage; FRL: fruit earliness.

	**YP (Kg)**	**TFN**	**SSC**	**CR**	**FS**	**IN**	**LC**
**TFN**	0.89*						
**SSC**	-0.33*	-0.28*					
**CR**	-0.53*	-0.45*	0.27*				
**FS**	0.58*	0.60*	-0.17	-0.23*			
**IN**	0.38*	0.36*	-0.30*	-0.24*	0.28*		
**LC**	0.14	0.16	-0.05	-0.46*	-0.06	-0.02	
**FRL**	0.33*	0.23*	-0.35*	-0.05	0.16	0.27*	-0.06
