# Supplementary material for: Tomato genomic prediction for good performance under high-temperature and identification of loci involved in thermotolerance response
Source: Hortic Res. 2021 Oct 1;8:212. doi: 10.1038/s41438-021-00647-3 (PMC8484564; doi:10.1038/s41438-021-00647-3)
Supplement: Supplementary file 4 — Table S3 [file 41438_2021_647_MOESM4_ESM.pdf]

Table S3. The transitions/transversions (Ts/Tv) ratio and total number of Ts and Tv in JAGF4 and JAGF5 populations were reported.

Population	Ts/Tv	Total no of Ts	Total no of Tv
JAGF4	1.19	73,392	61,863
JAGF5	1.21	96,71	79,886
