# Supplementary material for: Tomato genomic prediction for good performance under high-temperature and identification of loci involved in thermotolerance response
Source: Hortic Res. 2021 Oct 1;8:212. doi: 10.1038/s41438-021-00647-3 (PMC8484564; doi:10.1038/s41438-021-00647-3)
Supplement: Supplementary file 5 — Table S4 [file 41438_2021_647_MOESM5_ESM.pdf]

Table S4. SNP number obtained after filtering by PEMV for SSC and YP traits

PEMV	Number of markers	
	SSC	YP
90	14.286	14.210
85	16.625	16.224
80	18.806	18.637
75	21.019	20.555
70	23.471	23.228
