# Supplementary material for: Tomato genomic prediction for good performance under high-temperature and identification of loci involved in thermotolerance response
Source: Hortic Res. 2021 Oct 1;8:212. doi: 10.1038/s41438-021-00647-3 (PMC8484564; doi:10.1038/s41438-021-00647-3)
Supplement: Supplementary file 6 — Supplementary Figures [file 41438_2021_647_MOESM6_ESM.pdf]

Supplementary figure 1

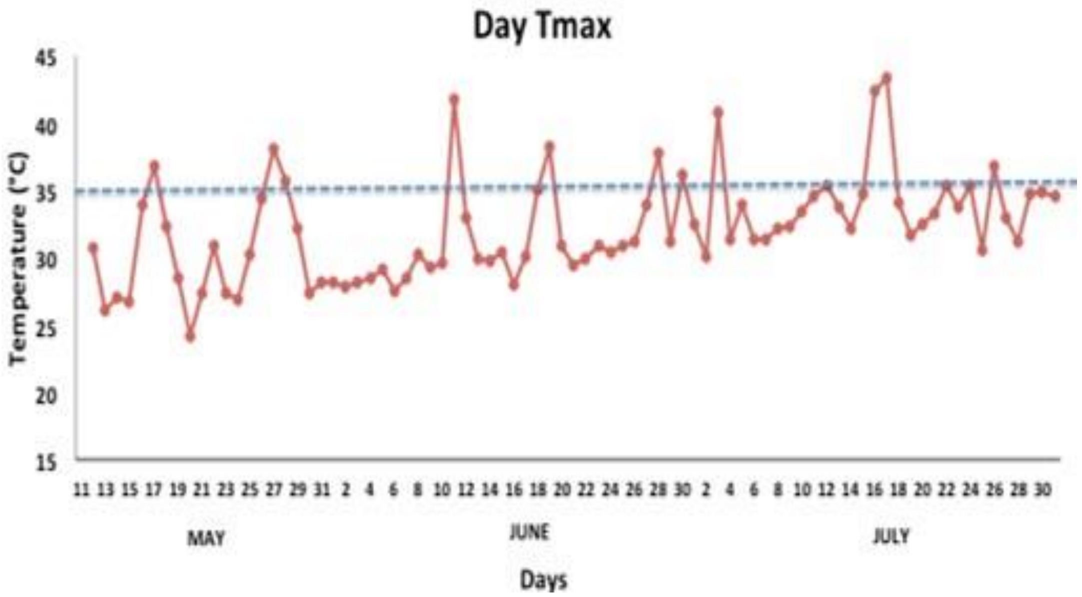

Maximum temperatures recorded in the experimental fields (Battipaglia, It) during the day from May to July 2017.

Supplementary figure 2

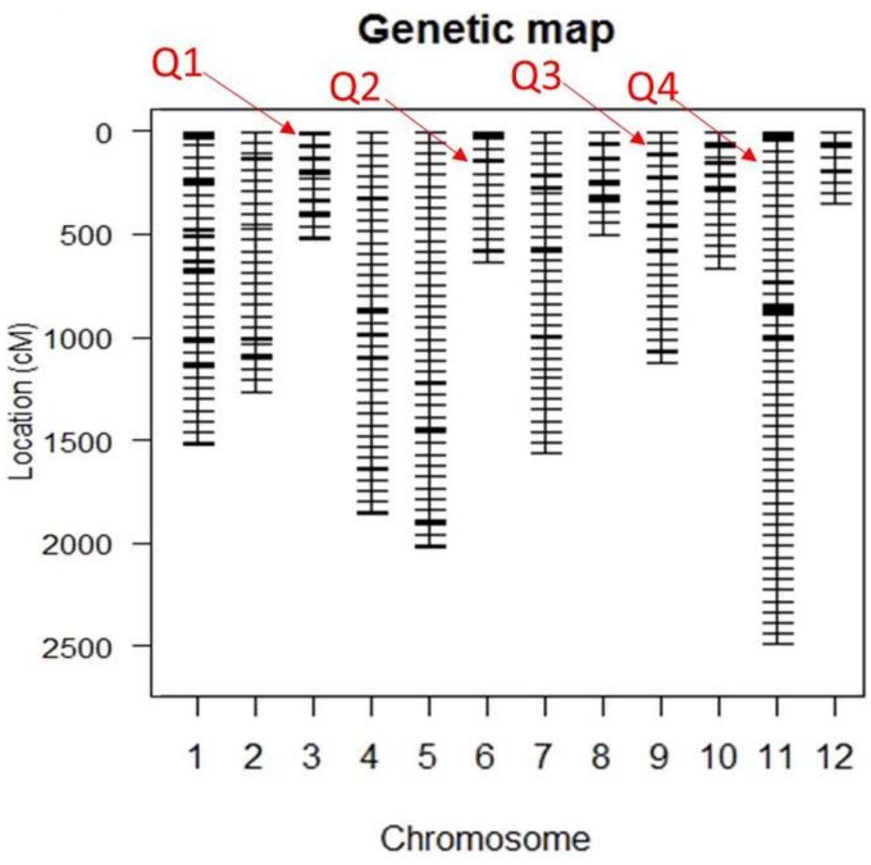

Yield QTLs on genetic map. The red arrows indicate the 4 identified QTLs on chromosome 3(Q1), chromosome 6(Q2), chromosome 9(Q3), and chromosome 11 (Q4).

Supplementary figure 3

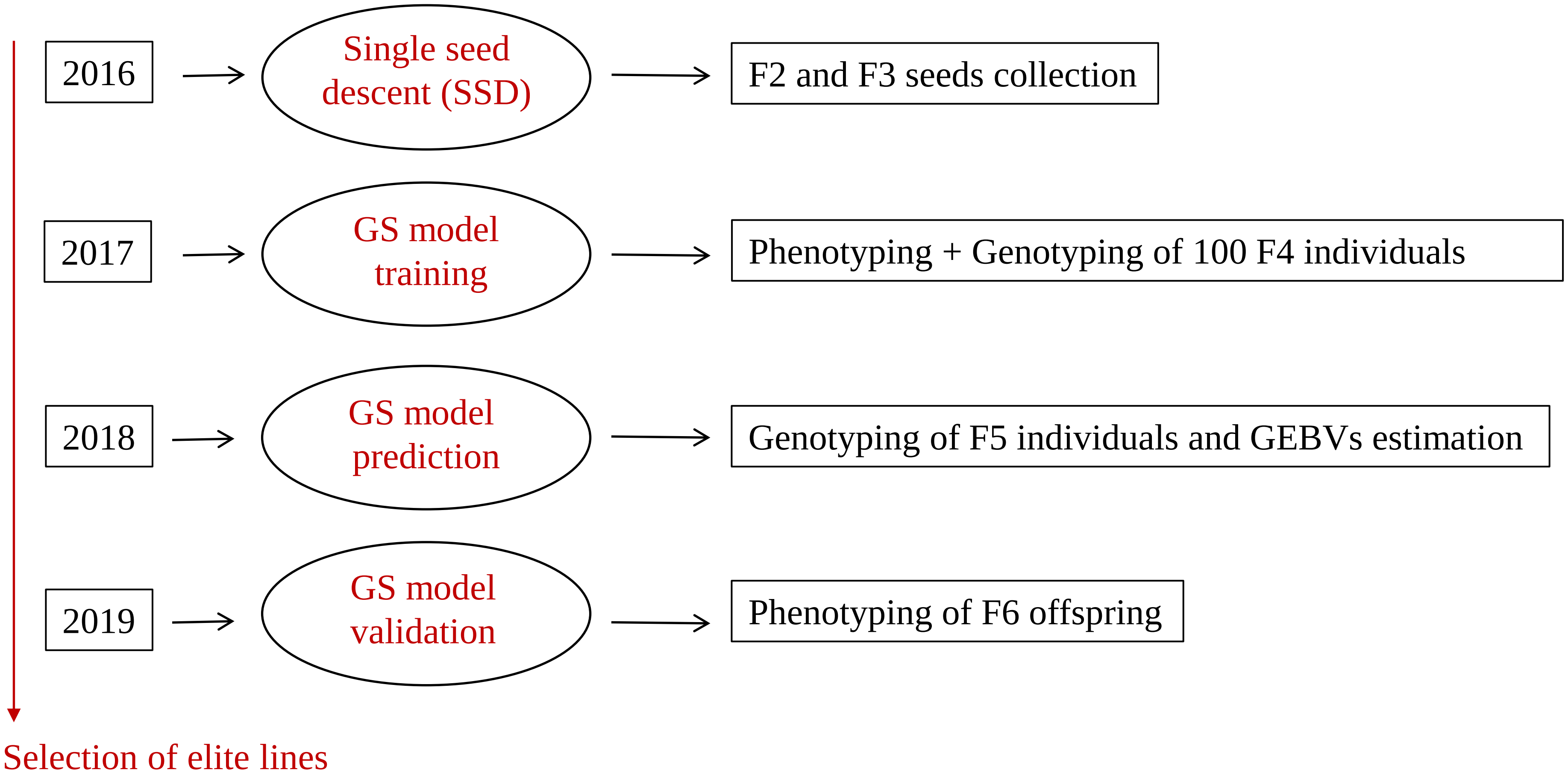

Genomic selection experimental scheme
